# Advancing Prognosis Prediction and Immunotherapy Efficacy in Lung Adenocarcinoma Through Machine Learning: Novel Insights From Anoikis Regulator Patterns in Single‐Cell Multiomics

**DOI:** 10.1155/ijog/9458552

**Published:** 2026-01-03

**Authors:** Shan Li, Wenhang Zhou, Chen Hu, Ting Chen, Jinping Li, Pengpeng Zhang

**Affiliations:** ^1^ Department of Laboratory Medicine, Northern Jiangsu People′s Hospital, Yangzhou, Jiangsu, China, yzsbh.com; ^2^ Department of Urology, Children′s Hospital of Chongqing Medical University, Chongqing, China, chcmu.com; ^3^ Department of Oncology, The Affiliated Huai′an Hospital of Xuzhou Medical University, the Second People′s Hospital of Huai′an, Huai′an, Jiangsu, China, xzmc.edu.cn; ^4^ Department of Anesthesiology, Huai′an Hospital of Huai′an City, Huai′an, Jiangsu, China

**Keywords:** anoikis, LUAD, machine learning, multiomics analysis, prognostic prediction, scRNA-seq, stRNA-seq

## Abstract

**Introduction:**

Anoikis, a type of programmed cell death induced by detachment from the extracellular matrix (ECM), is crucial in cancer progression. Resistance to anoikis often correlates with enhanced invasion, metastasis, treatment resistance, and tumor recurrence. However, no research has systematically explored anoikis‐regulated tumor microenvironment (TME) in lung adenocarcinoma (LUAD).

**Methods:**

We used single‐cell RNA sequencing (scRNA‐seq) and spatial transcriptome RNA sequencing (stRNA‐seq) analyses to reveal the subtype of anoikis‐related epithelial cells, demonstrating its spatial location characteristics. With the maker genes of prognostic significance, we depicted the molecular landscapes of anoikis regulator patterns in RNA‐seq data. We developed the anoikis‐related signature (Anoikis.Sig) by integrating 10 machine learning (ML) algorithms to accurately predict prognosis in LUAD. Based on the median risk score computed by Anoikis.Sig, patients were divided into high‐ and low‐risk groups. We employed extensive analysis between two risk groups, in terms of clinic implications, immune microenvironment, somatic mutations, immunotherapy, chemotherapy, and single‐cell landscape. Finally, we verified the prognosis value of two Anoikis.Sig model genes.

**Results:**

By integrative analysis of scRNA‐seq and stRNA‐seq datasets, we defined diverse function subtypes of anoikis‐related epithelial cells and investigated their spatial regulator patterns. Through its marker genes and leave‐one‐out cross‐validation, we utilized the RSF algorithm to develop the Anoikis.Sig with a superior predictive ability, outperformed other LUAD signatures and clinical indicators. We categorized LUAD patients into high‐ and low‐risk groups, which demonstrated the low‐risk group had a better survival outcome, an ample immune infiltration, a distinct mutational landscape, and response to immunotherapy. ScRNA‐seq analysis revealed biologically intercellular disparities delineated by Anoikis.Sig. qRT‐PCR validated the prognostic value of two model genes of Anoikis.Sig in LUAD.

**Conclusion:**

Through multiomics analyses and ML algorithms, we succeeded in establishing the Anoikis.Sig to efficiently predict prognosis in Anoikis.Sig, which delineated molecular landscapes of anoikis regulator patterns and clinical applications of Anoikis.Sig.

## 1. Introduction

Lung cancer is the most prevalent malignant tumor in the respiratory system and the primary reason of cancer‐related mortality among both genders [1]. According to the WHO classification, lung malignancies are categorized into nonsmall cell lung cancer (NSCLC) and small cell lung cancer (SCLC), with NSCLC constituting roughly 85% of cases and SCLC making up the remaining 15% [2]. Among these, adenocarcinoma and squamous cell carcinoma are the predominant subtypes of NSCLC, with lung adenocarcinoma (LUAD) being the most frequent, representing approximately 40% of all lung cancer diagnoses [3]. While surgical intervention remains the cornerstone treatment for early stage LUAD, its invasive characteristics usually complicate complete resection of tumors. The emergence of immunotherapy has revolutionized LUAD treatment, with immune checkpoint inhibitors notably enhancing patient prognosis and providing a neoadjuvant treatment for early stage, resectable tumor. However, despite the developing advancements, the 5‐year survival rate for LUAD patients was disappointingly low [4]. This underscores the critical need to uncover the molecular underpinnings of LUAD and establish robust molecular discrimination models to accurately predict prognosis and tailor individual therapy approaches.

Programmed cell death (PCD), or regulated cell death, is an essential physiological process vital for preserving tissue homeostasis and removing defective or unwanted cells. This process could unfold by a variety of mechanisms, such as apoptosis, anoikis, autophagy, alkaliptosis, cuproptosis, entosis, immunogenic cell death, ferroptosis, lysosome‐dependent cell death, methuosis, necroptosis, NETosis, oxeiptosis, pyroptosis, parthanatos, and paraptosis. Each of these mechanisms represents a distinct pathway for the regulated removal of cells, contributing to the overall health and stability of tissues [5]. Anoikis, one of the forms of PCD, is triggered when cells detach from the extracellular matrix (ECM) [6], resulting in the absence of integrin‐mediated survival signals and the activation of Bcl‐2 family members, which ultimately induce cell death [7]. While anoikis is essential for maintaining tissue homeostasis, cancer cells can sometimes evade this death pathway, developing resistance to anoikis. This resistance enables detached cells to endure in unfavorable circumstances [8]. Nowadays, studies are focused on elucidating the regulatory mechanisms of anoikis by diverse pathways, including integrins, epidermal growth factor receptor (EGFR), TGF‐*β* signaling, NF‐*κ*B signaling, and hypoxia [9]. One study highlighted that signals activated by *β*1 integrin are crucial for the formulation of anoikis resistance in glioblastoma cells [10]. Moreover, a glutaminolysis enzyme, Glutamate Dehydrogenase 1 (GDH1), upregulated with detachment by pleomorphic adenoma Gene 1 (PLAG1), could provide antianoikis and prometastatic signaling in LKB1‐deficient lung cancer [11]. Remarkably, the majority of existing treatment strategies, such as immunotherapies and cytotoxic drugs, have largely overlooked the crucial significance of anoikis. Currently, our understanding of how anoikis operates within the components of the tumor microenvironment is insufficient, which hinders the formulation of effective treatment regimens. It is imperative to conduct further research to deepen our knowledge of these associations and establish a solid foundation for the development of powerful clinical interventions.

Existing prognostic models, such as the TNM staging system, primarily rely on clinical and pathological features. While these tools have been instrumental in classifying patients into broad risk categories, they often fail to capture the molecular heterogeneity inherent in LUAD. The medical research landscape is undergoing a significant transformation, driven by advanced bioinformatics tools. Innovations in RNA sequencing (RNA‐seq) analysis, genetic variation studies, and high‐resolution single‐cell RNA sequencing (scRNA‐seq) related research are revolutionizing our comprehension of illness mechanisms [12]. When applied to the study of prognosis prediction and immunotherapy efficacy in LUAD, these methodologies provide novel insights into potential therapeutic strategies. Currently, several molecular markers have emerged as important immunotherapy predictors and prognostic indicators for LUAD [13]. Indicators include PD‐L1 expression levels, tumor mutational burden (TMB), and hematological biomarkers, which are key predictors of treatment outcomes and closely linked to the prognosis of LUAD. In addition to genetic factors, various environmental and lifestyle factors also play a role in the development of LUAD. For instance, carcinogens in tobacco smoke can induce the differentiation of bronchial epithelial cells, thus facilitating tumorigenesis [14]. However, these traditional indicators were sometimes not powerful enough, possibly facilitating more novel prediction tools to emerge as reliable assistants in enhancing prognosis prediction and immunotherapy response in LUAD. Harnessing diverse machine learning (ML) methods [15], we aimed to develop a novel signature based on anoikis‐related cellular patterns to evaluate immunotherapy effectiveness and forecast patient prognosis, through covering massive multiomics data of LUAD patients.

## 2. Materials and Methods

### 2.1. Sequencing Cohorts

We totally obtained 10 transcriptome datasets of LUAD: GSE13213, GSE26939, GSE29016, GSE30219, GSE31210, and GSE42127 in GEO, TCGA‐LUAD in TCGA, and three cohorts (OAK dataset [16], POPLAR dataset [17], and Jung cohort [18]) containing immunotherapy clinical information. We used the log2 (*x* + 1) method to normalize transcriptome reads and employed the combat method from the “sva” R package to decrease batch influence [19]. Protein expression data of LUAD was retrieved from the CPTAC database. Moreover, we acquired two scRNA‐seq cohorts of LUAD (GSE189357 and GSE131907) from GEO. For pancancer bulk RNA‐seq data of immunotherapy, GSE91061, GSE135222, GSE78220, and IMvigor datasets were acquired for analysis. Anoikis‐associated genes were retrieved from the Molecular Signatures Database (MSigDB, https://www.gsea-msigdb.org/gsea/msigdb).

### 2.2. Single‐Cell Analysis of Anoikis Regulator

For the scRNA‐seq matrix, raw mRNA expressions were read into the Seurat object through the Seurat R package (Version 4.4.0) [20]. We deleted unqualified cells with > 40,000 UMI per cell, < 500 genes per cell, > 5000 genes per cell, and > 20% mitochondrial genes. We reduced doublets through the “DoubletFinder” R package [21]. We harnessed the “Harmony” R package to decrease batch influence between scRNA‐seq datasets [22]. We implemented the FindVariableFeatures method to reveal the Top 2000 variant genes [23], at which PCA was employed. We implemented the FindClusters method to find differential clusters at a resolution of 0.5. We conducted the RunUMAP method to decrease dimension and manually annotated cellular subtypes according to canonical markers [24]. Acquisition of marker genes of epithelial subpopulations was conducted by the standard of log2FC > 0.25 and *p* value < 0.05 through the FindAllMarkers method. ScRNA‐seq score methods (AUCell in “AUCell” R package, Ucell in “Ucell” R package, ssGSEA and GSVA in “GSVA” R package, singscore in “singscore” R package, and AddModuleScore and PercentageFeatureSet in “Seurat” R package) were applied to acquired enrichment scores in single cells [20, 25–27], which were mentioned in our previous study [28]. Function‐enriching analysis through GO and KEGG was employed by the “ClusterGVis” R package (https://github.com/junjunlab/ClusterGVis). A numeric vector indicating cellular differentiation status, ranging from least differentiated (1.0) to most differentiated (0.0), was generated from the RNA matrix using the “CytoTRACE” R package [29]. We used “slingshot,” “Monocle,” and “Monocle 3” R packages to deduce cell lineages and pseudotime states [30–32]. We employed the “Scissor” R package to investigate the specific cellular subpopulations contributing to prognosis disparities by aggregating sequencing information and phenotype information [33]. We employed the “inferCNV” R package to define CNVs of epithelial cells, referred to as endothelial cells [34]. The “CellChat” R package was employed to investigate cellular interplay networks [35]. And we used “pySCENIC” (Version 0.11.2) in Python (Version 3.7) to investigate enriching transcription factors and regulon functionality of every cell state [36].

### 2.3. Spatial Transcriptome Analysis

To investigate the cellular overview of epithelial subtypes in a spatial transcriptome RNA‐seq (stRNA‐seq) viewpoint, we obtained stRNA‐seq data of LUAD from the GEO database with Accession Number GSE189487 [24]. Spatial transcriptome data analysis was performed via the “Seurat” R package. To precisely obtain the cellular compositions in every spot of stRNA‐seq data, a deconvolution method was employed [37], aggregating paired or unpaired stRNA‐seq and scRNA‐seq datasets to obtain the cellular compositions. Visualization of deconvolution results of various cell types was conducted by the “STdeconvolve” R package. To guarantee the accuracy, we implemented stringent quality control procedures on scRNA‐seq data, which was based on multiple criteria including the number of expressed genes, unique molecular identifier (UMI) counts, and proportion of mitochondrial RNA. Following the quality control process, we developed a signature score matrix. This was achieved by averaging the expressions of the Top 20 genes that were specifically expressed in each cell type, using the scRNA‐seq reference data from each site. We utilized the AddModuleScore method to compute enriching scores of every cellular subtype. These scores were then illustrated with the SpatialFeaturePlot method from the “Seurat” R package. In order to determine the spatial coordinates of different subtypes, we conducted a joint analysis of scRNA‐seq and stRNA‐seq datasets through the CellTrek R package [38], which was mentioned in our previous study [28]. The run_kdist method was utilized to measure the spatial *k*‐distance among various epithelial subpopulations and cell types. To analyze the intensity of spatial communication among different cell subtypes, we adopted the approach described in the “stLearn” (Version 0.3.2) method with Python (Version 3.7) [39].

### 2.4. Development of Anoikis‐Related Signature (Anoikis.Sig) by Integrating ML Algorithms

The TCGA‐LUAD cohort was selected as the training set for the development of the prognostic model. Meanwhile, the other six cohorts were designated as validation sets, while these validation cohorts were pooled together to form a comprehensive test set. By leveraging the marker genes of anoikis‐associated epithelial subtypes that possess prognostic significance, we created the Anoikis.Sig to predict the prognosis of patients with LUAD. We integrated 10 prognosis ML algorithms, covering random survival forest (RSF), elastic network (Enet), LASSO, Ridge, stepwise Cox, CoxBoost, partial least squares regression for Cox (plsRcox), supervised principal components (SuperPC), generalized boosted regression modeling (GBM), and survival support vector machine (survival‐SVM) for prognosis prediction. Totally 101 prognosis ML algorithms, which were described in our previous study [28], were trained in the training cohort through a leave‐one‐out cross‐validation (LOOCV) system to establish the Anoikis.Sig. Signatures with fewer than five genes were excluded. The average concordance index (C‐index) was obtained in each ML combination across the datasets [15]. The prognosis ML system scoring the best average C‐index was decided as the optimal signature. Risk scores were computed by the linear combination method for each prognosis ML combination through including model gene expressions. Using a single‐cell scoring algorithm and Cox regression analysis, we identified risky and protective genes in LUAD and computed the Anoikis.Sig score in scRNA‐seq data based on the following formula: Anoikis.Sig risk score = Algorithm_Score (risky genes) − Algorithm_Score (protective genes).

### 2.5. Model Validation of Anoikis.Sig

Extensive validation procedures were employed to verify the outstanding accuracy, stability, and discrimination of Anoikis.Sig. Logarithmic loss, recall, and decision calibration were employed to elect the optimal prognostic ML framework using the “mlr3proba” R package [40]. LUAD patients were separated into high‐ or low‐risk groups with the median Anoikis.Sig risk score in the training cohort. Verifications were carried out by Kaplan–Meier (KM) survival analysis and log‐rank test, through the “survival” and “survminer” R packages. Receiver operating characteristic (ROC) curves, calibration curves, and decision curve analysis (DCA) were applied to appraise the accuracy, discrimination, and clinical utility of Anoikis.Sig. Following this, we evaluated and contrasted the predictive ability of our signature against traditional clinical characteristics by means of time‐dependent AUC values. Additionally, we employed univariate and multivariate Cox regression analyses to ascertain the independent prognostic value of Anoikis.Sig.

### 2.6. Quantitative Real‐Time PCR (qRT‐PCR) Analyses

Total RNA from tumor and normal tissues was extracted by TRIzol (Invitrogen). Total RNA was reverse transcribed with 4× Reverse Transcription Master Mix (EZBioscience, United States) under the manufacturer′s instructions. qRT‐PCR was employed with 2× SYBR Green qPCR Master Mix (EZBioscience, United States) and a Roche LightCycler 480 Instrument. The forward and reverse primers used for GAPDH are GCACCGTCAAGGCTGAGAAC and TGGTGAAGACGCCAGTGGA. The forward and reverse primers used for LDHA are ACCACGCACTTCTCATCTGAGC and GTGAGGGTGCGTAGCACAGC. The forward and reverse primers used for KRT7 are CGGCATCATCGCTGAGGTCAA and GCCTGGAGGGTCTCAAACTTGG.

## 3. Results

### 3.1. Single‐Cell Transcriptome Atlas of LUAD

Combining sequencing data of two scRNA‐seq cohorts (GSE189357 and GSE131907), we finally retrieved 159,588 cells after “harmony” batch effect reduction (Supporting Information 1: Figure S1A), with qualified quality control (Supporting Information 1: Figure S1B). With canonical cell markers, we manually defined 13 major cell types, covering epithelial cells, T cells, B cells, NK cells, NKT cells, endothelial cells, macrophages, monocytes, plasma cells, fibroblasts, mast cells, neutrophils, and dendritic cells (DCs) (Figure 1a), with LUAD cells sourced from two scRNA‐seq cohorts (Figure 1b). We illustrated the Top 5 marker genes of every major cell type (Figure 1c). And we also harnessed GO and KEGG terms to functionally annotate each major cell type, which again validated our single‐cell annotation (Figure 1d). Several typical marker genes of each major cell type were displayed in a dot plot (Figure 1e). We then revealed the different cell proportions in every patient, demonstrating the tumor heterogeneity of LUAD (Figure 1f). Moreover, the expressions of anoikis‐related genes were visualized in a heatmap, showing their molecular landscapes in LUAD (Supporting Information 1: Figure S1C).

Figure 1Comprehensive scRNA‐seq landscapes of major cell types in LUAD. (a) Visualizing the distribution of various cell populations in the TME of LUAD by UMAP plot. (b) Visualizing the distinct LUAD cells from two scRNA‐seq cohorts. (c) Visualizing each major cell type′s Top 5 marker genes. (d) Visualizing each cell type′s marker genes with a heatmap, as well as GO and KEGG enrichment analysis, showed each cell type′s top functional terms. (e) Canonical marker genes used to annotate the major cell types. (f) The different cell proportions in different LUAD patients.

**Figure:**
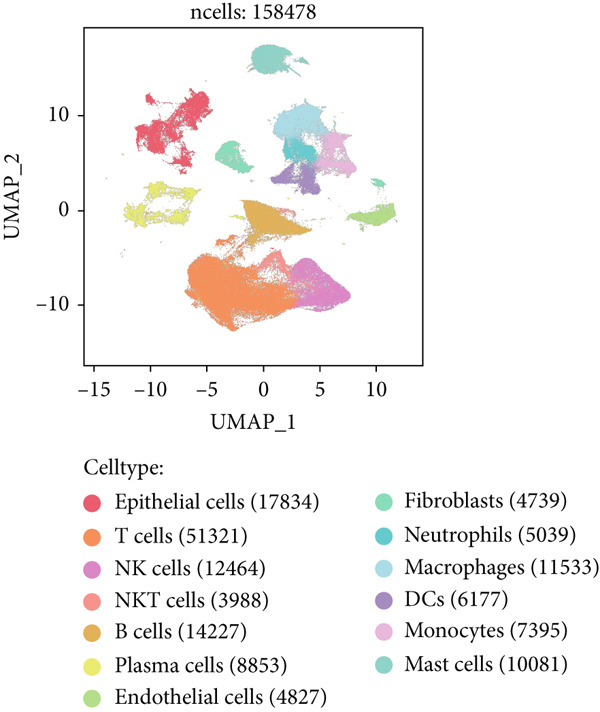
(a)

**Figure:**
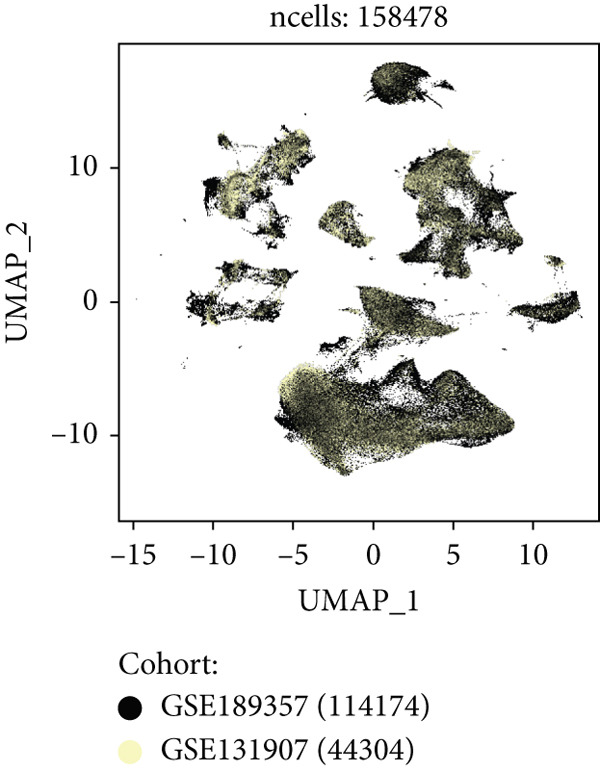
(b)

**Figure:**
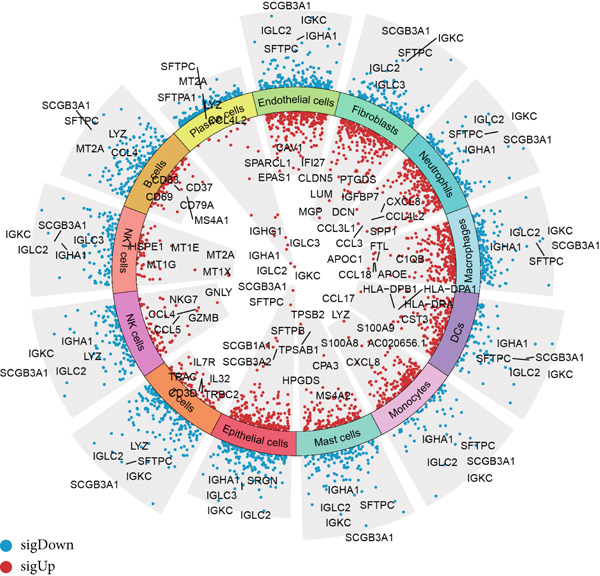
(c)

**Figure:**
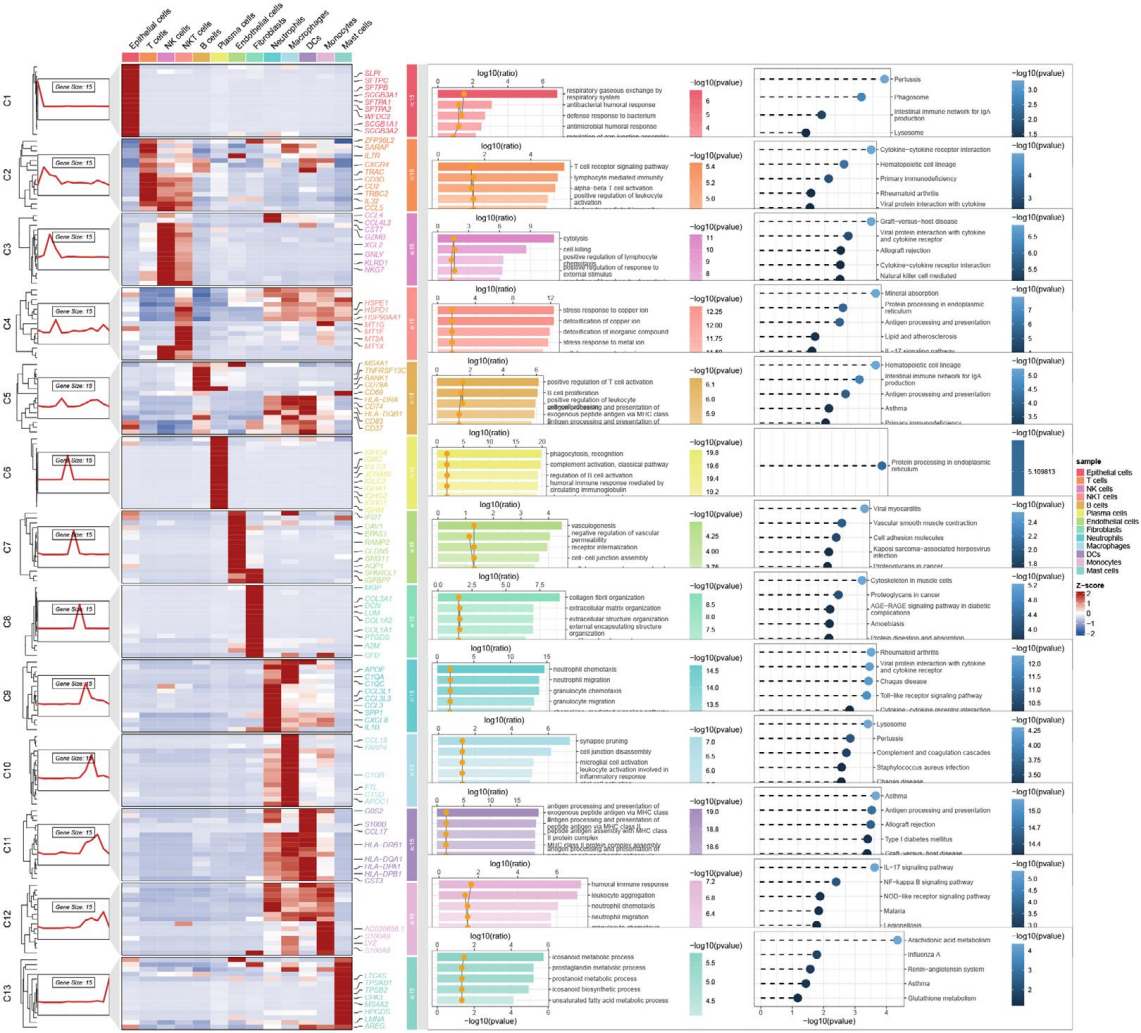
(d)

**Figure:**
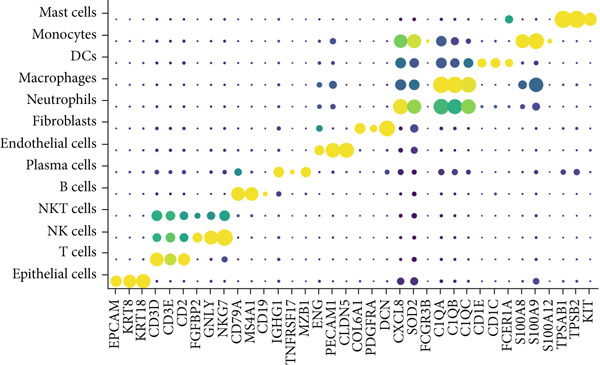
(e)

**Figure:**
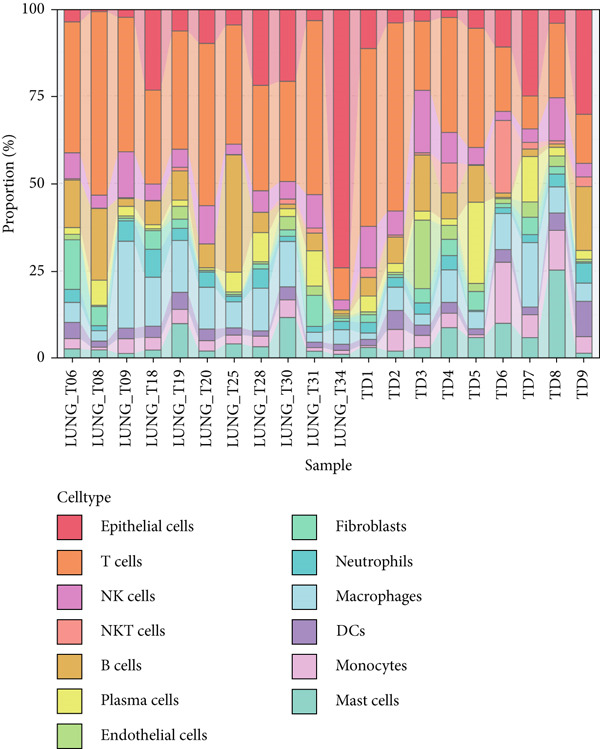
(f)

### 3.2. Epithelial Heterogeneity and Identification of Anoikis‐Related Cells

To demonstrate the molecular landscapes of anoikis in the comprehensive scRNA‐seq atlas of LUAD, we conducted scRNA‐seq enrichment analysis with the anoikis gene set to retrieve the anoikis‐enriched scores in LUAD cells (Figure 2a,b). The results indicated that the epithelial cells and endothelial cells scored the highest anoikis‐enriched scores, revealing the underlying abundant anoikis‐related biological functions in these two cell types. Given the oncological functions of epithelial cells in LUAD, we next extracted epithelial cells for deep investigation and performed further annotation on epithelial cells. We utilized the “harmony” algorithm to reduce batch effect in epithelial cells between two scRNA‐seq cohorts (Supporting Information 1: Figure S1D). Setting the resolution at 0.5, we finally identified 12 various clusters in epithelial cells in LUAD (Figure 2c). Subsequently, we performed “inferCNV” analysis to define the possible malignant epithelial cells in LUAD, which demonstrated that Clusters 0, 2, 3, and 7 had the most mutations among epithelial cells (Figure 2d). So based on the mutation results and marker genes retrieved from previous literature [24] (Supporting Information 2: Figure S2A,B), we manually annotated the epithelial cells into 10 subgroups (“AT2‐like Epi,” “ciliated‐like Mal,” “CRABP2+ Mal,” “AT1,” “lymphoid‐like Epi,” “Clara‐like Epi,” “myeloid‐like Epi,” “VIM+ Epi,” “TM4SF1+ Mal,” and “Clara‐like Mal,” Figure 2e), with “AT2‐like Epi,” “ciliated‐like Mal,” “TM4SF1+ Mal,” and “Clara‐like Mal” being the possible malignant epithelial cells. After successful identification of epithelial subgroups, we sought to discover the anoikis‐related cell subpopulation, by which we performed Cox regression analysis to identify risky and protective anoikis‐related genes in LUAD (Figure 2f). Then, according to the formula mentioned above, we conducted AddModuleScore‐, PercentageFeatureSet‐, AUCell‐, and ssGSEA‐based single‐cell scoring in epithelial cells, which successfully defined that AT2‐like Epi subpopulation was the most anoikis‐related cell subpopulations in epithelial cells (Figure 2g, Supporting Information 2: Figure S2C). Moreover, the expressions of anoikis‐related genes were illustrated in a heatmap, revealing their abundant molecular expression in AT2‐like Epi (Supporting Information 2: Figure S2E). Nevertheless, we implemented the CytoTRACE algorithm to reveal the heterogeneity of LUAD epithelial cells, illustrating that CRABP2+ Mal had the top CytoTRACE scores, inferring a possible stemness feature of this subpopulation, while AT2‐like Epi seemed to be in the final phase of the differentiation process (Figure 2h, Supporting Information 2: Figure S2D). With CRABP2+ Mal defined as the beginning, we performed Monocle 3 (Figure 2i, Supporting Information 3: Figure S3A) analysis and slingshot (Figure 2j) analysis to investigate differentiational lineages and calculate pseudotime scores among epithelial cell subpopulations, demonstrating the sophistication and heterogeneity of LUAD epithelial differentiation.

Figure 2Epithelial cell heterogeneity and identification of anoikis‐related epithelial cells in LUAD. (a, b) The AddModuleScore algorithm calculated the single‐cell scoring results of LUAD cells with the anoikis gene set. (c) Further downscaled clustering of epithelial cells using Seurat. (d) CNV landscapes of epithelial cells and total CNV of epithelial subgroups calculated by inferCNV. (e) Annotation of epithelial subgroups with distribution characteristics displayed by UMAP. (f) Univariate Cox regression analysis of anoikis‐related genes in the TCGA‐LUAD cohort. (g) AddModuleScore, PercentageFeatureSet, AUCell, and ssGSEA algorithm calculated the single‐cell scoring results of epithelial cells with the anoikis gene set. (h) CytoTRACE analysis assessing stemness among epithelial cell subpopulations. (i, j) The pseudotime trajectories from the (i) Monocle 3 analysis and (j) slingshot analysis reveal the differentiation paths of epithelial cell subpopulations.

**Figure:**
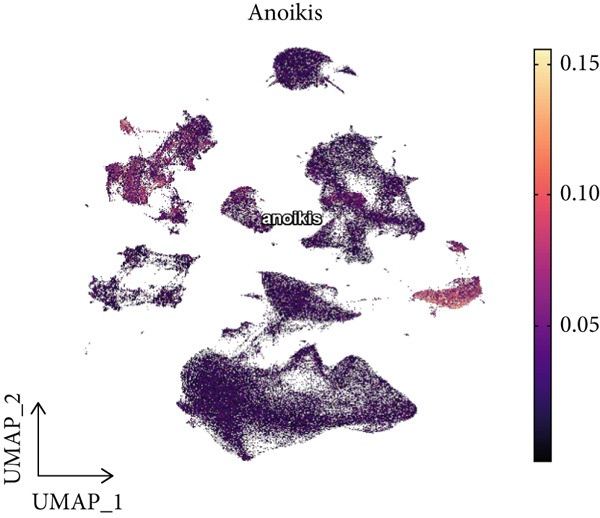
(a)

**Figure:**
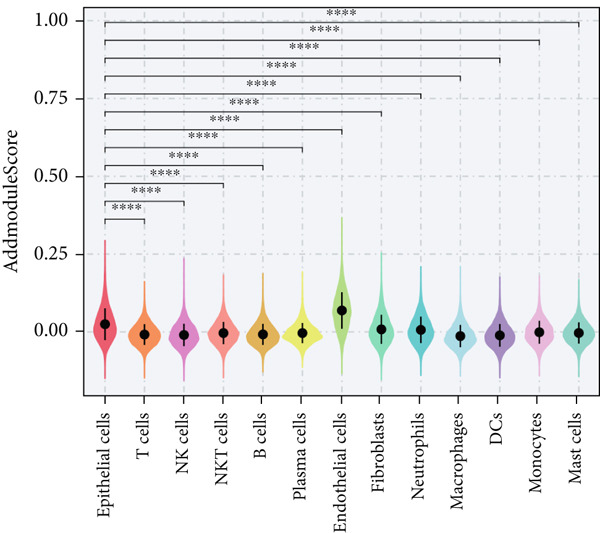
(b)

**Figure:**
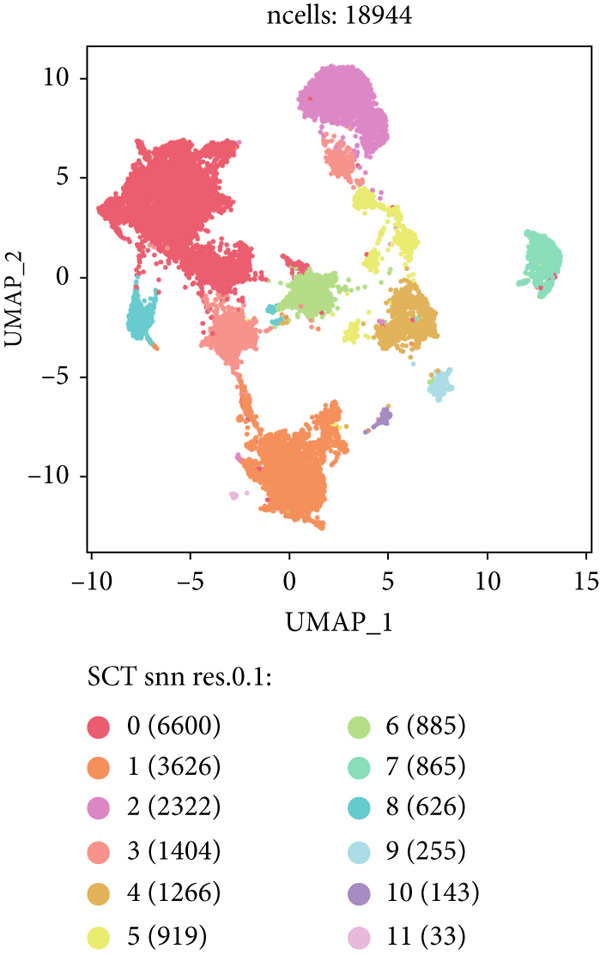
(c)

**Figure:**
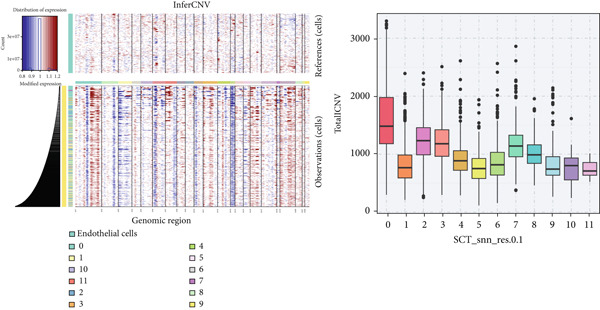
(d)

**Figure:**
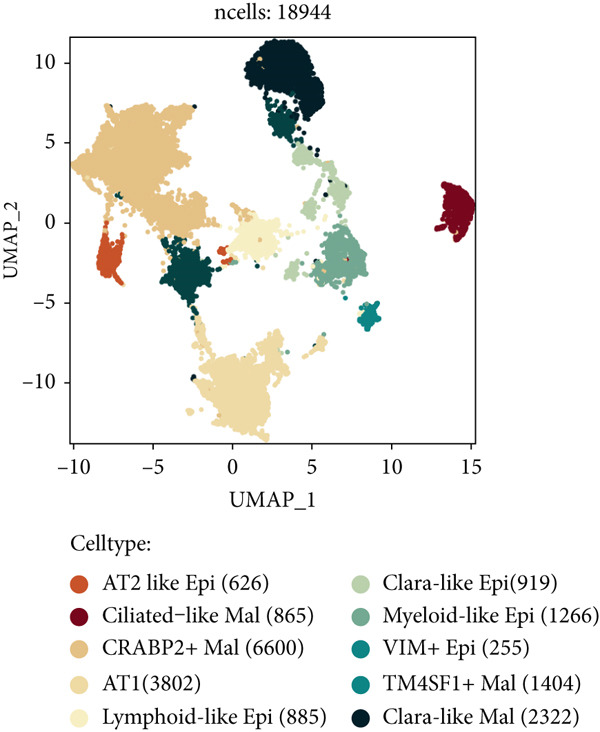
(e)

**Figure:**
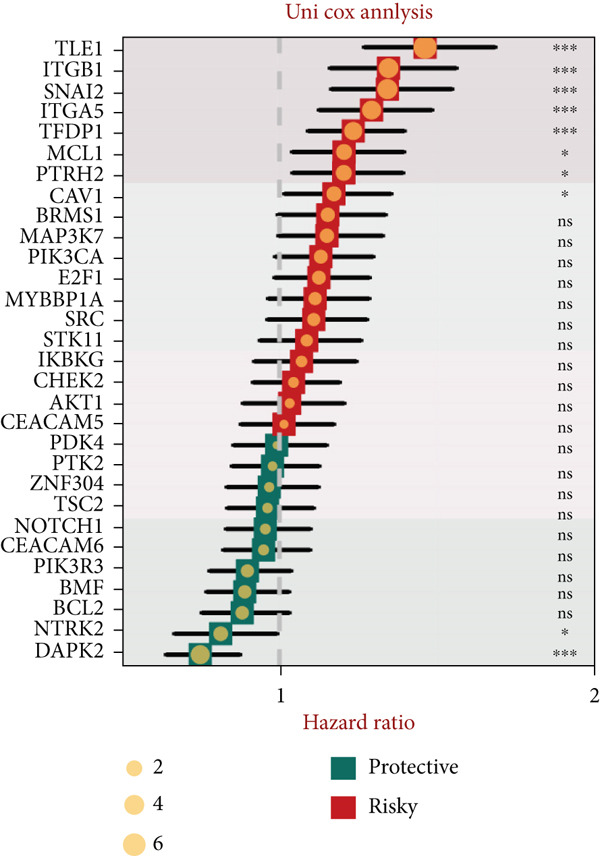
(f)

**Figure:**
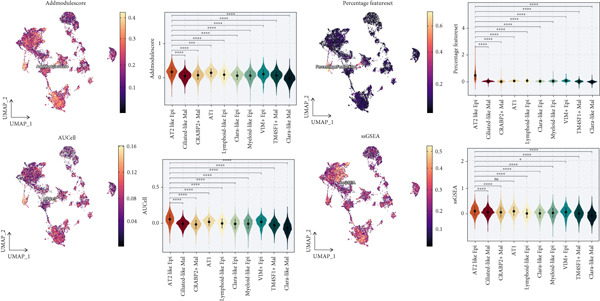
(g)

**Figure:**
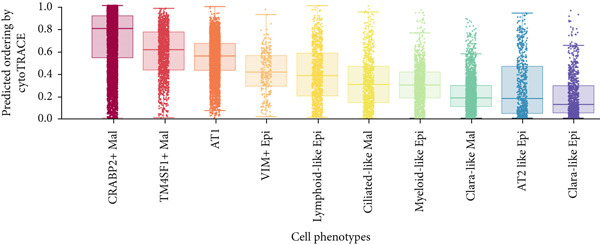
(h)

**Figure:**
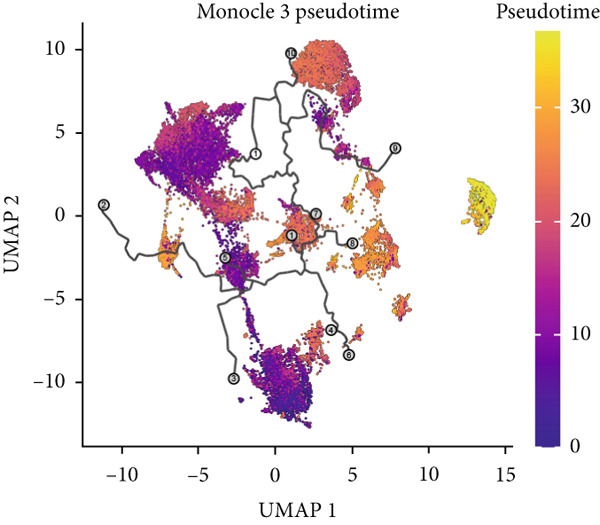
(i)

**Figure:**
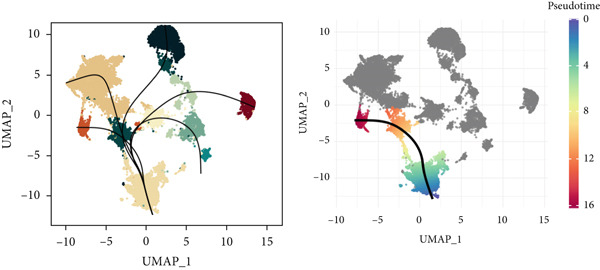
(j)

### 3.3. Elucidating the Molecular Landscapes of Epithelial Subgroups

To deeply analyze the comprehensive landscape of epithelial heterogeneity, we conducted the following scRNA‐seq analysis to reveal the biological functions of anoikis‐related subpopulations. We illustrated the Top 5 marker genes of each epithelial subtype (Figure 3a). Then, we harnessed GO and KEGG terms to functionally annotate each epithelial subtype, which provided biologically functional insights of every epithelial subtype (Figure 3b). We further revealed the different cell proportions in every patient, showing the epithelial heterogeneity of LUAD (Figure 3c). Besides, we performed Scissor analysis to prove the malignant roles of several epithelial subpopulations, which reveals that “AT2‐like Epi” and “CRABP2+ Mal” were Scissor+ cells with risky prognosis value (Figure 3d). Moreover, we also validated the malignant role of AT2‐like Epi in the TCGA‐LUAD cohort. Correlation analysis of dysfunction scores and AT2‐like Epi scores demonstrated that cell abundance of AT2‐like Epi was significantly related to immune dysfunction, indicating the probable mechanism of tumor immune escape in LUAD (Figure 3e). In the meantime, survival analysis of TCGA‐LUAD patients divided by median AT2‐like Epi score revealed that patients with higher AT2‐like Epi scores would suffer worse prognosis, indicating the malignant role of AT2‐like Epi (Figure 3e). While the median expressions of CD8A were not able to risk‐stratify TCGA‐LUAD patients in survival analysis, we interestingly discovered that within median CD8A expression and median AT2‐like Epi score, we could divide TCGA‐LUAD patients into four groups and reveal that patients with high CD8A expression and high AT2‐like Epi score suffered the worst prognosis, verifying the malignant behavior of AT2‐like Epi (Supporting Information 3: Figure S3B). Cell communication analysis among various cell populations displayed their intricate intercellular interactions, with almost all cell clusters exhibiting significant communication with AT2‐like Epi (Figure 3f). We plotted the cell interaction landscapes and demonstrated overexpressed ligand–receptor pairs and communication profiles among cell clusters and AT2‐like Epi (Supporting Information 3: Figure S3C). Distinct cell subtypes would produce dissimilar contributive cues affecting the total, inbound, and outbound signals, with macrophages and fibroblasts showing exceptional importance (Supporting Information 3: Figure S3D). And AT2‐like Epi could remarkably exhibit distinct signal output patterns, as well as differential signal input patterns (Supporting Information 3: Figure S3E). GSVA enrichment analysis within the Hallmark terms further elucidated the heterogeneity of tumor epithelial cells, with the AT2‐like Epi significantly enriched in heme metabolism (Supporting Information 3: Figure S3F). Finally, we investigated the distinctly enriched motifs across the epithelial subpopulations by SCENIC analysis (Figure 3g), with TEAD4, SP1, and SMAD9 motifs ranked highly in AT2‐like Epi.

Figure 3Molecular landscapes of various epithelial subgroups. (a) Visualizing each major cell type′s Top 5 marker genes. (b) Visualizing each cell type′s marker genes with a heatmap, as well as GO and KEGG enrichment analysis, showed each cell type′s top functional terms. (c) The different subgroup proportions in different LUAD patients. (d) Visualization of epithelial cells with risky prognosis value (Scissor+ cells) and epithelial cells with protective prognosis value (Scissor− cells) in LUAD. (e) The correlation between enrichment scores of AT2‐like Epi calculated by ssGSEA and dysfunction scores, while survival analysis of LUAD patients with high or low AT2‐like Epi scores. (f) Circle diagrams showed the interaction strength and numbers among two epithelial subpopulations, immune cells, and stromal cells. (g) SCENIC analysis indicated significant regulons and TF rank plots of each epithelial subpopulation.

**Figure:**
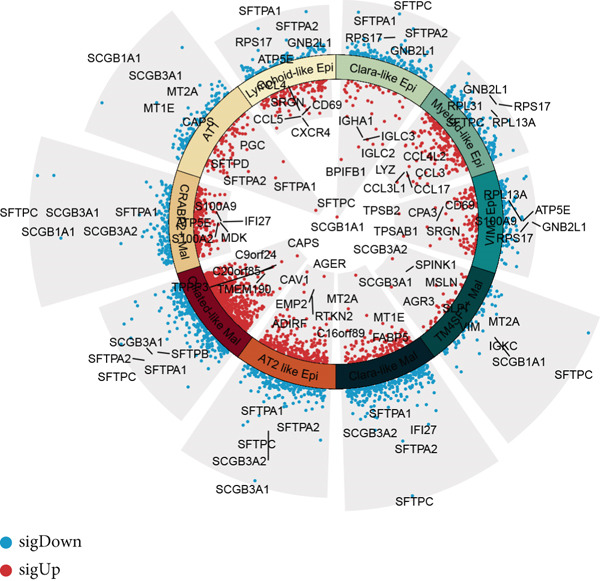
(a)

**Figure:**
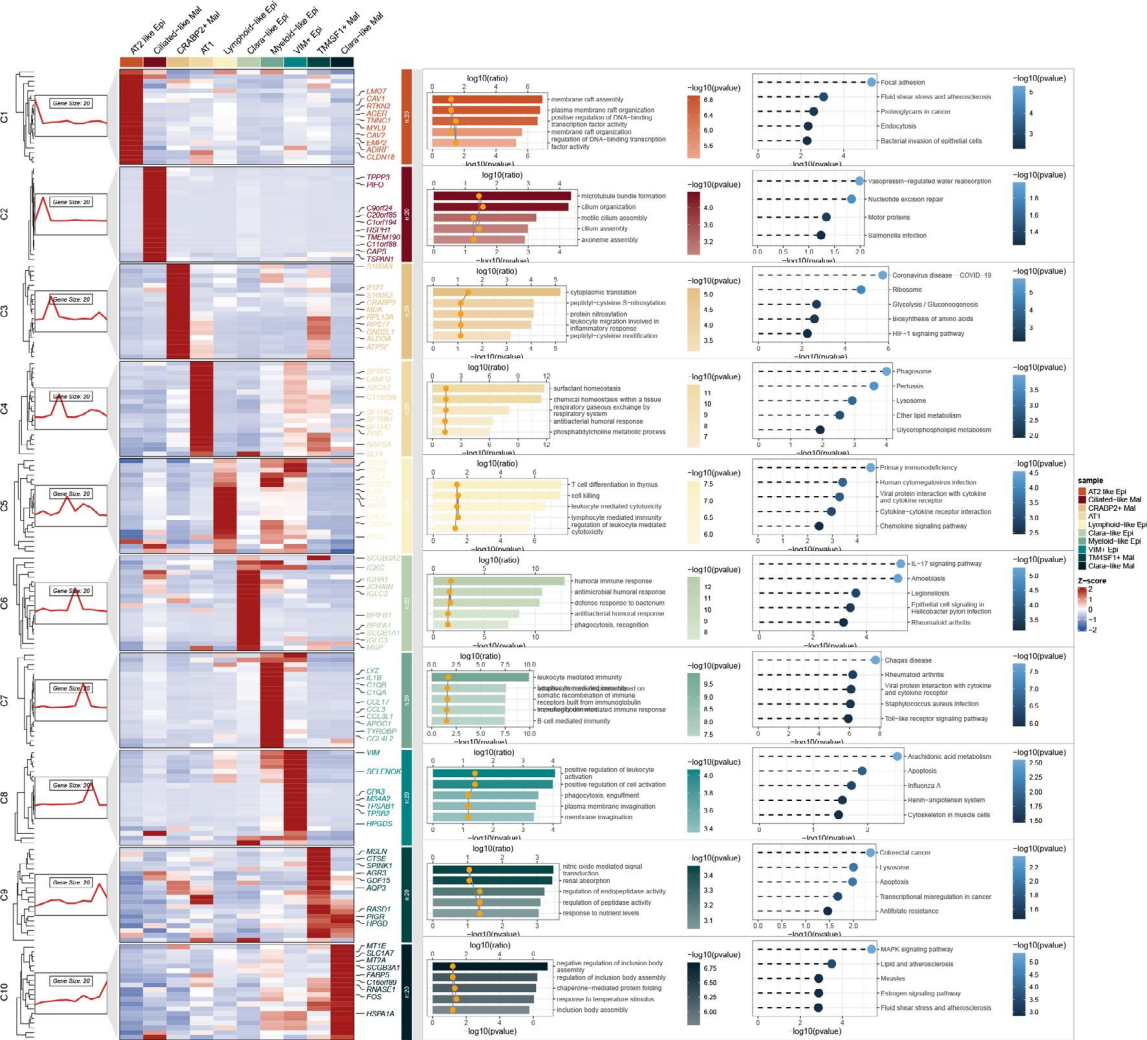
(b)

**Figure:**
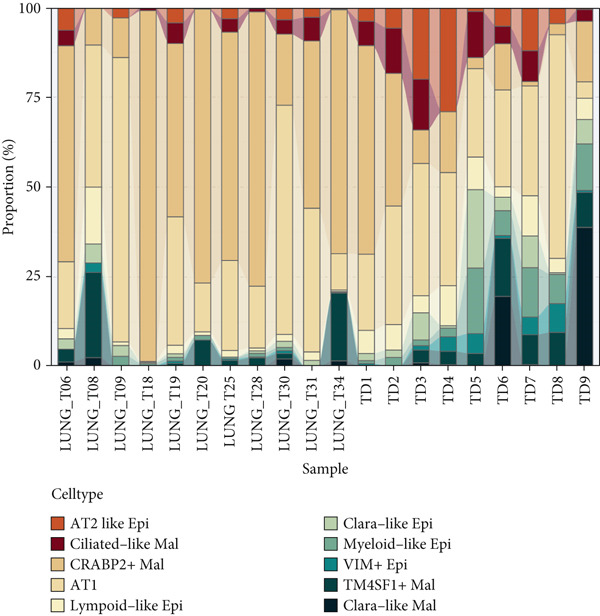
(c)

**Figure:**
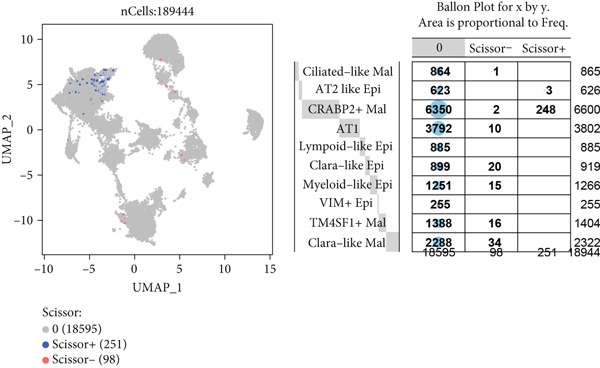
(d)

**Figure:**
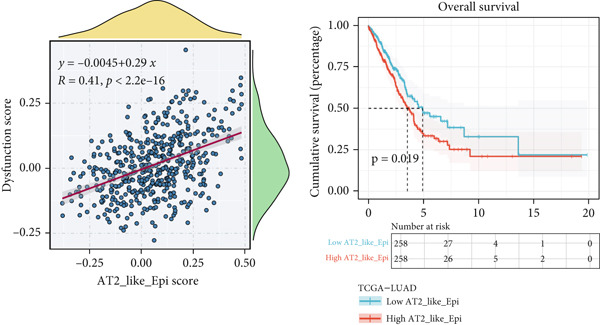
(e)

**Figure:**
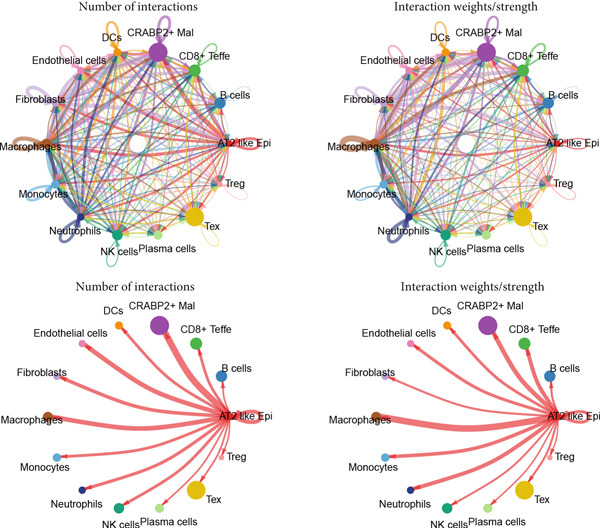
(f)

**Figure:**
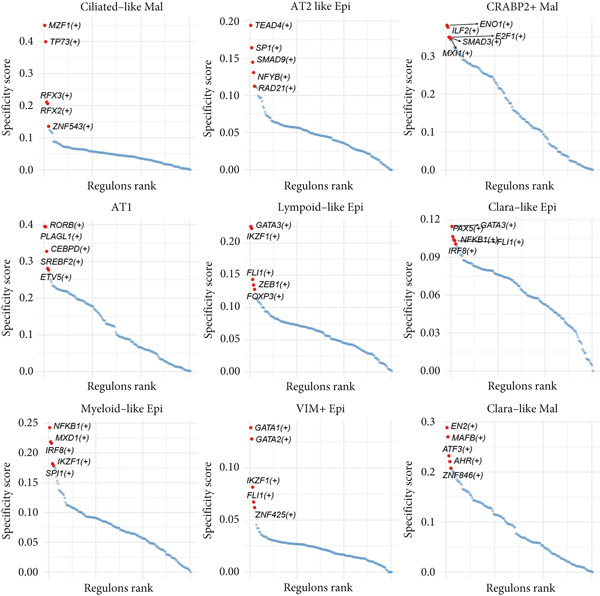
(g)

### 3.4. Spatial Distribution Characteristics of Epithelial Subtypes

Spatial transcriptome sequencing data of LUAD patients were sourced from the GSE189487 dataset. After excluding ribosomal and mitochondrial genes (Supporting Information 4: Figure S4A), the SCTransform method was applied to normalize sequencing depth and perform standardization steps. Through dimensionality reduction and clustering, 13 diverse cellular subtypes were defined within the spatial landscape (Figure 4a, Supporting Information 4: Figure S4B). Figure 4b displays the expression of 35 anoikis genes in epithelial subgroups, with CAV1 showing high expression in the LUAD section. We then visualized the possible spatial locations of various cell subpopulations by calculating enrichment scores with the AddModuleScore function. We used the Top 20 marker gene of various cell types obtained in scRNA‐seq data and performed spatial enrichment analysis of various cell types in LUAD sections, generally illustrating the spatial locations and abundance of various epithelial subgroups, stromal cells, and immune cells (Figure 4c). To deeply explore the spatial interactions of these cells, scRNA‐seq and stRNA‐seq data were integrated using RCTD deconvolution analysis to determine cell types and proportions in each spot (Figure 4d, Supporting Information 4: Figure S4C). Spots were defined according to the dominant cell type proportion in each section, with the proportions of various subtypes were visualized (Figure 4e). Besides, the spatial interplay among various cellular subtypes was inferred and illustrated. Notably, fibroblasts had stronger receptor–ligand (SERPING1_LRP1) interplay with AT2‐like Epi, while macrophages mostly engaged in communications with CRABP2+ Mal (Figure 4f). Furthermore, we used CellTrek on scRNA‐seq and stRNA‐seq data to build spatially single‐cell atlases (Figure 4g). We thereby revealed the spatial locations of epithelial subtypes in tissue sections by CellTrek mapping analysis, which showed an abundant infiltration of various epithelial subpopulations and immune, stromal cells in the tumor microenvironment (Figure 4g). Moreover, we computed the spatial *k*‐distance among various cell subtypes in tumor tissue sections, supporting that AT2‐like Epi subpopulation exhibited the minimum spatial *k*‐distance to endothelial cells, B cells, and NK cells (Figure 4g).

Figure 4Spatial transcriptomics analysis between epithelial cells, stromal cells, and immune cells in spatial coordinates. (a) Spatial map showing 13 clusters identified by stRNA‐seq. (b) Bubble chart presenting the expression of anoikis‐related genes in different clusters. (c) Spatial map showing the locations and abundances of various cell types in tumor sections calculated by the AddModuleScore function. (d) Identification of enriched cell types in each spot through deconvolution methods, with spatial maps showing the first abundant cell type in each spot. (e) Cell type proportions in each spot through deconvolution methods. (f) Heatmaps and network diagrams calculate the communication intensity between different cell types based on the stLearn method in the ST1 section. (g) Spatial cell charting of various cell subpopulations in each tissue section using CellTrek. And CellTrek calculated the average *k*‐distance from different cell types to the epithelial subtype in each tissue slice in the ST1 section.

**Figure:**
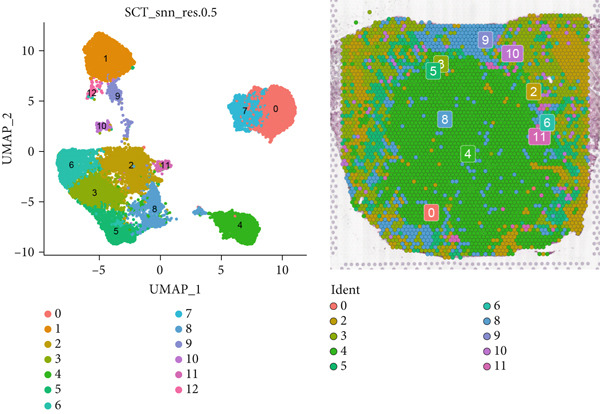
(a)

**Figure:**
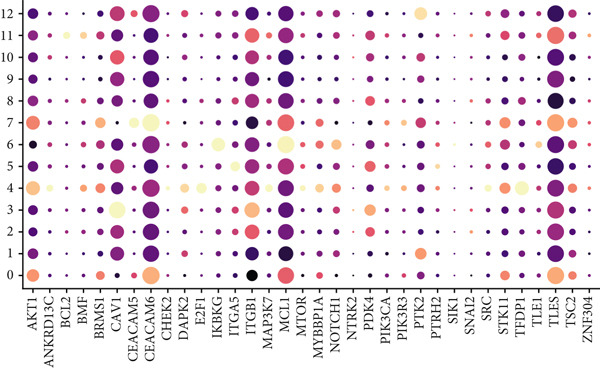
(b)

**Figure:**
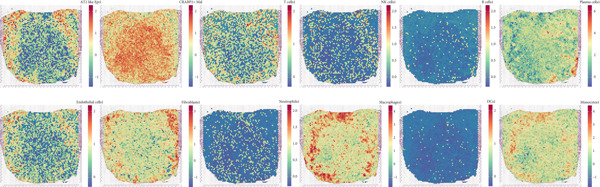
(c)

**Figure:**
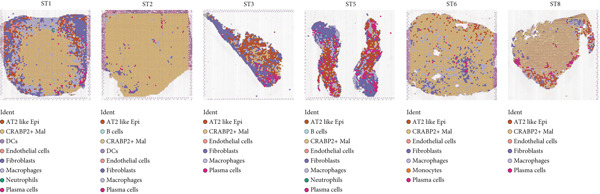
(d)

**Figure:**
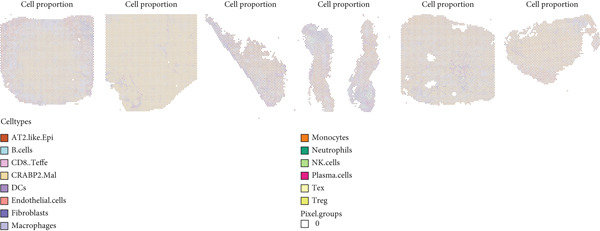
(e)

**Figure:**
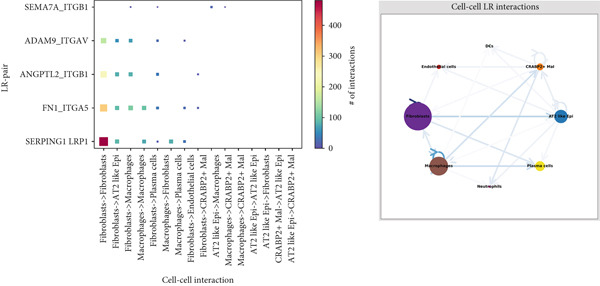
(f)

**Figure:**
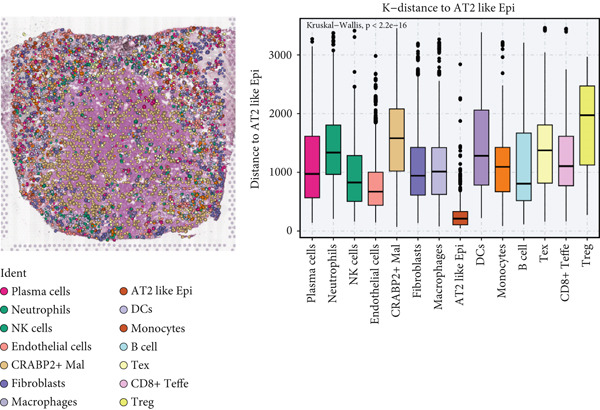
(g)

### 3.5. Development and Validation of Anoikis.Sig

We successfully removed most batch effects among seven LUAD cohorts before model development (Supporting Information 5: Figure S5A). Marker genes of AT2‐like Epi with prognosis value were used for feature selection and model development. We then conducted 101 prognosis ML combinations with an LOOCV framework to sift through the best ML combination to develop Anoikis.Sig (Supporting Information 5: Figure S5B). Considering the Top 5 ML frameworks within the best average C‐index, logarithmic loss, recall, and decision calibration were computed to appraise the calibration and exactness of ML models (Supporting Information 5: Figure S5C). Ultimately, the most effective prognosis ML combination was developed by RSF in feature selection and model development with the top average C‐index (0.691) in seven cohorts (Figure 5a). ROC curves in 5‐year OS (0.986 [0.975, 0.994] and 0.683 [0.671, 0.699]) of the train and the combined test cohorts demonstrated superior specificity of Anoikis.Sig (Figure 5b,c). Calibration curves showed the well accuracy and stability of Anoikis.Sig in the train and the combined test cohorts (Figure 5d,e), indicating that the model′s probability predictions were consistent and well‐calibrated. Survival analysis depicted that the low‐risk patients had a better overall survival (OS) than the high‐risk patients in the train and the combined test dataset (Figure 5f,g). AUC values of 3‐year OS displayed that Anoikis.Sig and Cox regression model covering Anoikis.Sig and clinical variables were more discriminative to forecast prognosis than other clinical indicators (Figure 5h). Time‐dependent AUC values verified that Anoikis.Sig and Cox regression model acted better than several clinical indicators in discriminative capability (Figure 5i). Multivariate Cox regression analysis validated that Anoikis.Sig risk score was an independent prognostic predictor in the TCGA‐LUAD cohort (*p* < 0.001) (Figure 5j). DCA curves indicated that predicting prognosis with Anoikis.Sig would provide clinical benefits (Figure 5k), indicating that using Anoikis.Sig to guide clinical decision‐making would result in net clinical benefits from certain interventions, such as more aggressive treatment or intensive monitoring. In summary, those indicators of model assessment comprehensively demonstrated that the Anoikis.Sig showed precision and robustness in model performance.

Figure 5Construction and validation of Anoikis.Sig to predict prognosis in LUAD. (a) A total of 101 kinds of prognostic models via a leave‐one‐out cross‐validation framework and further calculated the C‐index of each model. (b) ROC curves of 1‐, 3‐, and 5‐year OS of Anoikis.Sig in the train cohort. (c) ROC curves of 1‐, 3‐, and 5‐year OS of Anoikis.Sig in the combined test cohort. (d) One‐, 3‐, and 5‐year calibration curves of Anoikis.Sig in the train cohort. (e) One‐, 3‐, and 5‐year calibration curves of Anoikis.Sig in the combined test cohort. (f) Kaplan–Meier survival curves of OS for high‐risk and low‐risk groups in the train cohort. (g) Kaplan–Meier survival curves of OS for high‐risk and low‐risk groups in the combined test cohort. (h) AUC values of 3‐year OS of Anoikis.Sig, the Cox regression model, and clinical variables in the train cohort. (i) Time‐dependent AUC values of Anoikis.Sig, the Cox regression model, and clinical variables in the train cohort. (j) Forest plot visualized the outcome of multivariate Cox regression analysis involving the Anoikis.Sig and clinical variables in the train cohort. (k) DCA curves of Anoikis.Sig, the Cox regression model, and clinical variables in the train cohort.

**Figure:**
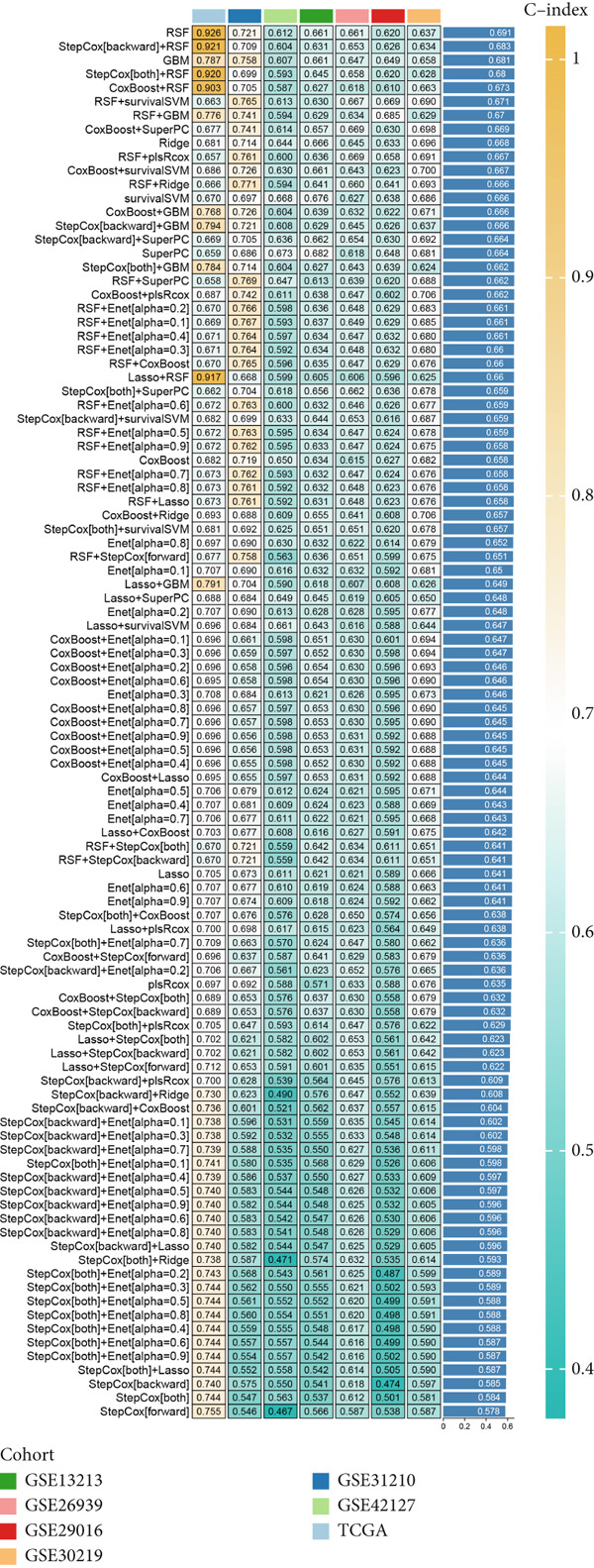
(a)

**Figure:**
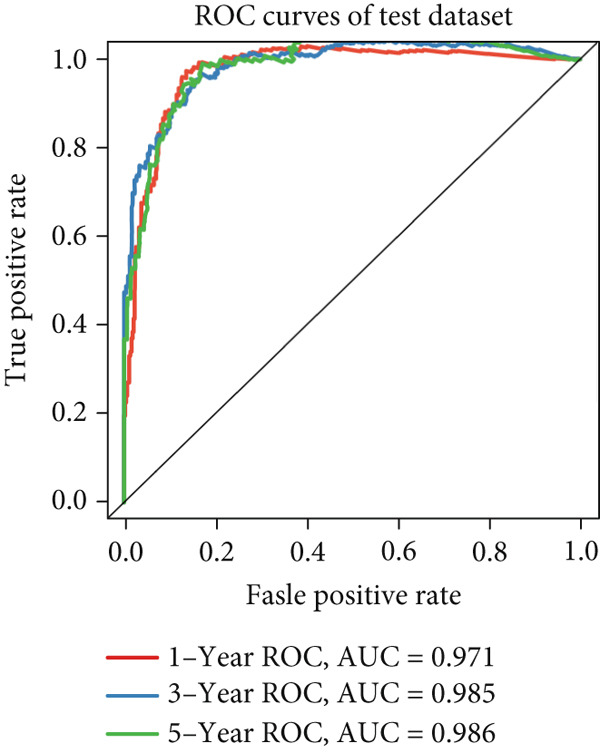
(b)

**Figure:**
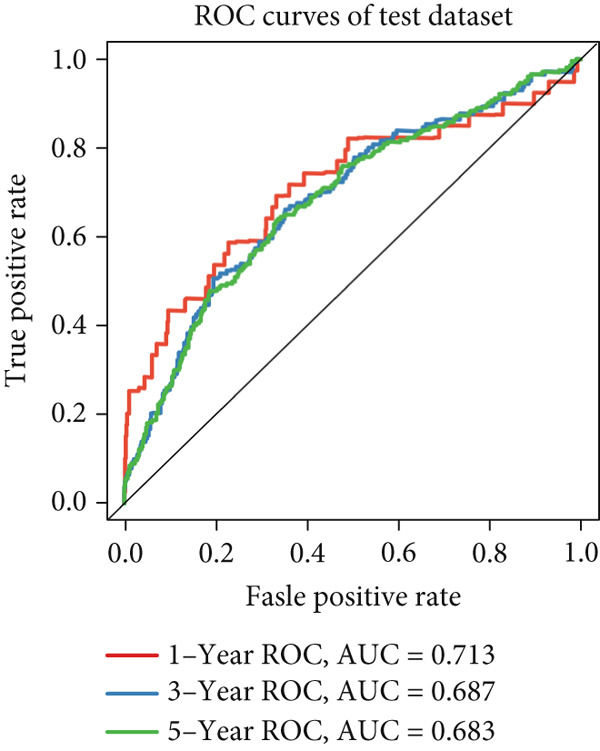
(c)

**Figure:**
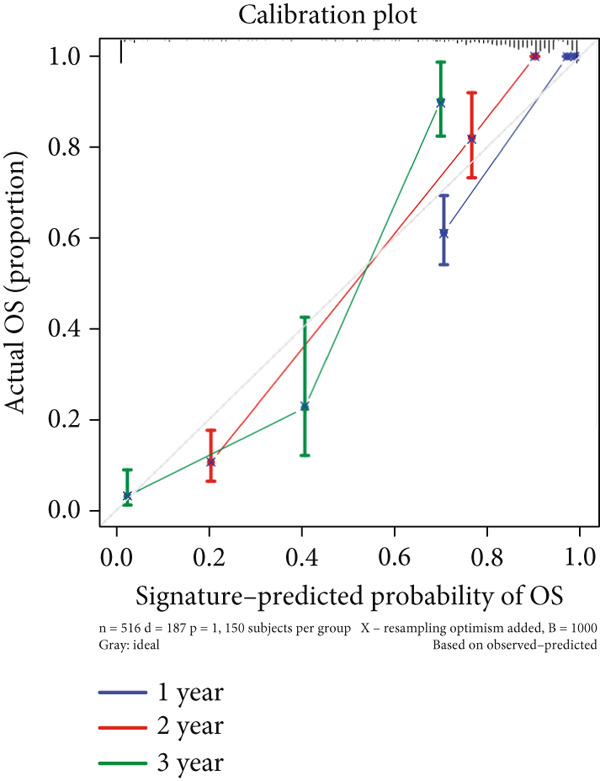
(d)

**Figure:**
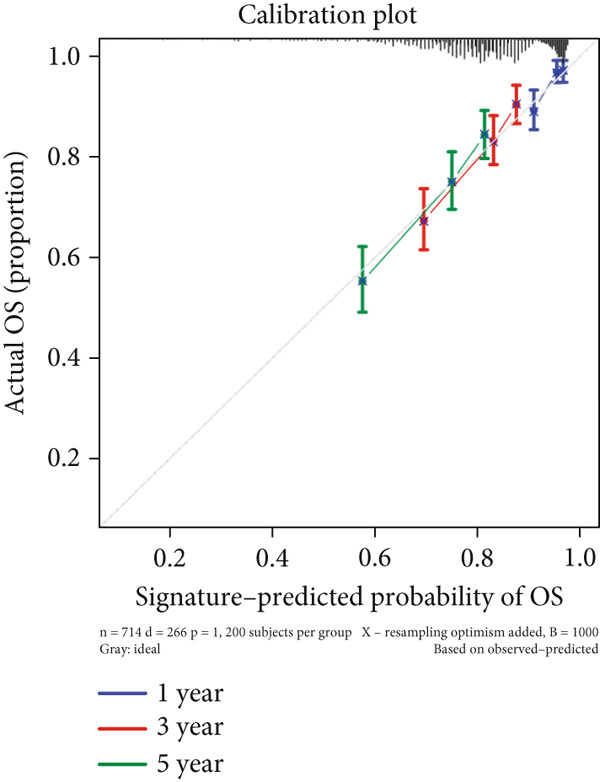
(e)

**Figure:**
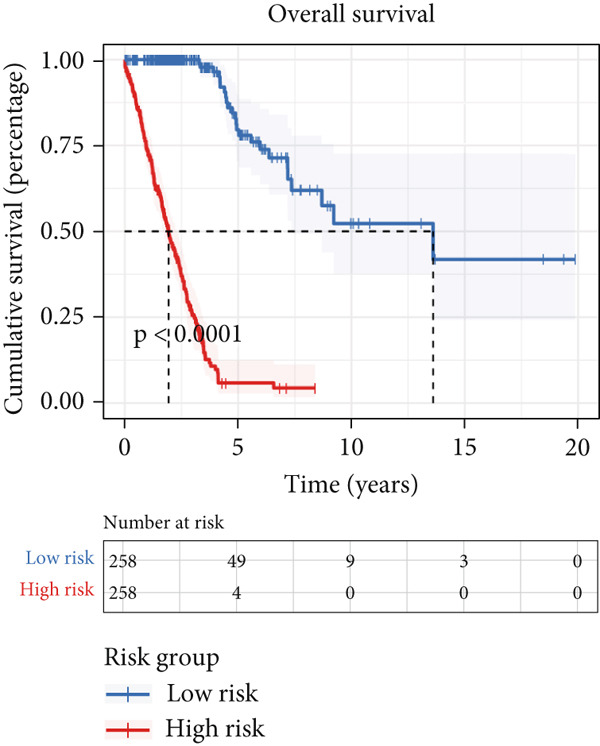
(f)

**Figure:**
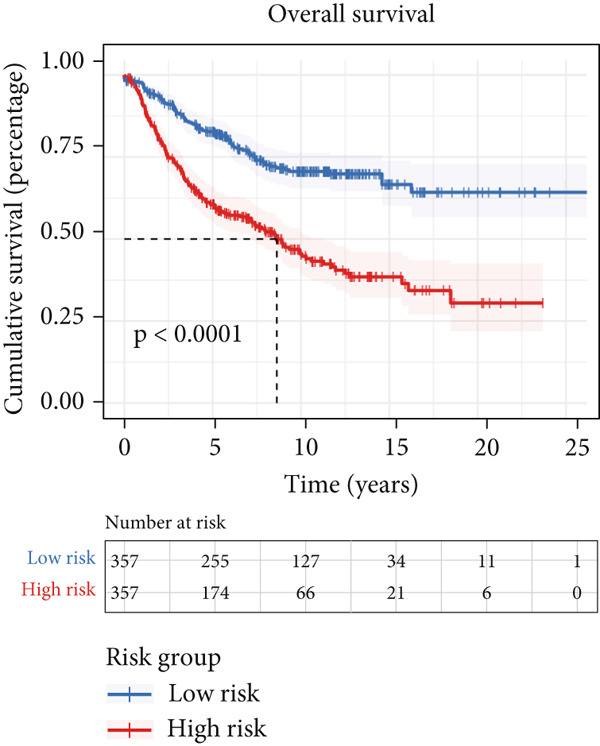
(g)

**Figure:**
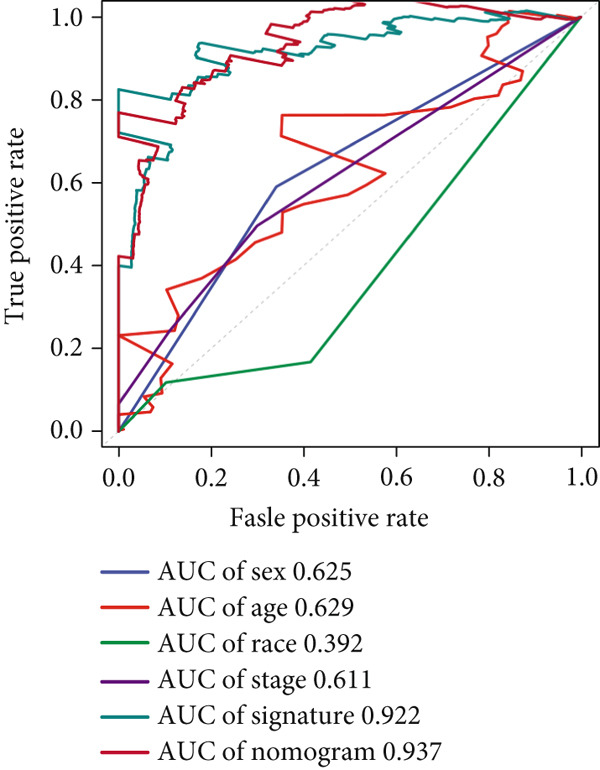
(h)

**Figure:**
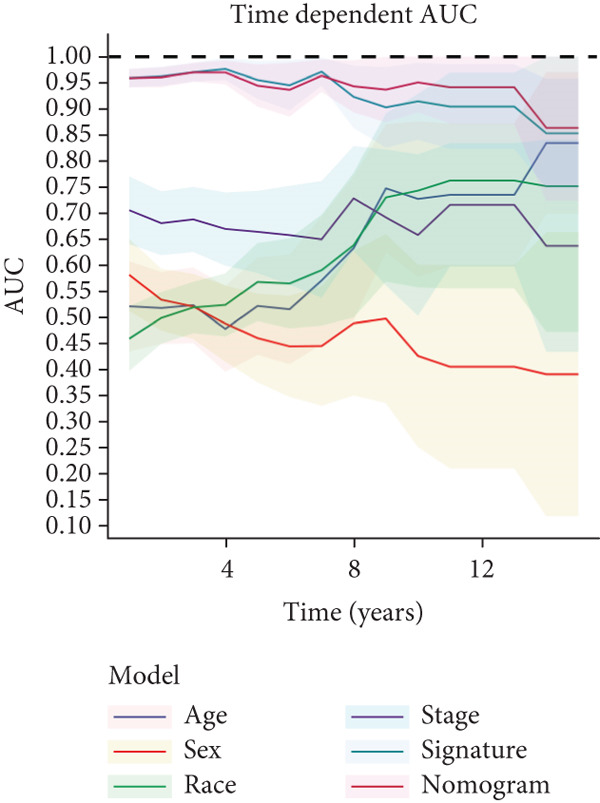
(i)

**Figure:**
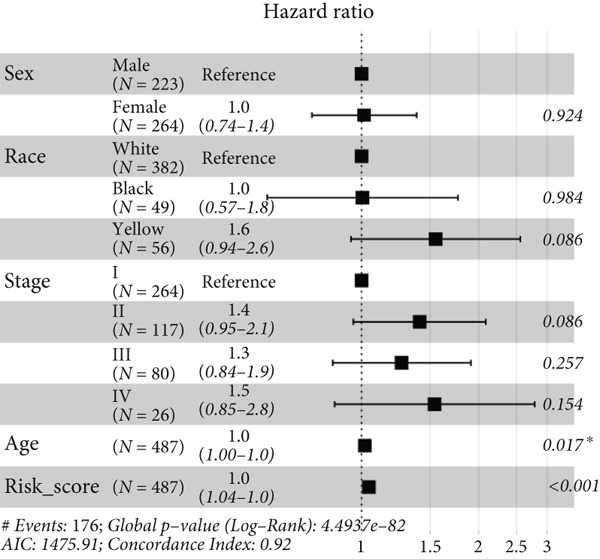
(j)

**Figure:**
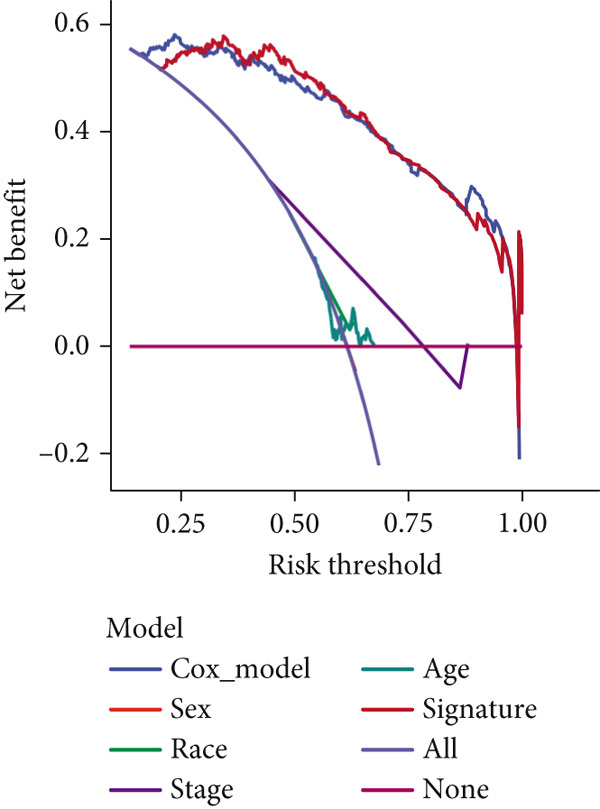
(k)

### 3.6. Model Comparison and Model Evaluation in Immunotherapy Prediction

To test the outperforming prognosis prediction capability of Anoikis.Sig, we collected model gene coefficients of various previously published LUAD prognosis signatures. Subsequently, we analyzed the C‐index of those prognostic models with Anoikis.Sig in LUAD RNA‐seq cohorts. Finally, we demonstrated that the Anoikis.Sig performed better than most of those models in the LUAD datasets in prognosis prediction (Figure 6a), which indicated that Anoikis.Sig acted as a powerful LUAD prognostic model. Then, we illustrated the feature importance of Anoikis.Sig model genes selected by the RSF algorithm, revealing GAPDH as the most impactful factor (Figure 6b). For immunotherapy prediction, we performed survival analysis in three immunotherapy‐related cohorts of LUAD and discovered that patients with high Anoikis.Sig risk scores would suffer worse PFS (Figure 6c) and OS (Figure 6d) than patients with low Anoikis.Sig risk scores. Moreover, responders to immunotherapy would have lower Anoikis.Sig risk scores than nonresponders of immunotherapy, proving the predictive capability of Anoikis.Sig in immunotherapy response (Figure 6e).

Figure 6Model comparisons and model performances in predicting immunotherapy. (a) C‐index comparison analysis between Anoikis.Sig and published signatures in TCGA‐LUAD, GSE31210, GSE42127, GSE13213, GSE26939, GSE29016, GSE30219, and the metacohort.  ^∗^ *p* < 0.05;  ^∗∗^ *p* < 0.01;  ^∗∗∗^ *p* < 0.001;  ^∗∗∗∗^ *p* < 0.0001. (b) The feature selection and feature importance of model variables of Anoikis.Sig in the RSF algorithm. (c) Kaplan–Meier survival curves of PFS for high‐risk and low‐risk groups in three immunotherapy‐treated cohorts. (d) Kaplan–Meier survival curves of OS for high‐risk and low‐risk groups in two immunotherapy‐treated cohorts. (e) Comparison of risk scores calculated by Anoikis.Sig in responders and nonresponders in three immunotherapy‐treated cohorts.

**Figure:**
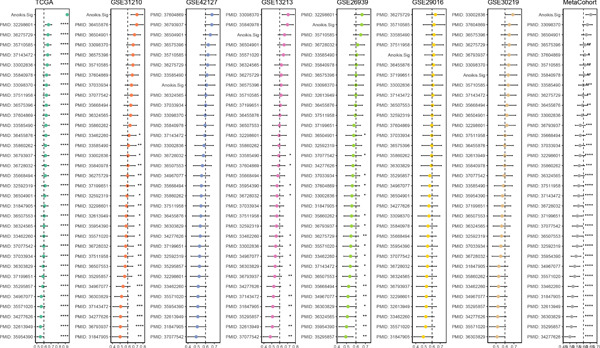
(a)

**Figure:**
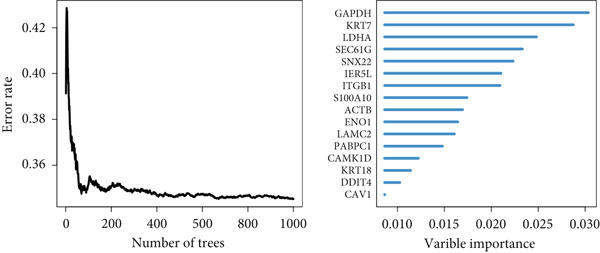
(b)

**Figure:**
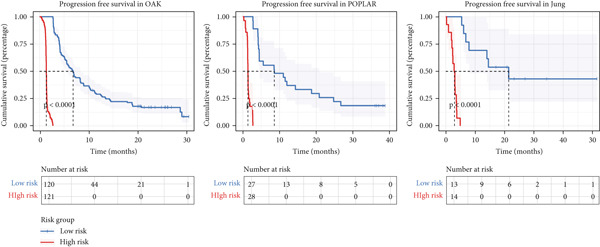
(c)

**Figure:**
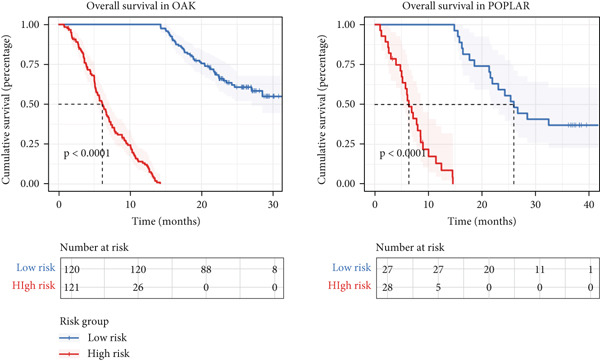
(d)

**Figure:**
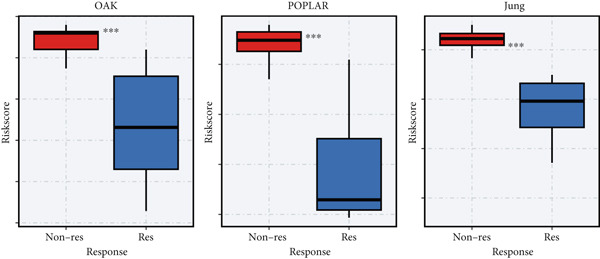
(e)

### 3.7. Function Enrichment and Transcriptome Overview of Model Gene

Further investigation was carried out to decipher the underlying molecular mechanisms of Anoikis.Sig model genes. PCA depicted remarkable disparities between the two risk groups in the TCGA‐LUAD cohort, divided by Anoikis.Sig risk scores (Figure 7a). With DEGs acquired from difference analysis between two risk groups, we conducted functional enrichment analyses via GO and KEGG annotations, which demonstrated that DEGs were enriched in mitotic spindle elongation in GO annotation and proteasome in KEGG annotation (Figure 7b,c). GSEA decoded that cellular senescence was upregulated in high‐risk patients, and the cAMP signaling pathway was inhibited in high‐risk patients (Figure 7d,e). Moreover, GSVA based on Hallmark gene set indicated that high‐risk patients were abundant in glycolysis and decreased in KRAS signaling (Figure 7f). The distinct expression profiles of Anoikis.Sig model genes and the disparities of clinic indicators between the two risk groups were noteworthy (Figure 7g). And Spearman correlation analysis displayed the significant correlations (correlation *p* value < 0.001) among Anoikis.Sig model genes (Figure 7h). The above analysis results revealed the diverse transcriptome overviews in high‐risk and low‐risk patients, respectively.

Figure 7Functional enrichment analysis and transcriptome landscape of model genes. (a) PCA analysis plot of the high‐risk group and the low‐risk group. (b, c) GO and KEGG enrichment analyses of DEGs among the two risk groups. (d–f) GSEA and GSVA analyses of DEGs among the two risk groups. (g) Differences in the expression of model genes and differences in the clinical variables of LUAD patients among the two risk groups. (h) The molecular interaction network plot visualized the correlations among the expressions of model genes and their prognostic prediction value. Significantly positive and negative correlations are shown as red and blue lines, respectively. The color and size of the nodes indicate the type of model genes and *p* values from Cox regression.

**Figure:**
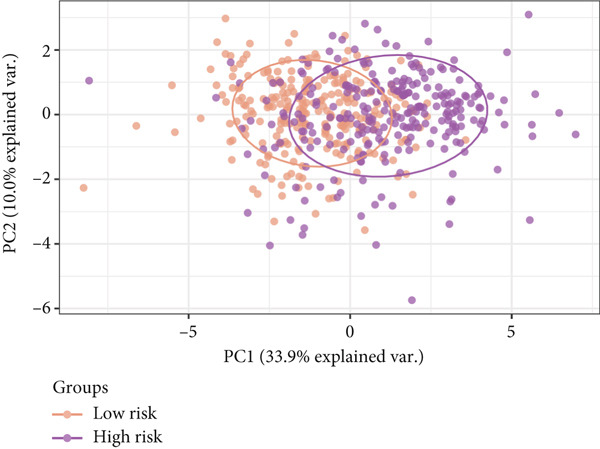
(a)

**Figure:**
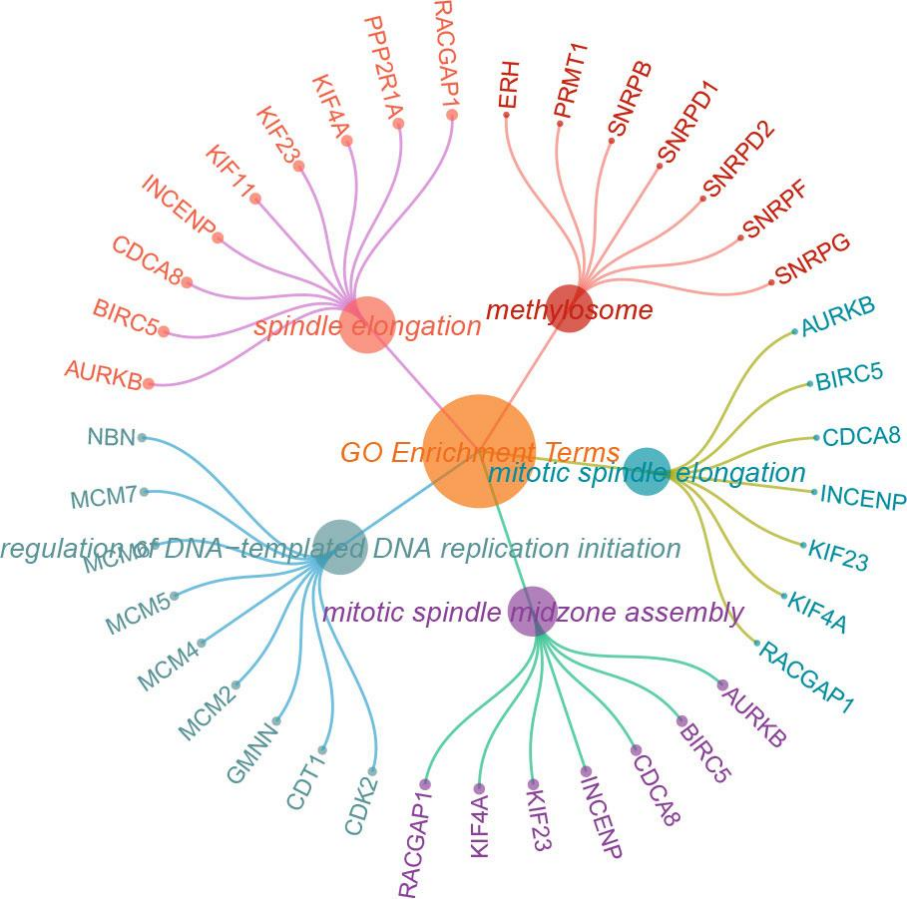
(b)

**Figure:**
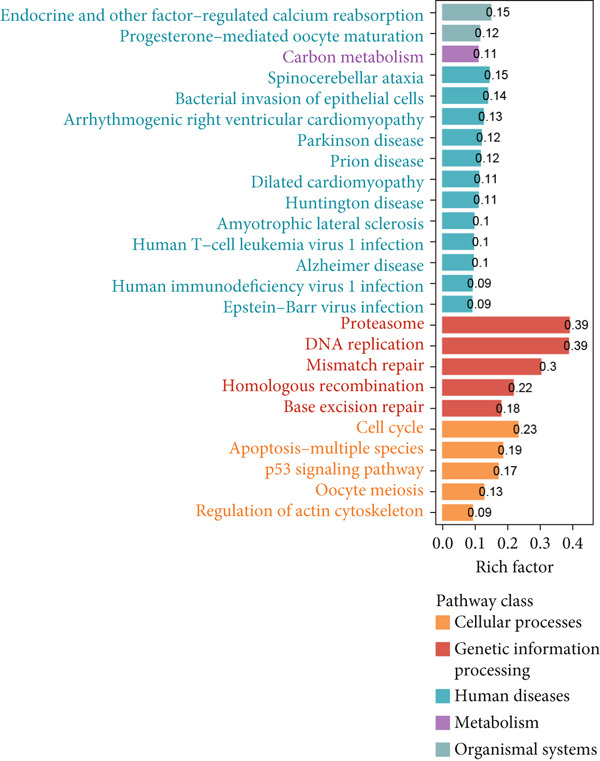
(c)

**Figure:**
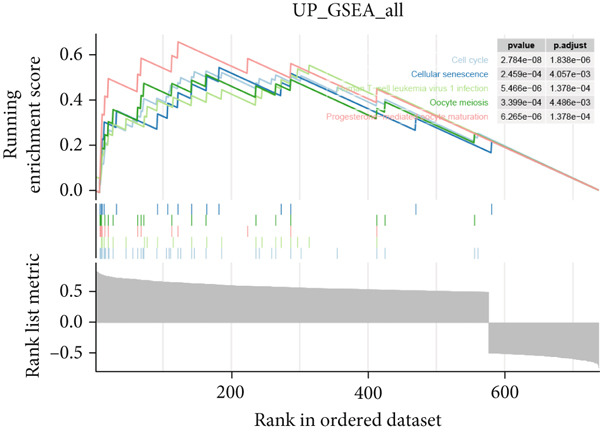
(d)

**Figure:**
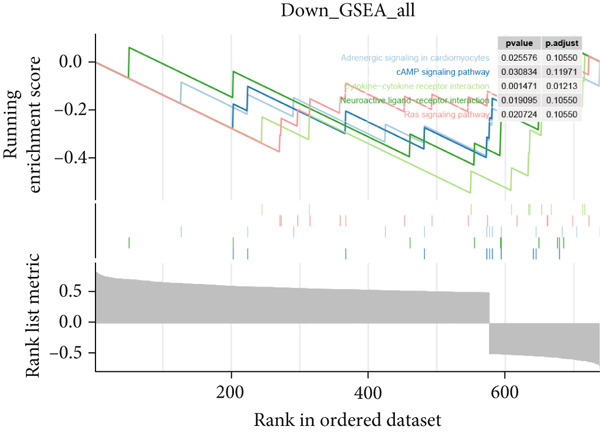
(e)

**Figure:**
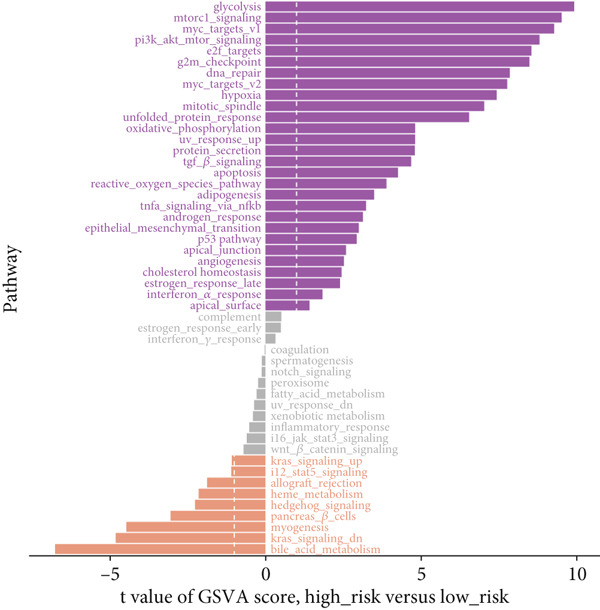
(f)

**Figure:**
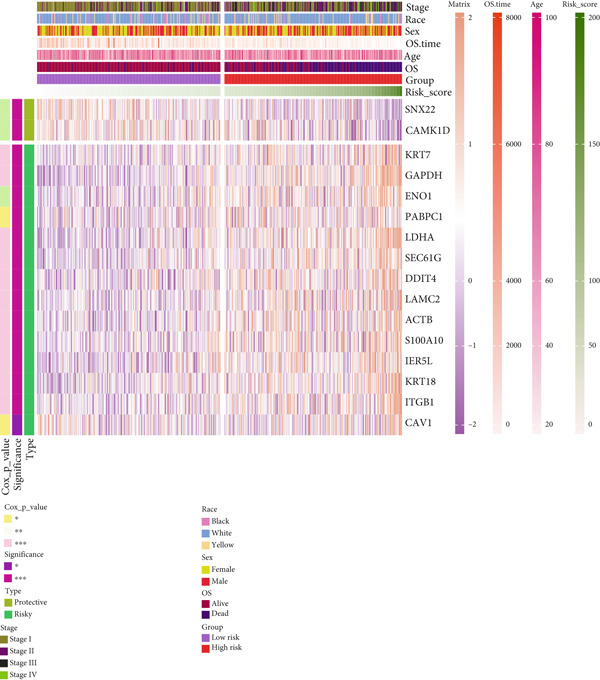
(g)

**Figure:**
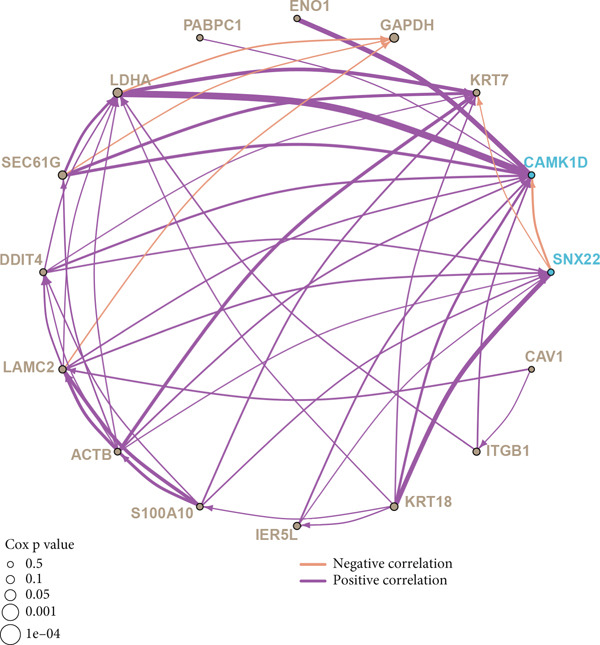
(h)

### 3.8. Applications of Anoikis.Sig in Immune Microenvironment

To investigate the discriminative capability of Anoikis.Sig in the immune microenvironment, we computed the immune cell abundances in two risk groups in the TCGA‐LUAD cohort by eight immune algorithms. We sought to demonstrate the remarkably worse immune cell infiltrations in high‐risk cases (Figure 8a). Besides, we performed Spearman correlation analysis to reveal close relations among immune cell abundances, Anoikis.Sig risk scores, and Anoikis.Sig model gene expressions (Figure 8b). Interestingly, we found that low‐risk patients owned higher immune checkpoint gene expressions, reminding a possibility of responding to immunotherapy (Figure 8c). Furthermore, we conducted ssGSEA analysis with immune function‐related signatures, which depicted that low‐risk patients were remarkably more abundant in the immune microenvironment (Figure 8d). Besides, ssGSEA analysis of gene signatures of six key steps in the cancer immunity cycle displayed that low‐risk patients were more responsive to cancer treatments (Figure 8e). The Spearman correlation method decoded the inverse relations between immune function levels and Anoikis.Sig risk scores (Figure 9a). All of the aforementioned analysis results demonstrated the well discrimination of Anoikis.Sig in analyzing TME of LUAD.

Figure 8Comprehensive analysis of the TME in different risk groups in TCGA‐LUAD cohort. (a) Differences in immune infiltration status between the two risk groups were evaluated by eight immune algorithms. (b) Heatmap visualized the correlation between different immune cells and risk scores and the relationship between different immune cells and expressions of model genes. (c) The differences in the expressions of immune checkpoint–related genes between the two risk groups. (d) The differences in immune function scores calculated by ssGSEA analysis between the two risk groups. (e) The differences in cancer immunity cycle scores based on ssGSEA analysis between the two risk groups.

**Figure:**
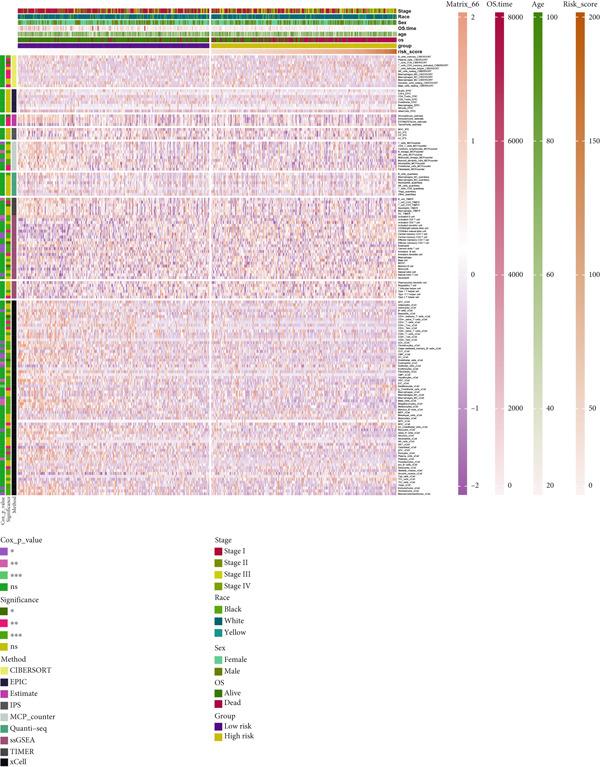
(a)

**Figure:**
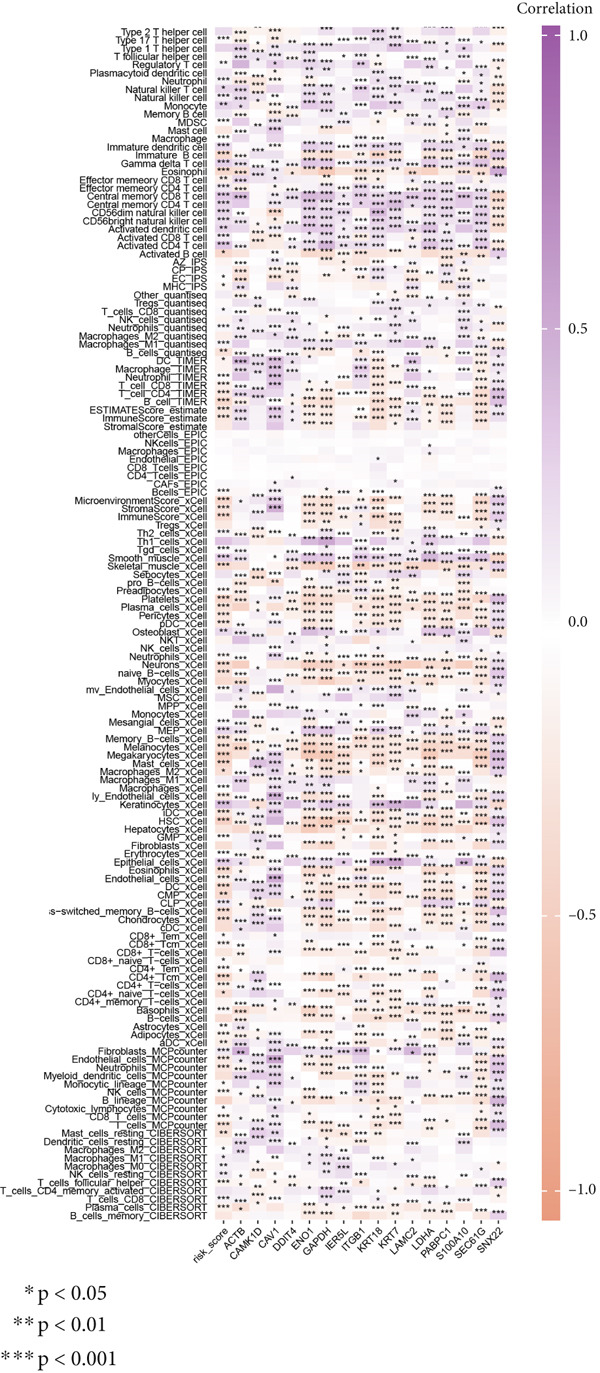
(b)

**Figure:**
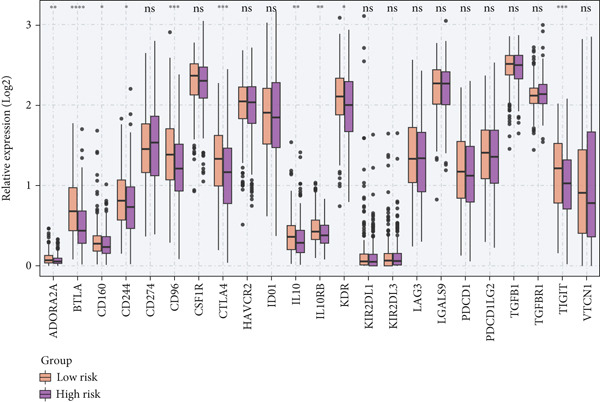
(c)

**Figure:**
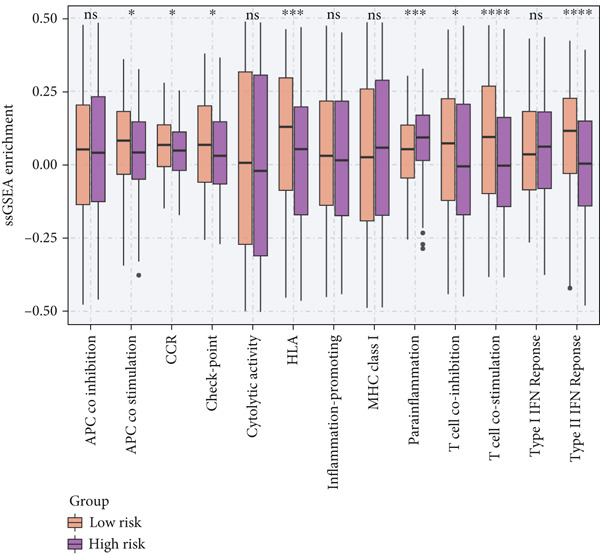
(d)

**Figure:**
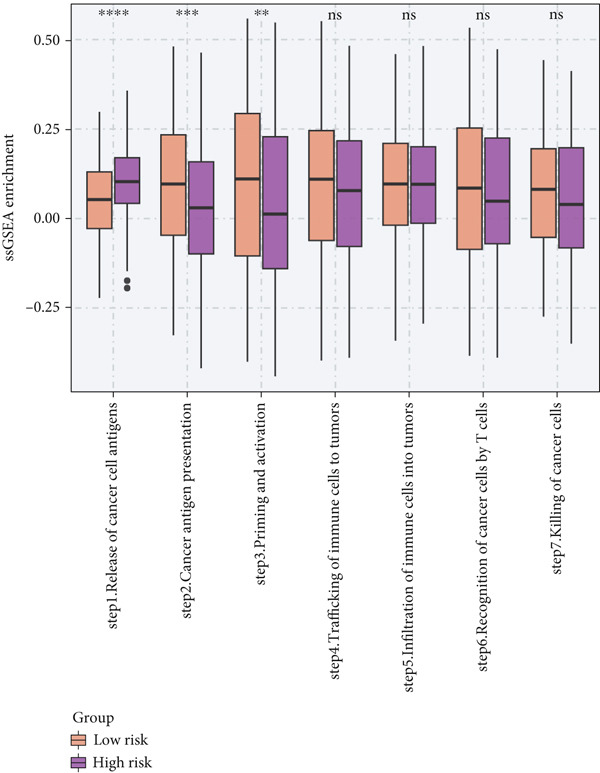
(e)

Figure 9General landscapes of immune, somatic mutation, and CNVs in high‐risk and low‐risk groups. (a) The correlations between risk scores and immune function scores in a bubble plot. (b) The correlations between the risk scores, immune functions, and metabolic‐related pathways based on GSVA analysis of KEGG terms were displayed in a butterfly plot. (c) The molecular interaction network plot visualized the correlations among immune cells and fibroblasts in TME and their prognostic prediction value. (d) The mutational landscapes of model genes in the TCGA‐LUAD cohort. (e, f) Visual summary displayed common genetic alterations in the low‐risk and high‐risk groups in the TCGA‐LUAD cohort. (g, h) Variant classification, variant type, SNV class, variants per sample, variant classification summary, and the Top 10 mutated genes in the low‐risk and high‐risk groups in the TCGA‐LUAD cohort. (i) Tumor mutation burdens between high‐risk and low‐risk groups in the TCGA‐LUAD cohort. (j) Spearman correlation between risk scores and TMB scores in the TCGA‐LUAD cohort. (k) Comprehensive survival analysis on OS based on two risk groups and two TMB groups in the TCGA‐LUAD cohort.

**Figure:**
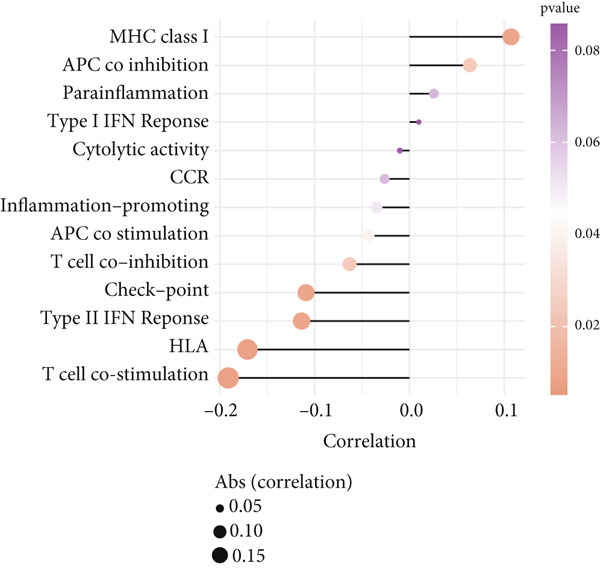
(a)

**Figure:**
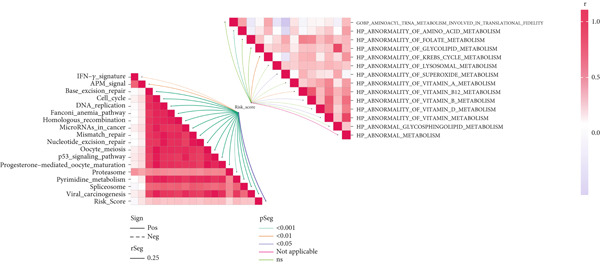
(b)

**Figure:**
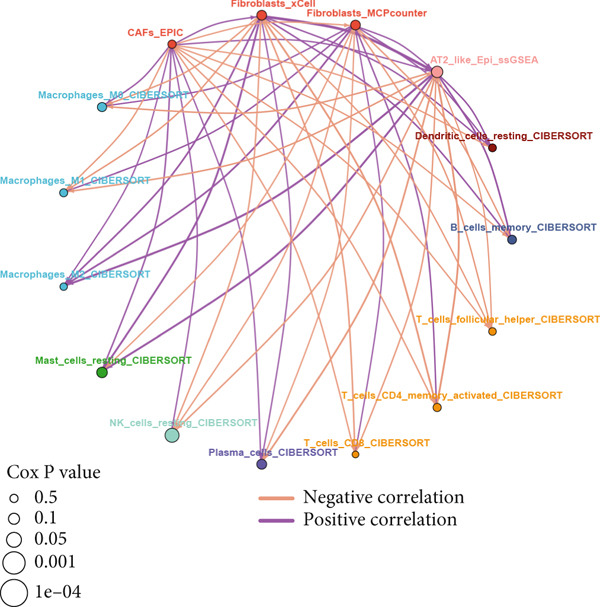
(c)

**Figure:**
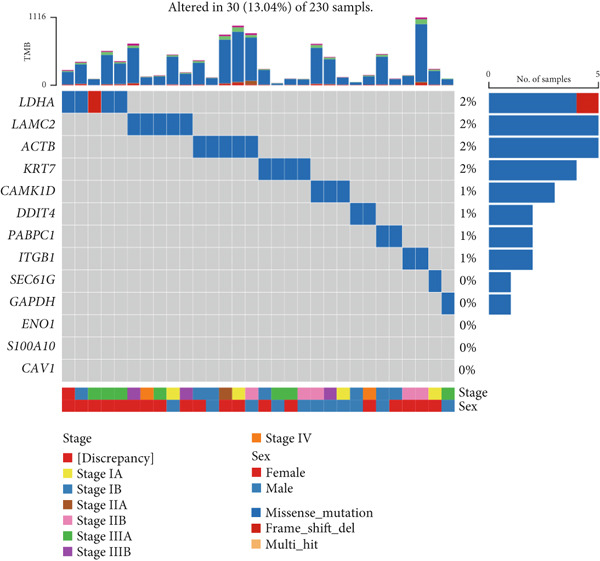
(d)

**Figure:**
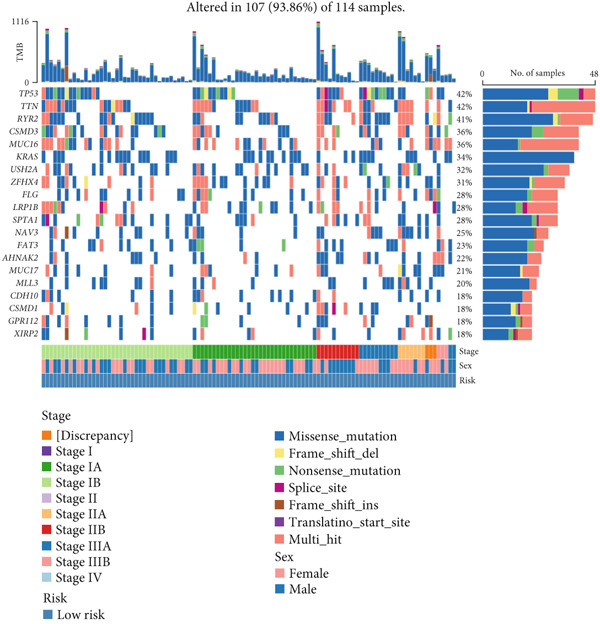
(e)

**Figure:**
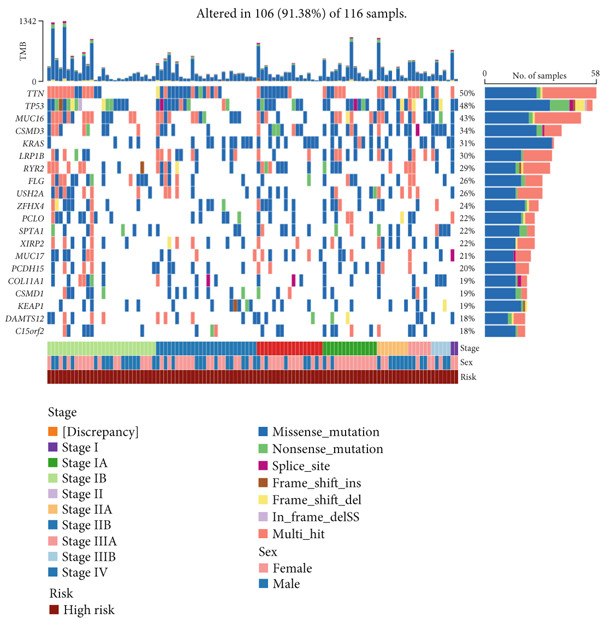
(f)

**Figure:**
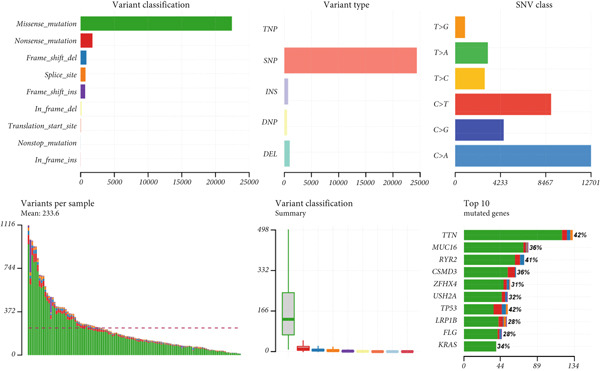
(g)

**Figure:**
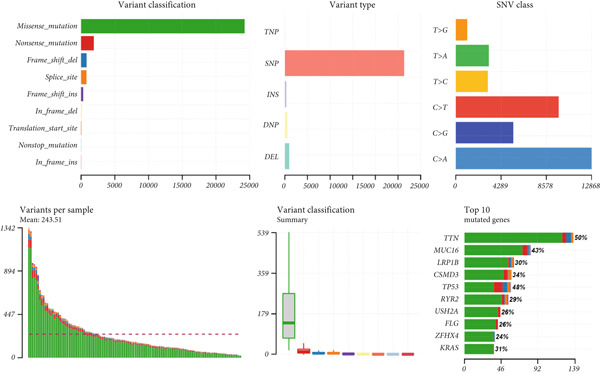
(h)

**Figure:**
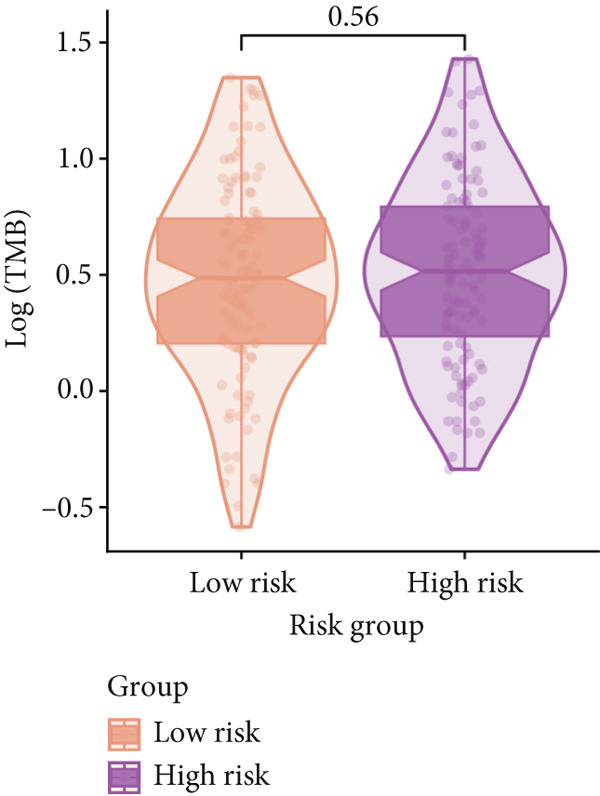
(i)

**Figure:**
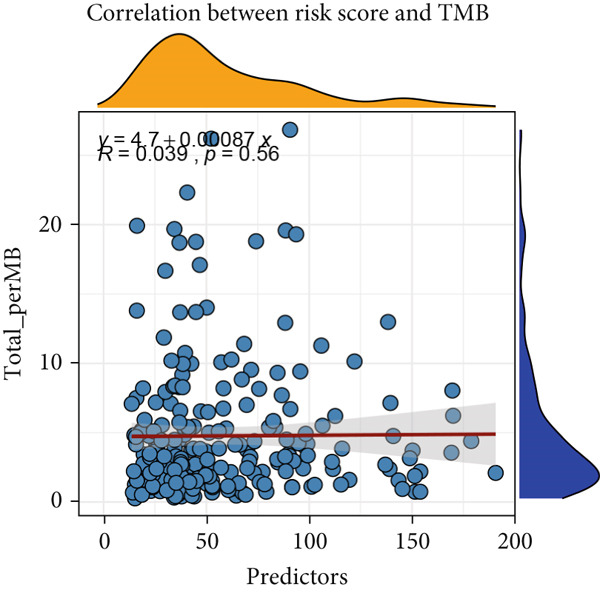
(j)

**Figure:**
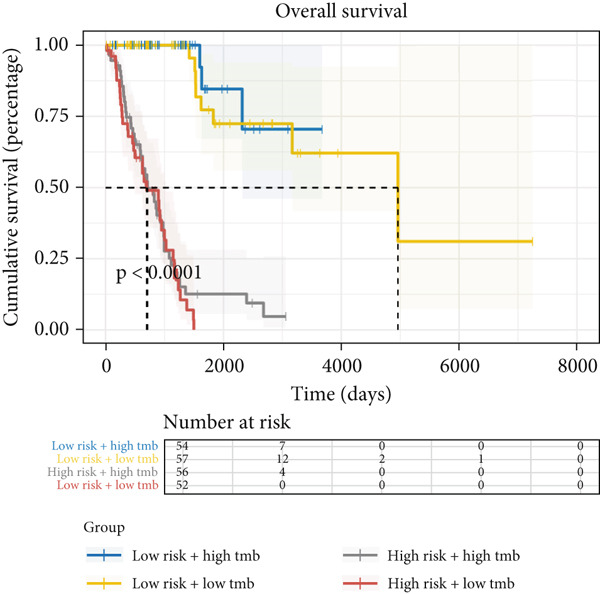
(k)

### 3.9. Overall Outline of Immune Infiltration and Gene Mutational Profiles

Activities of immune‐related pathways were important in cancer immunity, so we analyzed these immune function levels using ssGSEA analysis based on corresponding gene signatures and revealed their close relations to Anoikis.Sig risk scores (Figure 9a). Given the well‐known distinct metabolic reprogramming of cancer cells [41], we sourced metabolic pathway signatures from the KEGG database to investigate the relationship among Anoikis.Sig risk scores and metabolic pathway activities, which showed intricate metabolic implications of Anoikis.Sig (Figure 9b). With immune cell abundances computed by eight immune algorithms, we next conducted Spearman correlation analysis to investigate the obvious correlations (correlation *p* value < 0.001) among immune cells and fibroblasts in TME, as well as their prognosis prediction value (Figure 9c). Subsequently, we sought to conduct multiomics analyses to reveal the genetic landscapes of two risk groups in the TCGA‐LUAD cohort, as well as the mutational landscapes of Anoikis.Sig model genes (Figure 9d). We successfully portrayed the overview of somatic mutations in low‐risk group (Figure 9e) and high‐risk group (Figure 9f) with clinical indicators in TCGA‐LUAD cohort, as well as variant classification, variant type, SNV class, variants per sample, variant classification summary, and Top 10 mutated genes in low‐risk cases (Figure 9g) and high‐risk cases (Figure 9h). And we discover that high‐risk cases had more TMB than low‐risk cases without significance (Figure 9i), while TMB was positively correlated to Anoikis.Sig risk scores according to Spearman correlation analysis without significance (Figure 9j). Moreover, we categorized the TCGA‐LUAD cohort into four subgroups based on median TMB and median risk score. Survival analysis showed that high‐risk patients with low TMB owned the worst OS and low‐risk patients with high TMB showed the best OS, with significance (*p* < 0.0001, Figure 9k).

### 3.10. Implication of Immunotherapy and Potential Therapeutic Targets

Leveraging the TIDE website and submap analysis, the response to immunotherapy of LUAD patients was assessed between two risk groups. In the TCGA‐LUAD cohort, high‐risk cases owned remarkably higher TIDE, TIDE dysfunction, and TIDE exclusion scores, which indicated probabilities to suffer immune escape and immune exhaustion during immunotherapy (Figure 10a). In the IMvigor210 cohort, low‐risk cases who are reactive to immunotherapy owned the best OS, whereas high‐risk cases who are not reactive to immunotherapy exhibited the worst OS (Figure 10b). Moreover, there were significantly larger proportions of responders in low‐risk patients (Figure 10c). Therefore, the submap algorithm was utilized to demonstrate that low‐risk cases were more reactive to PD1 inhibitors, indicating the capability of Anoikis.Sig to select the qualified responders of immunotherapy (Figure 10d). Subsequently, we contrasted the responsiveness of pancancer patients in immunotherapy cohorts between two risk groups, which demonstrated that patients reactive to immunotherapy had significantly lower risk scores (Figure 10e). Finally, to reveal novel chemotherapy drugs for high‐risk cases selected by Anoikis.Sig, we predicted the drug reaction based on drug sensitivity information sourced from CTRP and PRISM databases. By cross‐correlating these pharmacogenomics datasets, we jubilantly acquired seven promising medicines or compounds, which exhibited therapeutic efficacy in high‐risk LUAD cases (Figure 10f).

Figure 10Immunotherapy information and chemotherapy sensitivity in two risk groups. (a) The violin diagram illustrated the significant variance in TIDE scores between high‐risk and low‐risk groups in the TCGA‐LUAD cohort. (b) Kaplan–Meier survival analysis delineated the OS rates for patients categorized into high‐risk and low‐risk groups in the IMvigor cohort. (c) The TIDE algorithm predicted response to immunotherapy between high‐risk and low‐risk groups in the TCGA‐LUAD cohort. (d) Comprehensive submap analysis predicted response to immunotherapy between high‐risk and low‐risk groups in the TCGA‐LUAD cohort. (e) The box diagram depicted the disparity in risk scores among immunotherapy patients in the IMvigor210, GSE78220, GSE135222, and GSE91061 immunotherapy cohorts. (f) Correlation study and differential drug response analysis of CTRP‐derived pharmaceuticals and PRISM‐derived pharmaceuticals to explore potential drugs for high‐risk patients in the TCGA‐LUAD cohort.

**Figure:**
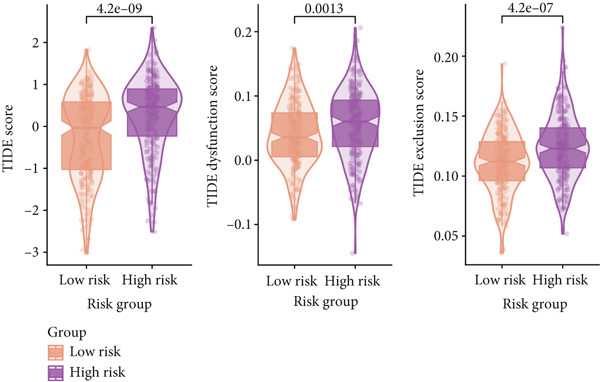
(a)

**Figure:**
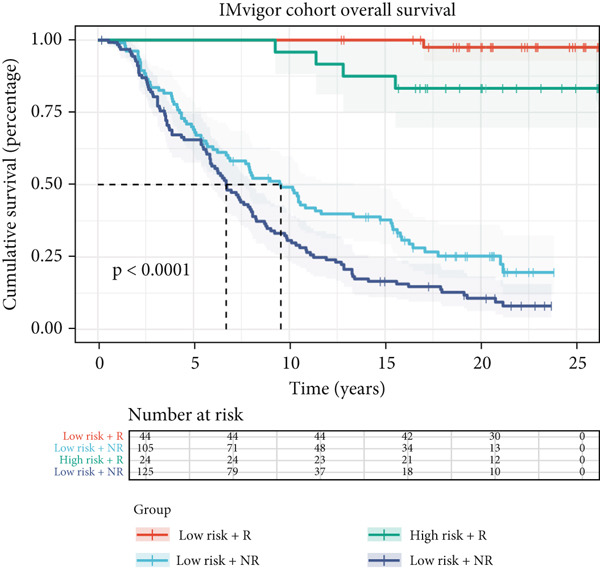
(b)

**Figure:**
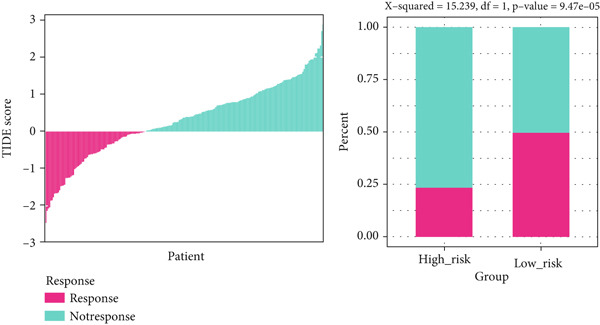
(c)

**Figure:**
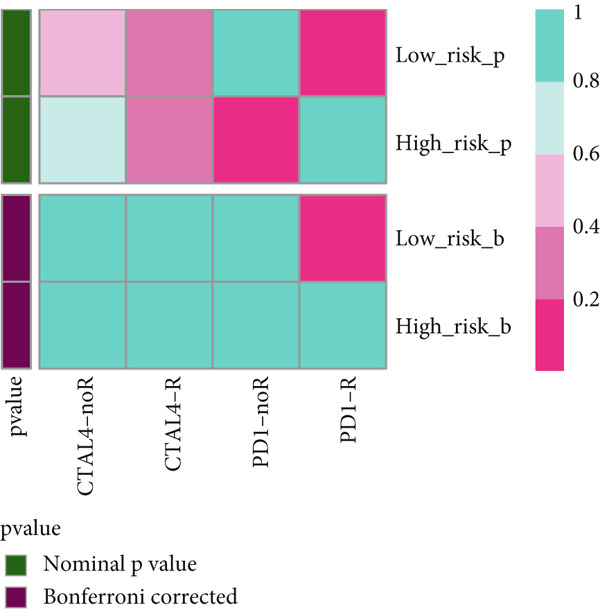
(d)

**Figure:**
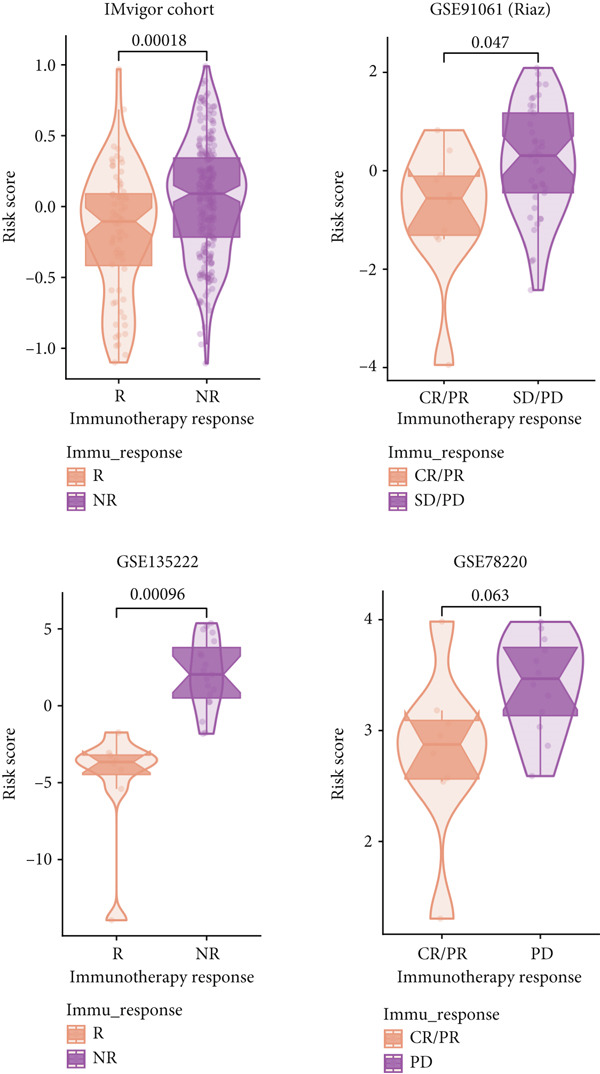
(e)

**Figure:**
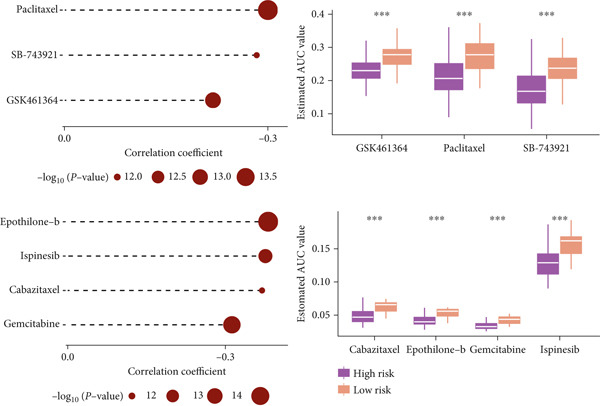
(f)

### 3.11. Identification of Anoikis.Sig‐Related Clusters

To deeply investigate the expression profiles of Anoikis.Sig model genes, three LUAD RNA‐seq cohorts were chosen to conduct consensus molecular clustering analysis. We harnessed Anoikis.Sig model genes to carry out unsupervised clustering analysis in LUAD cohorts, revealing *k* = 2 with outperforming discriminative ability (Figure 11a). Survival analysis proved their distinct survival outcomes, with Cluster 2 having significantly worse OS (Figure 11b). Moreover, PCA (Figure 11c) and t‐SNE (Figure 11d) dimensional reduction analysis revealed oblivious disparities between the two clusters. Analyzing DEGs of Cluster 2 compared to Cluster 1, we conducted functional enrichment analysis to reveal DEG enrichment in intermediate filament‐based processes in GO annotation and ECM–receptor interaction in KEGG annotation (Figure 11e,f). GSEA analysis manifested that keratinocyte differentiation was abundant in Cluster 2 and adaptive immune response was inhibited in Cluster 2 (Figure 11g). GSVA analysis based on Hallmark gene set revealed that Cluster 2 was enriched in TGF‐beta signaling and inhibited in DNA repair (Figure 11h). Hence, the transcriptome landscapes of Anoikis.Sig model genes and the clinical indicators between the two clusters were pointed out with significant differences (Figure 11i). Next, we harnessed immune infiltration algorithms to assess the immune infiltration disparities between two clusters, as well as decoding the prognosis value of each cell subtype (Figure 11j).

Figure 11Consensus clustering analysis of model gene‐related clusters in three LUAD cohorts (TCGA‐LUAD, GSE31210, and GSE42127). (a) Consensus matrices of LUAD patients in three cohorts for *k* = 2. (b) Kaplan–Meier survival analysis of OS between two clusters. (c, d) PCA and t‐SNE analysis of two clusters. (e, f) GO and KEGG enrichment analysis indicated significant enrichment of pathways in Cluster 2. (g, h) GSEA and GSVA analyses of DEGs among the two clusters. (i) ComplexHeatmap of the distribution and expression of model genes and clinical variables in the two clusters in the TCGA‐LUAD cohort. (j) Differences in the proportion of various kinds of immune cells calculated by eight immune algorithms in the two clusters in the TCGA‐LUAD cohort.

**Figure:**
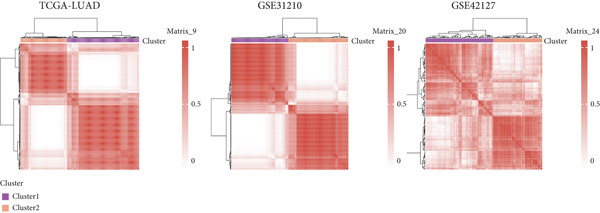
(a)

**Figure:**
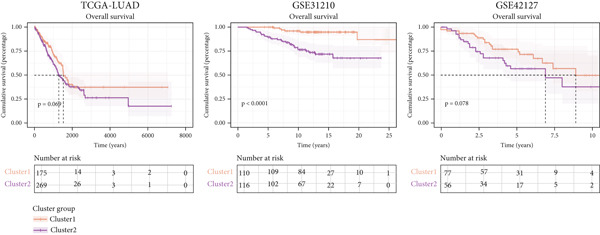
(b)

**Figure:**
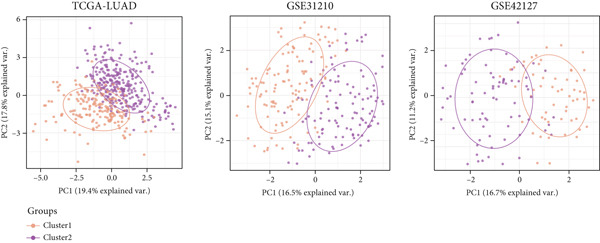
(c)

**Figure:**
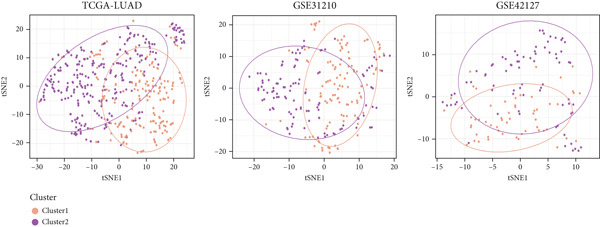
(d)

**Figure:**
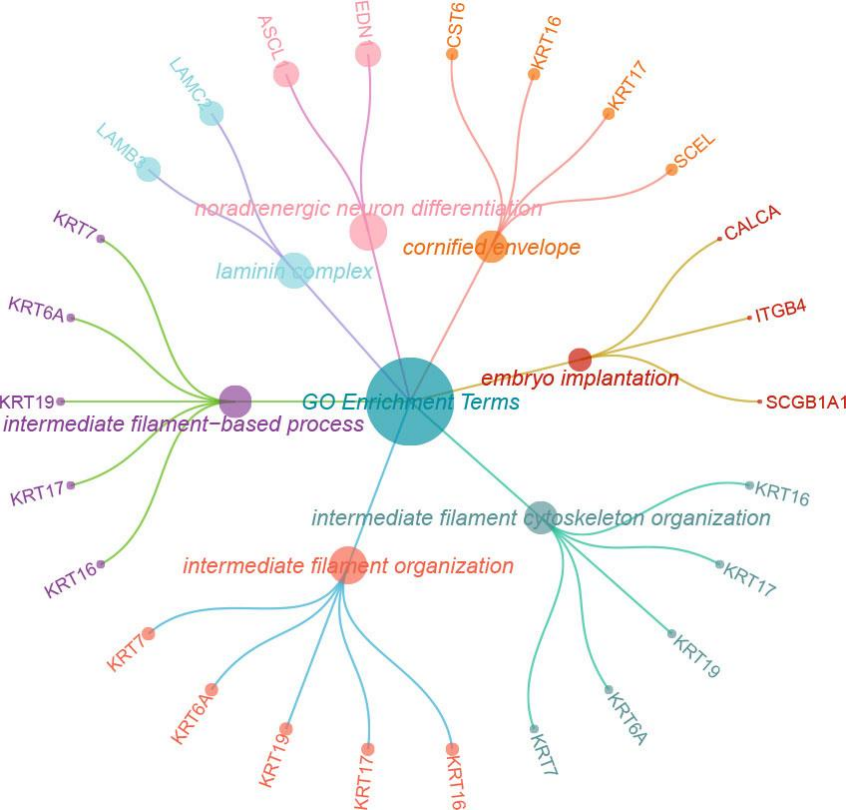
(e)

**Figure:**
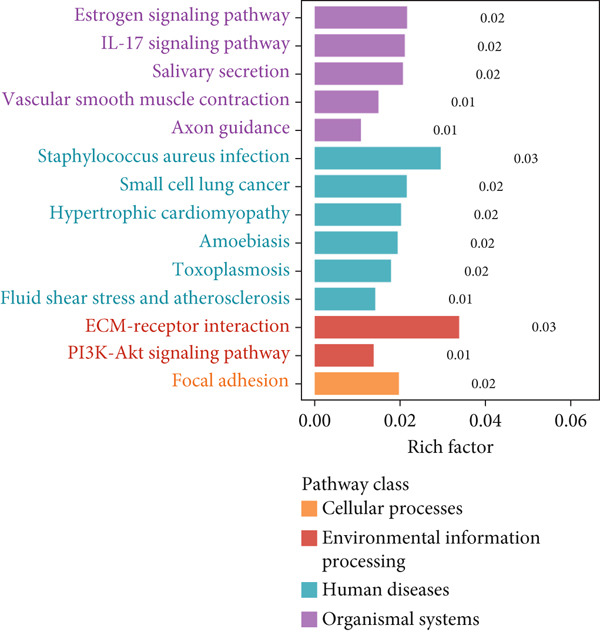
(f)

**Figure:**
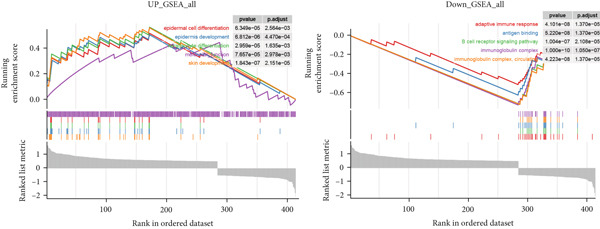
(g)

**Figure:**
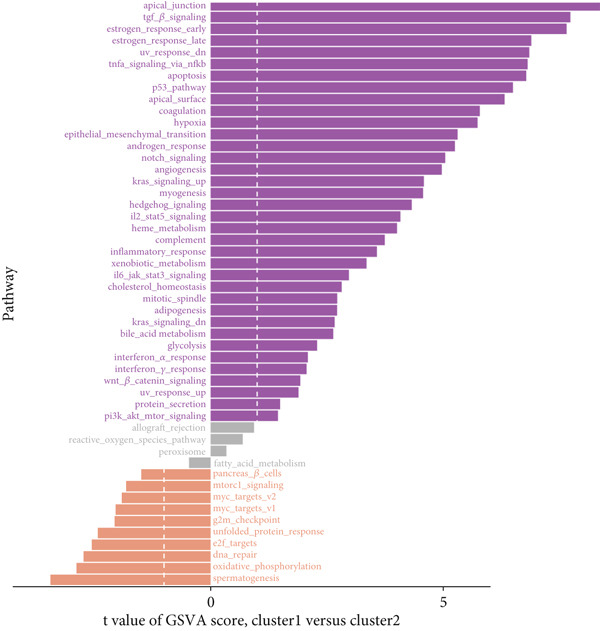
(h)

**Figure:**
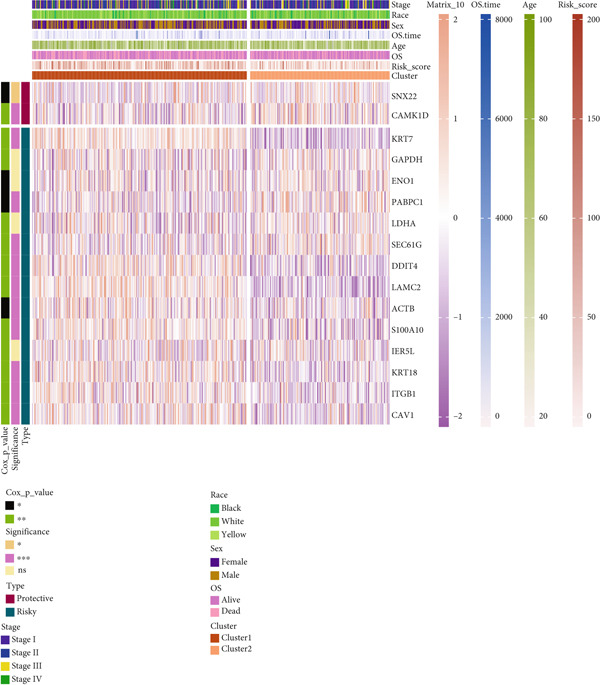
(i)

**Figure:**
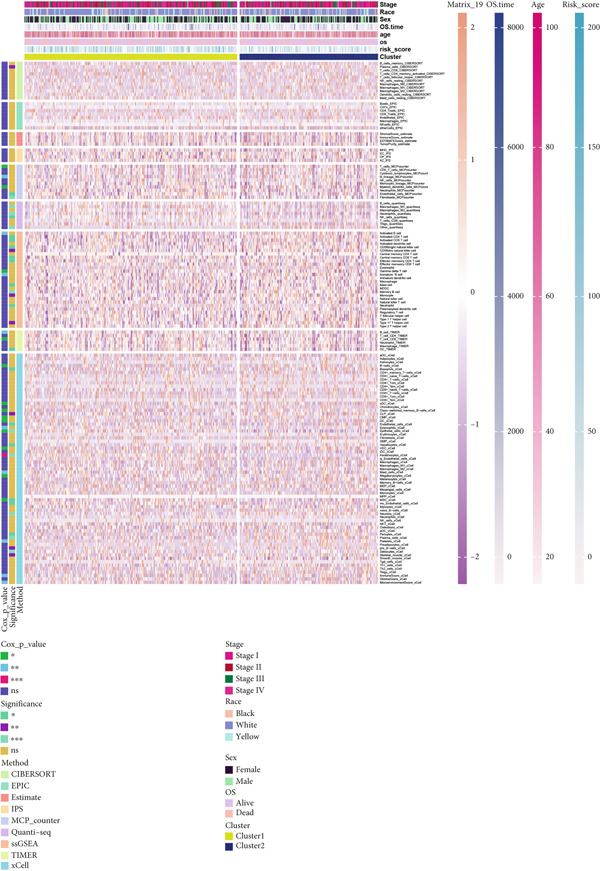
(j)

### 3.12. Genetic Profiles of Model Genes, Immune Subtypes, and Clinical Information

With CNV information in the TCGA‐LUAD cohort, we depicted the loci of mutations of Anoikis.Sig model genes on the chromosome (Figure 12a), while we illustrated that SNX22 owned the most somatic CNV frequency of loss in model genes and S100A10 owned the most somatic CNV frequency of gain in model genes (Figure 12b). Meanwhile, we successfully defined five immunological subtypes of TCGA‐LUAD patients, while we demonstrated significant disparities of respective proportions of immunological subtypes between risk groups and clusters, reminding less inflammatory (C3) pattern in high‐risk cases and Cluster 2 (Figure 12c). Besides, we visualized the diverse constitution of TCGA‐LUAD patients among risk groups, clusters, and clinical information through a “Sankey plot” (Figure 12d). After all, comparing clinical data between two risk groups indicated that low‐risk cases owned better prognosis and better clinical status in the TCGA‐LUAD cohort (Figure 12e).

Figure 12Comprehensive landscapes of model genes, as well as two risk groups and two clusters divided by them. (a) Chromosome position and alteration of all model genes. (b) The CNV mutation frequency of the prognostic model genes. (c) Differences in the proportion of five immune subtypes between two clusters and two risk groups. (d) Sankey diagram illustrating the distributions in two clusters and two risk groups with different clinical variables and survival outcomes. (e) A circular pie chart visualized the proportion difference of clinical indices and immune subtypes between the two risk groups in the TCGA‐LUAD cohort.

**Figure:**
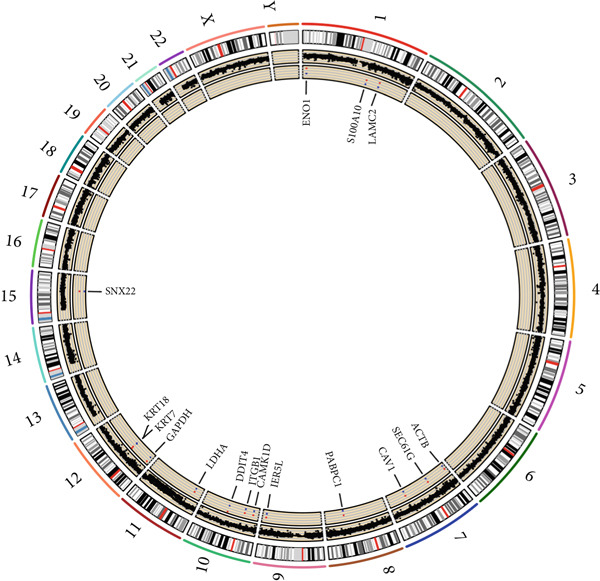
(a)

**Figure:**
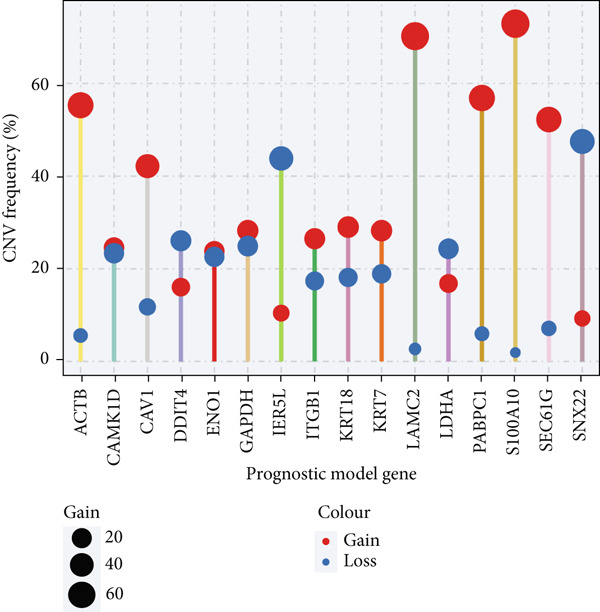
(b)

**Figure:**
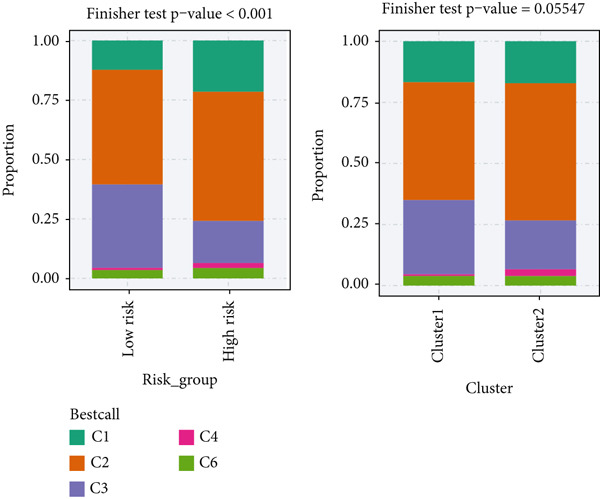
(c)

**Figure:**
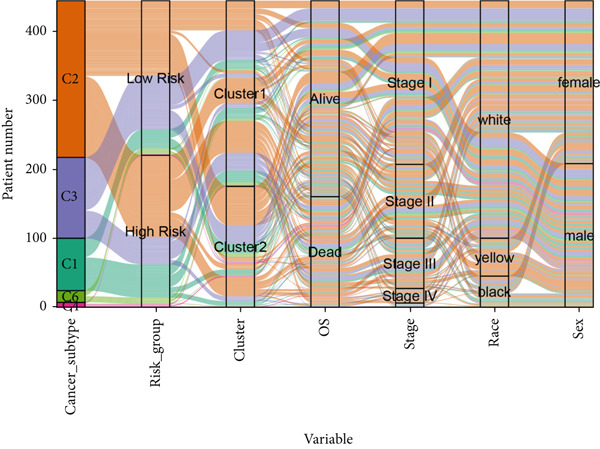
(d)

**Figure:**
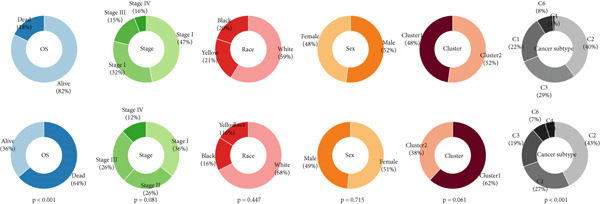
(e)

### 3.13. Cell Scoring, Scissor Algorithm, and Pseudotime Analysis

To verify the risk‐stratify capability of Anoikis.Sig in the scRNA‐seq viewpoint, we computed the Anoikis.Sig enrichment risk scores via scRNA‐seq scoring algorithms, which demonstrated that cells with higher risk scores were mostly ample in AT2‐like Epi cells (Figures 13a, 13b, 13c, and 13d). To decode the cell origins manifesting high‐risk clinical manifestations, we conducted the “Scissors” analysis to search for relations between RNA‐seq data and scRNA‐seq data, which sifted out cells with obvious alignments to the clinical phenotypes. We labeled high‐risk and low‐risk status of patients as our prime phenotypes in RNA‐seq data, thus supporting the comprehensive search of high‐risk cells (Scissors+ cells) and low‐risk cells (Scissors− cells) (Figure 13e). The Anoikis.Sig risk scores of Scissors+ cells, computed by four scRNA‐seq scoring algorithms, were remarkably higher than Scissors− cells and various cell subtypes, validating triumphant detection of Scissors+ cells representing high‐risk clinal status, as well as solidifying the risk‐stratify ability of Anoikis.Sig (Figure 13e). Next, we employed pseudotime analysis by the Monocle 2 algorithm to discover the temporal sequences of intercellular differentiation in epithelial cells, with diverse pseudotime scores, epithelial subtypes, states, and samples (Figures 13f, 13g, 13h, 13i, and 13j). We then used median Anoikis.Sig risk score to categorize epithelial cells as high‐risk and low‐risk states. Comparing pseudotime scores demonstrated that high‐risk epithelial cells owned a more original differential state than low‐risk epithelial cells, indicating that immature epithelial cells with higher risk scores could possibly exhibit as malignant cells (Figure 13k). Interestingly, we employed the inferCNV algorithm to investigate the clone structures of epithelial cells, reminding that high‐risk cells significantly got more CNVs (Figure 13l).

Figure 13Exploration of Anoikis.Sig risk scores in the combined scRNA‐seq cohort. (a–d) Visualizing Anoikis.Sig risk scores computed by four scRNA‐seq scoring algorithms in a UMAP plot. Comparisons of Anoikis.Sig risk scores computed by four scRNA‐seq scoring algorithms among various cell subtypes in a boxplot. (e) Comparisons of Anoikis.Sig risk scores computed by four scRNA‐seq scoring algorithms among Scissor+ epithelial cells, Scissor− epithelial cells, and various cell subtypes in a boxplot. (f–j) Pseudotime trajectory analysis in epithelial subgroups via the Monocle 2 algorithm (cells are colored according to pseudotime, cell types, states, and samples). (k) Pseudotime trajectory analysis based on the Monocle 2 algorithm revealed significant differences of pseudotime scores between high‐risk epithelial cells and low‐risk epithelial cells (cells are colored in single‐cell scoring results by AddModuleScore and high‐risk or low‐risk groups). (l) Significant differences of CNVs in high‐risk epithelial cells and low‐risk epithelial cells.

**Figure:**
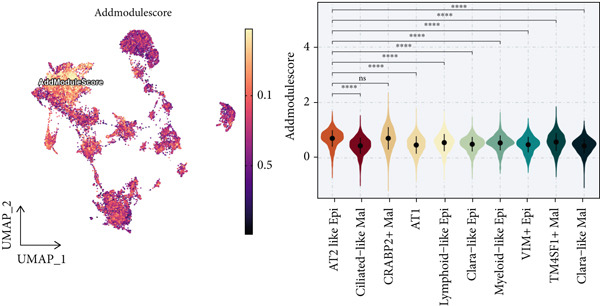
(a)

**Figure:**
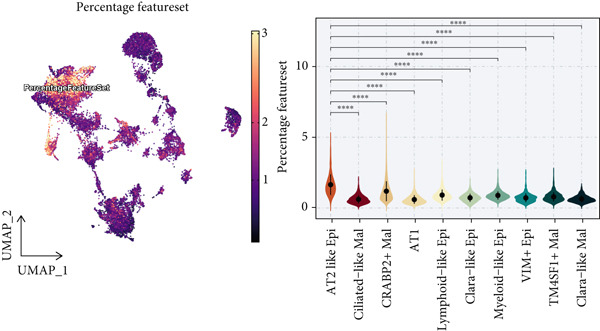
(b)

**Figure:**
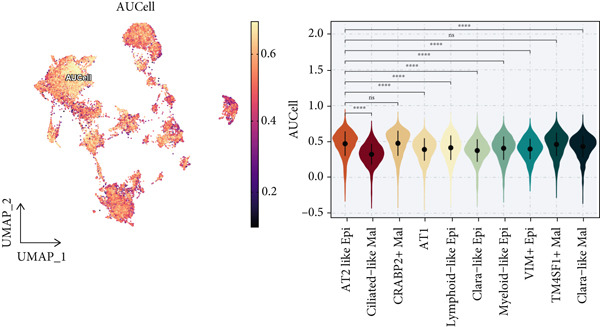
(c)

**Figure:**
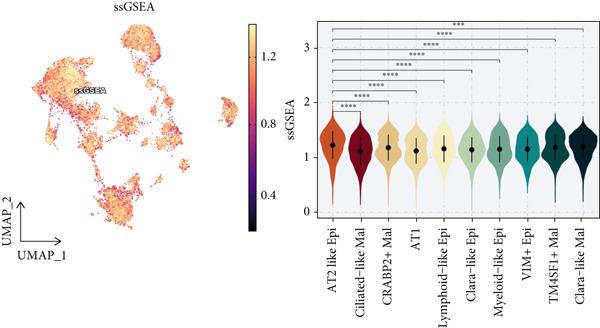
(d)

**Figure:**
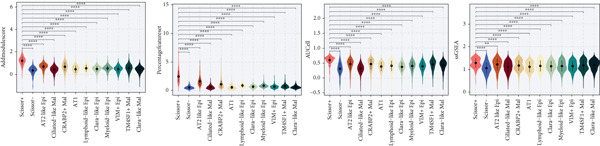
(e)

**Figure:**
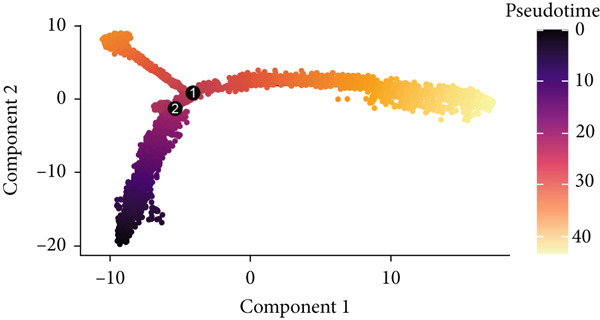
(f)

**Figure:**
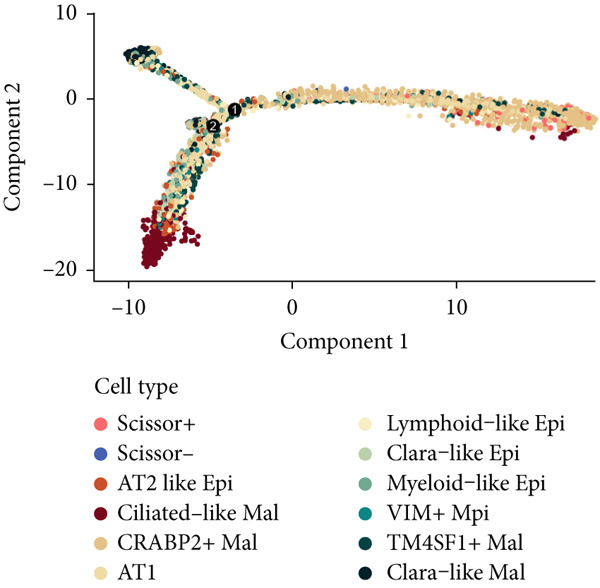
(g)

**Figure:**
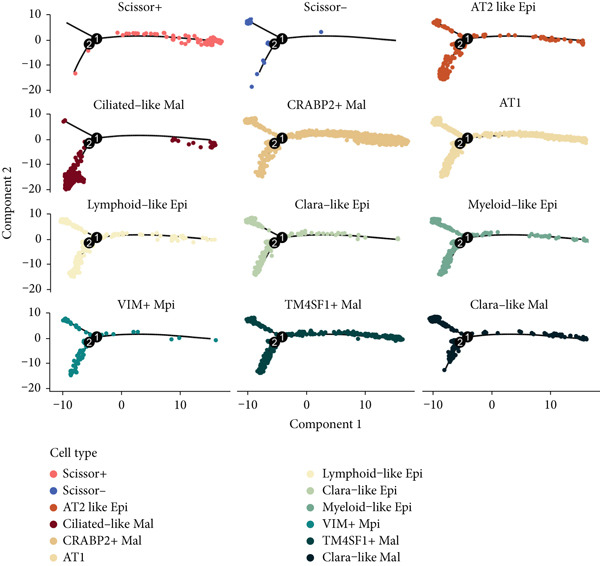
(h)

**Figure:**
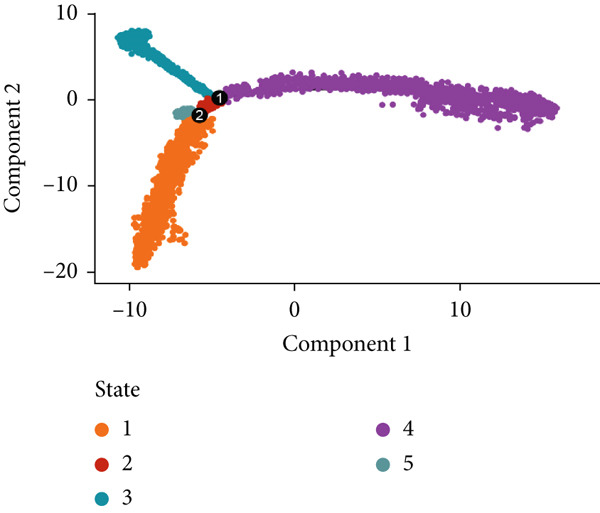
(i)

**Figure:**
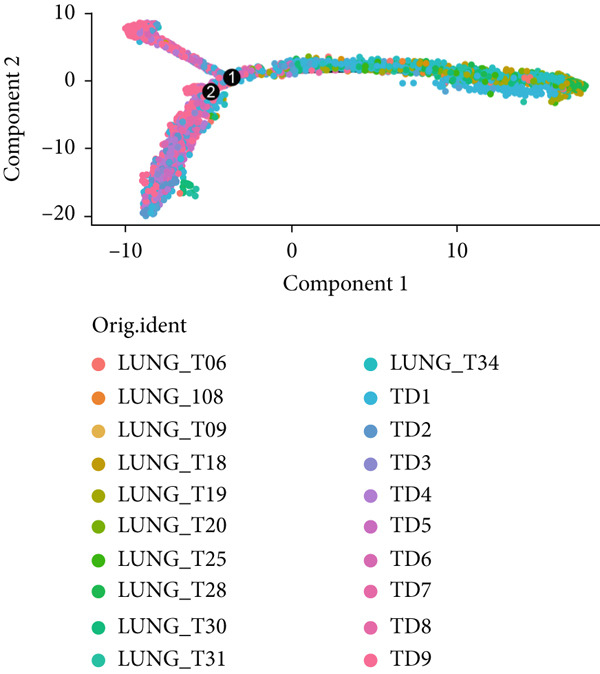
(j)

**Figure:**
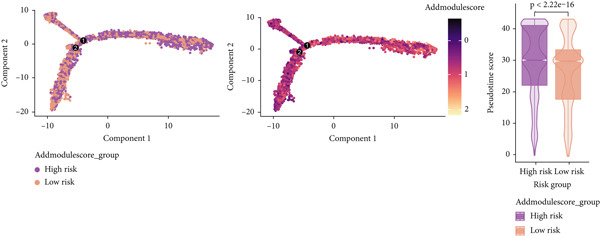
(k)

**Figure:**
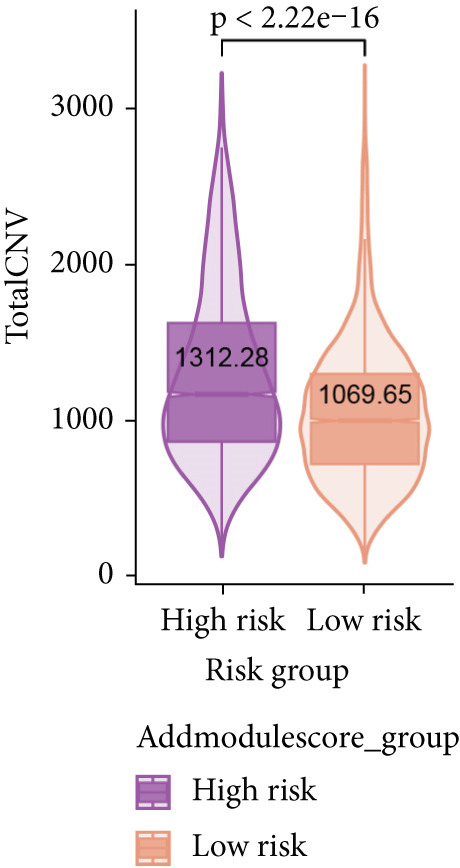
(l)

### 3.14. Experimental Validation of Hub Genes

Our aforementioned bioinformatics analysis has demonstrated that the Anoikis.Sig model genes were powerful in the prognosis prediction of LUAD patients and showed huge research value in LUAD. Indeed, we further illustrated the diverse mRNA expressions of those model genes in tumor and normal tissues in the TCGA‐LUAD cohort (Figure 14a). Survival analysis also verified the prognosis prediction value of those model genes in the TCGA‐LUAD cohort (Figure 14b). Furthermore, the distinct protein abundances of those model genes in tumor and normal tissues were deeply analyzed, supporting the abnormal expressions and dysregulated transcription of those probable oncogenes (Figure 14c). Ultimately, we carefully chose LDHA and KRT7 to explore their oncogene role in LUAD, given that they were significantly upregulated in tumor tissues in both mRNA and protein levels, while they were risky genes based on survival analysis. To further verify the malignant biological behaviors of LDHA and KRT7 in LUAD, we employed qRT‐PCR to corroborate our above bioinformatics findings, supporting their elevated mRNA expressions in tumoral samples (Figure 14d). Our in‐house survival data also validated the risky prognosis role of LDHA and KRT7 in LUAD, which revealed that patients with high mRNA expressions would suffer worse outcomes (Figure 14e). Overall, these results indicate that LDHA and KRT7, model genes of Anoikis.Sig, were important oncogenes with risky prognosis value in LUAD, which need to be thoroughly investigated in future research.

Figure 14Clinical validation of hub genes in Anoikis.Sig. (a) Different mRNA expressions of Anoikis.Sig model genes in tumor tissues and normal tissues in the TCGA‐LUAD cohort. (b) Survival analysis of Anoikis.Sig model genes illustrated by KM curves in the TCGA‐LUAD cohort. (c) Different protein expressions of Anoikis.Sig model genes in tumor tissues and normal tissues in the CPTAC database. (d) qRT‐PCR analysis of mRNA expressions of two hub genes in tumor tissues and normal tissues. (e) KM curves of the high mRNA expression group and the low mRNA expression group in tumor samples.  ^∗^,  ^∗∗^,  ^∗∗∗^, and  ^∗∗∗∗^ indicate a significance level of 0.05, 0.01, 0.001, and 0.0001, respectively.

**Figure:**
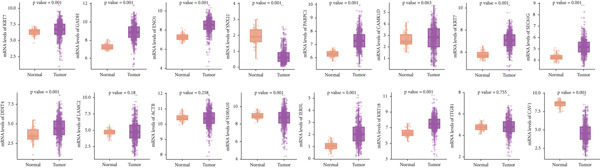
(a)

**Figure:**
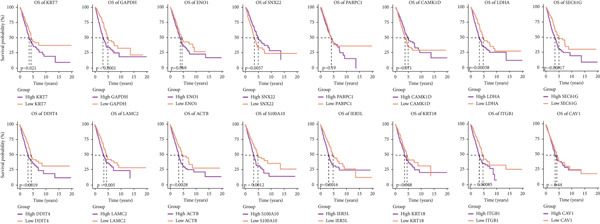
(b)

**Figure:**
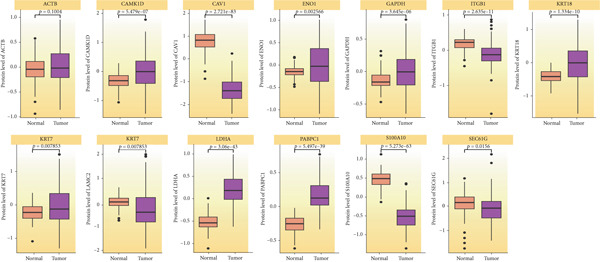
(c)

**Figure:**
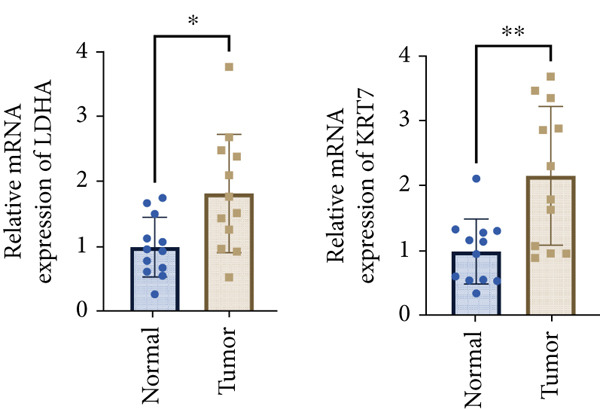
(d)

**Figure:**
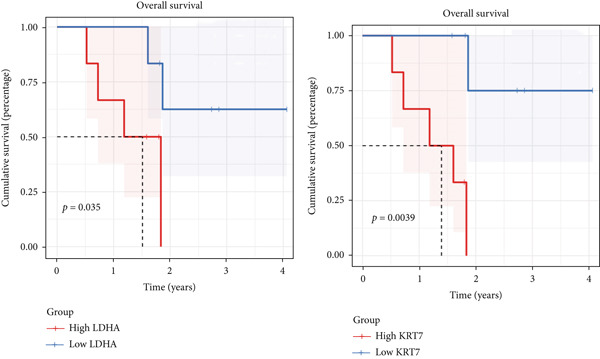
(e)

## 4. Discussion

LUAD represents a complicated and extensively variable cancer, rendering the prognostic forecast for LUAD patients extremely difficult [42]. Prognostic indicators are of great significance in forecasting disease progression, choosing suitable therapy strategies, and forecasting outcomes. Across these vital prognostic elements, histological subtypes and grades hold utmost importance. Higher grade is linked to more invasive cancer behavior, worse PFS, and reduced OS. Molecular indicators have emerged as potent predictors of prognosis in LUAD. The age at the time of diagnosis is also a pivotal factor affecting patient outcome, as younger patients typically have a longer OS in comparison to older ones. The size and location of the tumor also carry prognostic significance, as smaller tumor size and more favorable locations are associated with favorable prognoses. The presence of residual tumor following surgical resections is yet another crucial variable impacting prognoses. Given that our knowledge of LUAD biology deepens, nascent prognostic variables and new molecular indicators are currently being explored to enhance prognostic forecasting and personalized therapy methods.

In recent research, connections between the development mechanisms of LUAD and anoikis have been brought to light. Nevertheless, the possible part that anoikis‐induced cell modulation plays in tumor formation has not been extensively explored. ML techniques are being increasingly adopted in predicting patient survival in cancer research [43], which serves as an essential tool to utilize intricate algorithms to remarkably deal with extensive and variant data. This tool truly excels in prediction‐based activities, with its strength manifesting when recognizing crucial trends and extracting meaningful insights from data, enabling it to perform at its best [44]. However, efficiently applying these methods in clinical practice while ensuring accuracy remains a challenge. Two key aspects to consider are the reason for using a particular ML algorithm and which solution is optimal. The algorithm selection by researchers may be largely influenced by their private inclinations and partialities [45]. In our research, we gathered expression landscapes of LUAD patients covering seven global multicenter cohorts and constructed an Anoikis.Sig model with an innovative computational structure. The Anoikis.Sig is built upon the mRNA expression of anoikis‐related model genes that are most efficacious in forecasting patient outcomes. Enrichment analyses indicated that the high‐risk group is mainly enriched in diverse cellular functions, environmental processing, and organismal systems. Interestingly, our research discovered a substantial relation between the high‐risk group and biological processes associated with DNA replication, cell cycle, and various proliferation‐related pathways. Those results offer a partial explanation for the worse prognosis seen in this group. Our model validations imply that Anoikis.Sig can function as a valuable instrument for guiding treatment decisions and improving patient outcomes. Significantly, Anoikis.Sig can effectively distinguish the prognosis of patients in immunotherapy cohorts, offering clinicians more reliable patient stratification and aiding in identifying those most appropriate for immunotherapy.

We successfully implemented an integrative ML framework to develop and verify the Anoikis.Sig based on the mRNA expression profiles of marker genes of anoikis‐related epithelial cells, which illustrated superior performances in predicting prognosis and forecasting immunotherapy response. A total of 101 prognosis ML algorithm combinations were employed in the train and the test sets according to the LOOCV framework. Comprehensive explorations in seven LUAD cohorts demonstrated that the most powerful ML combination was RSF in feature selection and model establishment. The solidness of this integrative framework is due to its ability to assemble multiple ML algorithms for election, thus triumphantly constructing models with outperforming prognostic prediction capabilities after sifting through every ML combination. This unified system streamlines the model for functional and translational utilization by decreasing the dimensionality of multiple indicators in the process of feature selection. The superior ability of Anoikis.Sig was then validated by time‐dependent AUC values, ROC curves and corresponding AUC values, survival analysis, Cox regression analysis, calibration curves, and DCA curves, which pointed out its supremacy over other models and clinical information in accuracy and stability. Additionally, comparisons of C‐index across seven LUAD cohorts, as well as a meta‐analysis of C‐index altogether, demonstrated that Anoikis.Sig displayed the top precision and robustness among external validation cohorts compared to other signatures, suggesting its vital prospects in translational research and assisting decision‐making.

Immunotherapy offers new opportunities for LUAD patients with malignancies to extend their survival, thereby giving a glimmer of hope to those struggling with this difficult disease [46]. By analyzing the interaction between Anoikis.Sig risk scores and the TME, we discovered an inverse link between Anoikis.Sig risk scores and the majority of immunocytes and immunomodulators, as well as the gene expressions of immune checkpoint genes. Enrichment analysis further emphasized the prevalence of immunologically important functions in the low‐risk group. As a result, individuals with lower risk scores would show “immune hot” characteristics, manifested by increased immunocyte infiltration. It is worth mentioning that previous studies have suggested a beneficial association between increased infiltration of most immune cells in the TME and better prognosis for LUAD, indicating their potential role in suppressing tumor growth [47]. Therefore, this finding may partially explain why patients with high risk scores often have worse prognoses. Significantly, cases with low risk scores had remarkably lower TIDE scores, suggesting that the TIDE algorithm predicts greater sensitivity to immunotherapy in this group. This hypothesis was confirmed in those four pancancer immunotherapy cohorts, highlighting the crucial role of Anoikis.Sig risk as a predictive indicator for immunotherapy efficacy in LUAD and pancancer landscapes.

Furthermore, using the combined scRNA‐seq dataset of LUAD, we further explored the detailed profiles of Anoikis.Sig risk scores on every single cell. Our scRNA‐seq scoring results showed that the AT2‐like Epi had the highest risk scores, confirming their malignant functions in the tumorigenesis of LUAD. Subsequently, with clinical phenotypes from RNA‐seq datasets, we separated high‐risk and low‐risk patients into different phenotypes and employed the Scissor algorithm to map these phenotypes onto scRNA‐seq data, identifying epithelial cells closely related to each risk status. The resulting Scissor− and Scissor+ phenotypes successfully corresponded to low‐ and high‐risk statuses, respectively. Notably, Scissor− cells had lower risk scores compared to Scissor+ cells, which further verified the consistency between Scissor± and high‐/low‐risk statuses and the stable risk‐stratify ability of Anoikis.Sig. We next employed pseudotime trajectory analyses and utilized the inferCNV technique, respectively, in high‐risk and low‐risk cells, which depicted vital disparities within intercellular differentiation and CNV landscapes between the two risk groups. We discovered that epithelial cells with higher risk scores were mostly immature and malignant epithelial cells with more CNVs, suggesting the fine capability of Anoikis.Sig to risk‐stratify LUAD epithelial cells, as well as the diverse single‐cell transcriptome landscapes between the two risk groups.

In stRNA‐seq analysis, the finding that the AT2‐like subpopulation was in closest spatial proximity to endothelial cells, B cells, and NK cells suggests a specialized niche for local immune interaction. This architecture may be critical for facilitating the exchange of signals between the lung epithelium and the immune system. The proximity to endothelial cells could imply a role in leukocyte trafficking or the reception of systemic signals. Furthermore, the close association with B and NK cells points to a potential role for this AT2‐like subpopulation in modulating adaptive and innate immune responses, possibly through antigen presentation or the secretion of specific chemokines/cytokines that create a localized immune microenvironment. While scRNA‐seq and stRNA‐seq provide invaluable high‐throughput gene expression data, they inherently lack direct experimental validation of the spatial relationships between cells. Future validation efforts should integrate experimental approaches that can directly assess the spatial distribution of anoikis‐related regulators. Multiplex immunofluorescence (mIF) is a powerful technique that can provide detailed spatial information about protein expression and cell localization. By using mIF, researchers can visualize and quantify the spatial relationships between different cell types and their expression of anoikis‐related regulators.

LUAD exhibits distinct metabolic and molecular features, while two biomarkers, Lactate Dehydrogenase A (LDHA) and Keratin 7 (KRT7), have emerged as significant players in LUAD pathogenesis, diagnosis, and therapeutic targeting. LDHA, a key glycolytic enzyme, catalyzes pyruvate‐to‐lactate conversion under hypoxia, fueling the Warburg effect observed in 80% of LUAD cases [48]. Researchers have found that LDHA maintains NAD+ regeneration for glycolytic flux, with siRNA‐mediated knockdown suppressing tumor cell proliferation in vitro [49]. And high LDHA levels correlate with increased invasion and metastasis promotion via epithelial–mesenchymal transition (EMT) and autophagy [50]. In translational medicine, researchers indicated that targeting LDHA might be a prospective therapy to enhance radiotherapy effectiveness in NSCLC patients, which needs to be deeply validated by clinical trials [51]. KRT7, alternatively referred to as cytokeratin‐7 (CK‐7) or K7, represents a major component of the intermediate filament cytoskeleton. This protein is predominantly found in simple epithelial tissues. These tissues form the inner linings of various structures, including the cavities within internal organs, glandular ducts, and blood vessels [52]. KRT7 is highly expressed in 90%–95% of LUADs but is absent in squamous cell carcinoma, which could be used as a diagnostic differentiation [53]. In triple‐negative breast cancer, the expression of KRT7 is abnormal and may be related to the invasion and metastasis of the tumor [54]. Previous studies have shown that KRT7 mRNA expression measured by qRT‐PCR could be a sensitive technique for the molecular detection of KRT7‐positive circulating tumor cells (CTCs) resembling A549 cells in peripheral whole blood of LUAD patients. In summary, these researches have provided meaningful insights into the oncogenic roles of LDHA and KRT7 in LUAD, which need to be further investigated for their translational value in clinical practices.

Our Anoikis.Sig can be easily replicated through qRT‐PCR detection techniques, making it viable for wider clinical application. Sequencing the transcriptome of LUAD samples obtained from biopsies or surgeries could serve as a routine predictive tool to inform treatment strategies. With this data integrated into Anoikis.Sig, clinicians could accurately assess prognosis and design tailored treatments for patients facing challenging outcomes. However, our study has several limitations. First, we collected retrospective sequencing datasets and relevant clinical information from public repositories, which need a large‐scale, multicenter prospective validation. The absence of detailed treatment strategies, metastatic sites, and recurrence information might impact our results. Second, the characteristics of these two hub genes in LUAD remain undetermined. Thus, additional research with more tumor samples and in vitro or in vivo experiments is needed to explore its biological functions in LUAD. Finally, our current model‐building approach, relying solely on transcriptome sequencing, could greatly benefit from integrative multimodal data analysis. Such comprehensive integration allows for a more in‐depth understanding of molecular mechanisms and physiological processes, enhancing the reliability and accuracy of prediction models. Incorporating multimodal data adds numerous variables to the analysis, which is essential for more complex artificial intelligence models. Deep learning, a subfield of ML, can independently identify key classification features, unlike traditional ML techniques that require manual feature selection and input. Therefore, using novel deep learning algorithms, combined with the advantages of multimodal data integration, represents a potent strategy for advancing individual treatment for LUAD patients.

## 5. Conclusion

Ultimately, we succeed in constructing the Anoikis.Sig to precisely predict prognosis and forecast immunotherapy in LUAD patients, based on a robust ML algorithm framework. By numerous verifications in model performances, immune microenvironment, mutation landscapes, immunotherapy assessment, chemotherapy response, scRNA‐seq, and stRNA‐seq analysis, Anoikis.Sig shows both solidness and accuracy in outcome forecasting, serving as a superior predictive model in LUAD. Furthermore, we investigate two Anoikis.Sig hub genes of LDHA and KRT7 with abundant research value, which depicts their oncogene roles in LUAD, suggesting underlying research opportunities in the future.

## Ethics Statement

The study was conducted according to the guidelines of the Declaration of Helsinki and approved by the Ethics Committee of Huai′an Hospital of Huai′an City.

## Consent

The authors have nothing to report.

## Disclosure

All authors contributed to the article and approved the submitted version.

## Conflicts of Interest

The authors declare no conflicts of interest.

## Author Contributions

Shan Li designed the research plan, performed bioinformatics analysis, data collection, assembly of data, conducted data analysis, and wrote the manuscript. Ting Chen and Chen Hu performed experiments and finalized the manuscript. Wenhang Zhou and Jinping Li supervised the research progress and revised the manuscript.

## Funding

No funding was received for this manuscript.

## Supporting Information

Additional supporting information can be found online in the Supporting Information section.

## Supporting information


**Supporting Information 1** Figure S1: (A) Harmony algorithm reduced the batch effects of each sample in the combined scRNA‐seq cohort of LUAD. (B) Quality control of each cell type in the combined scRNA‐seq cohort of LUAD. (C) The expression profiles of anoikis‐related genes in the combined scRNA‐seq cohort of LUAD. (D) Harmony algorithm reduced the batch effects of epithelial cells in the combined scRNA‐seq cohort of LUAD.

**Supporting Information 2** Figure S2: (A) Violin plot visualized the marker gene expression in LUAD epithelial clusters. (B) The UMAP plot visualized the marker gene expression in LUAD epithelial clusters. (C) AddModuleScore and PercentageFeatureSet algorithms calculated the single‐cell scoring results of epithelial cells with the anoikis gene set. (D) Stemness within the epithelial subpopulations was assessed using CytoTRACE analysis, with higher CytoTRACE scores indicating increased stemness. CytoTRACE scores were mapped onto individual cells in a UMAP to provide a more intuitive representation of stemness variation across different epithelial subpopulations. (E) The expression profiles of anoikis‐related genes across different epithelial subpopulations.

**Supporting Information 3** Figure S3: (A) Pseudotime DEGs identified by pseudotime analysis. (B) Survival analysis of TCGA‐LUAD patients with high CD8A or low CD8A, as well as four groups of patients divided by median CD8A expression and AT2‐like Epi scores. (C) The bubble chart showed differences in communication signals among various cell subpopulations. Bubble size represents *p* value generated by the permutation test, and the color represents the possibility of interactions. (D) Top 3 Hallmark terms of every epithelial subtype by GSVA. (E) The heatmap showed the efferent or afferent contributions of all signals to different cell types. (F) Heatmap showing incoming and outgoing interactions among various cell subtypes.

**Supporting Information 4** Figure S4: (A) Quality control of stRNA‐seq data. (B) Spatial map showing 13 clusters identified by stRNA‐seq. (C) Identification of cell types and proportions in each spot through deconvolution methods, with spatial maps showing the second abundant cell type in each spot.

**Supporting Information 5** Figure S5: (A) PCA plot shows the successful removal of batch effects among LUAD RNA‐seq cohorts. (B) The flowchart to schematically explain the algorithmic pipeline of machine learning algorithm integration. (C) Visualization of logarithmic loss, recall, and decision calibration of Top 5 prognostic machine learning models.

**Supporting Information 6** Supporting methods: These methods were previously described in our previous study [28].

## Data Availability

Publicly available datasets were analyzed in this study. The names of the repositories and accession numbers can be found within the article/supporting information. The data that support the findings of this study are available on request from the corresponding authors. The data are not publicly available due to privacy or ethical restrictions.
