# Supplementary figures and images for: Advancing Prognosis Prediction and Immunotherapy Efficacy in Lung Adenocarcinoma Through Machine Learning: Novel Insights From Anoikis Regulator Patterns in Single‐Cell Multiomics
Source: Int J Genomics. 2026 Jan 3;2026:9458552. doi: 10.1155/ijog/9458552 (PMC12764181; doi:10.1155/ijog/9458552)

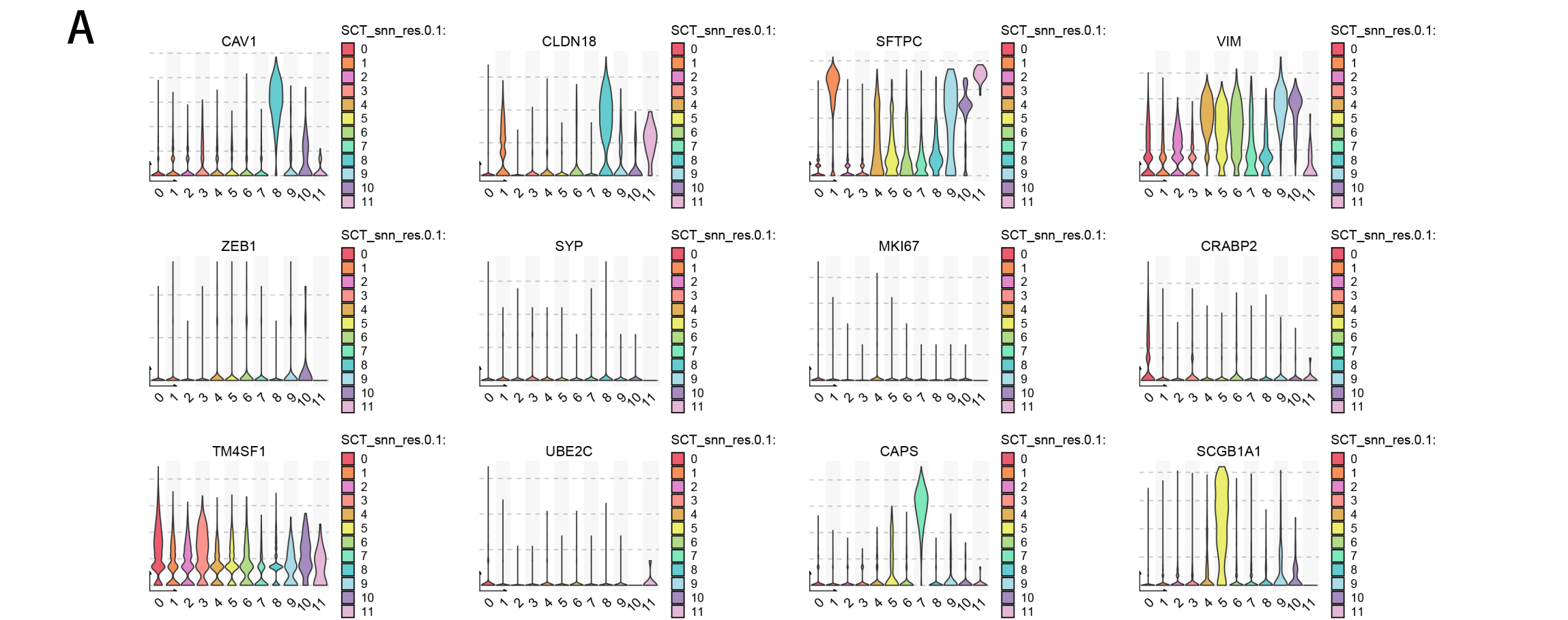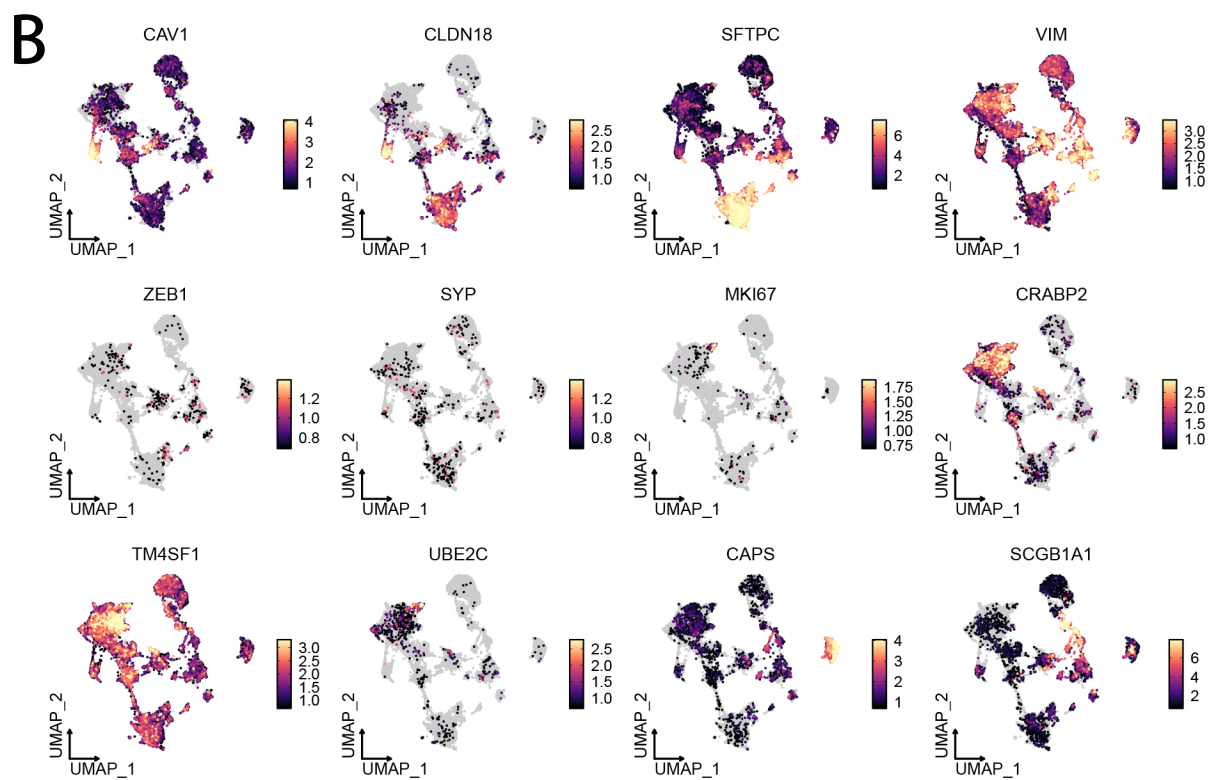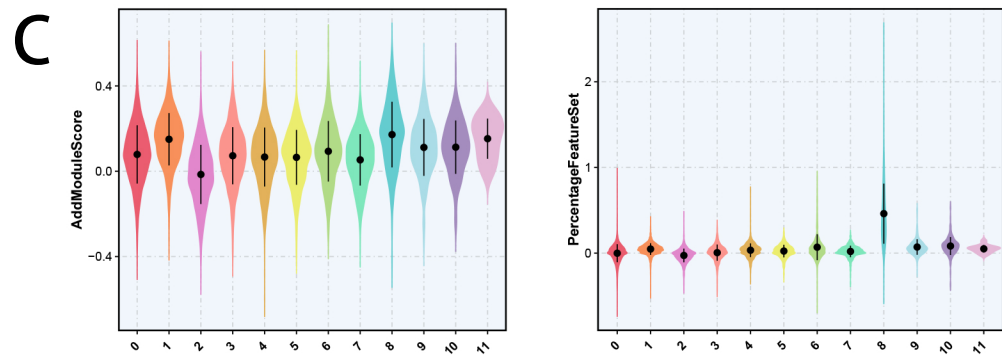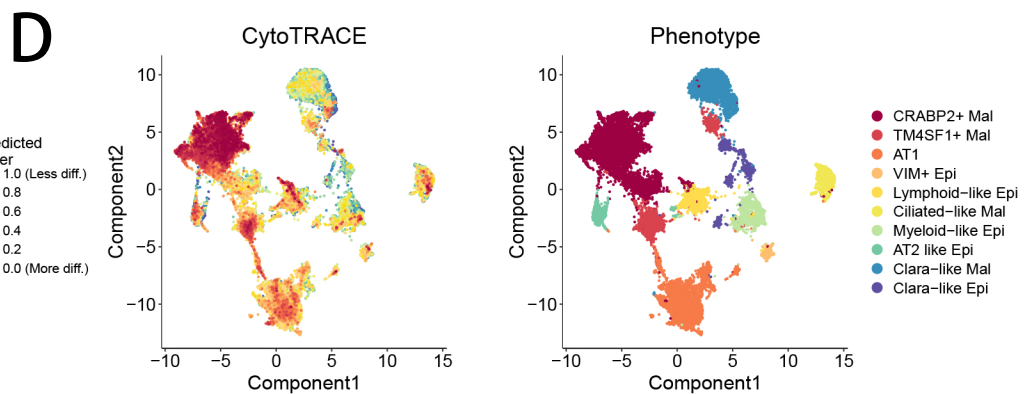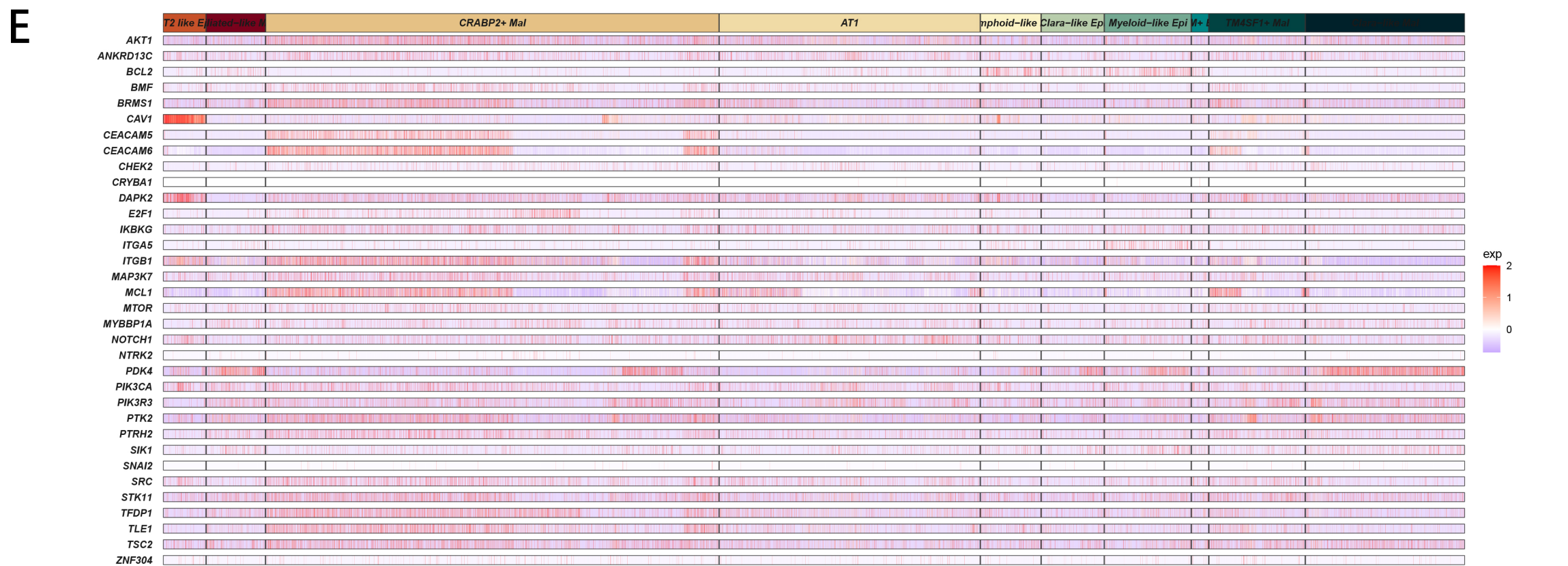

Supplement: Supplementary file 2 — Supporting Information 2 Figure S2: (A) Violin plot visualized the marker gene expression in LUAD epithelial clusters. (B) The UMAP plot visualized the marker gene expression in LUAD epithelial clusters. (C) AddModuleScore and PercentageFeatureSet algorithms calculated the single‐cell scoring results of epithelial cells with the anoikis gene set. (D) Stemness within the epithelial subpopulations was assessed using CytoTRACE analysis, with higher CytoTRACE scores indicating increased stemness. CytoTRACE scores were mapped onto individual cells in a UMAP to provide a more intuitive representation of stemness variation across different epithelial subpopulations. (E) The expression profiles of anoikis‐related genes across different epithelial subpopulations. [file IJOG-2026-9458552-s002.pdf]

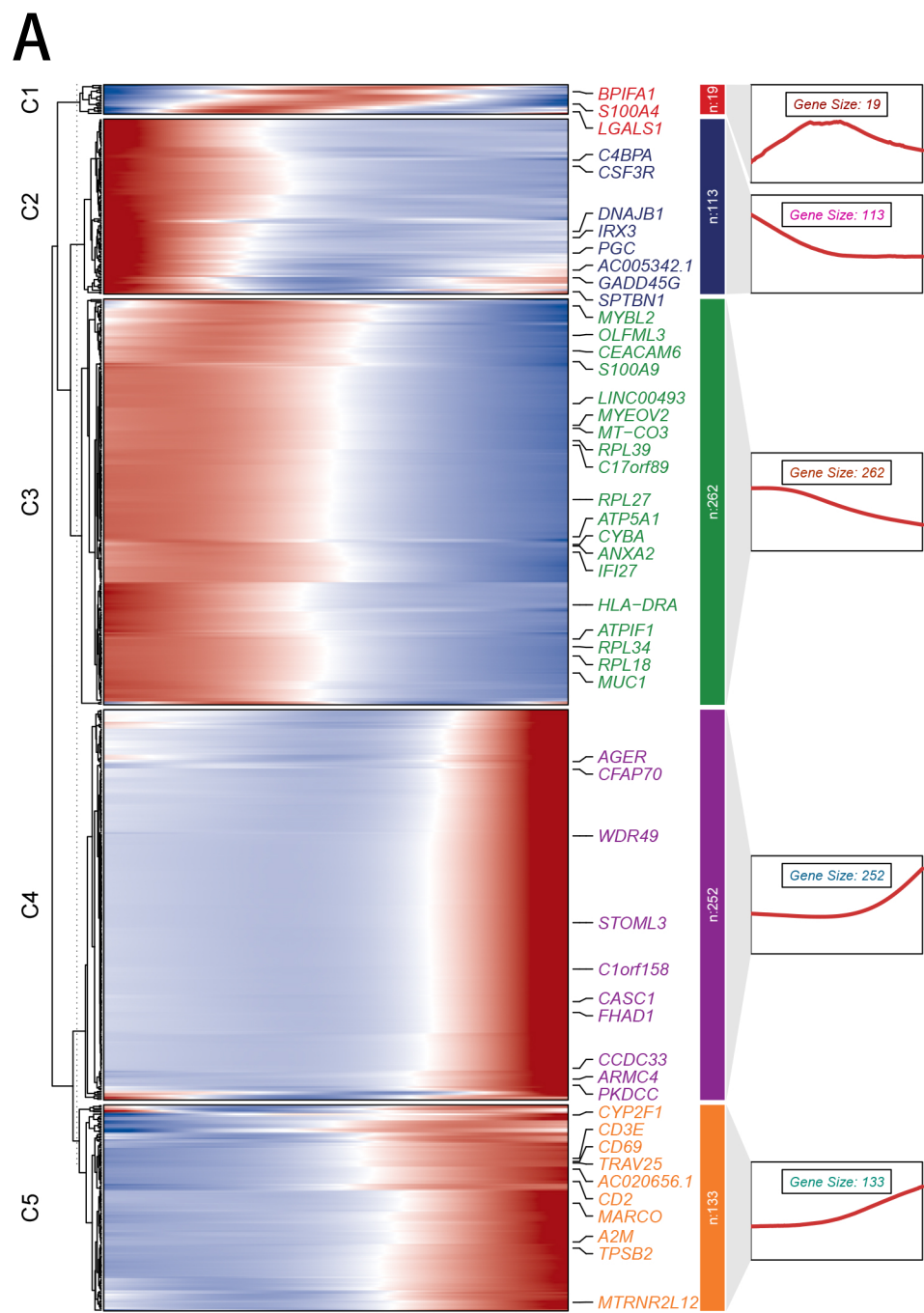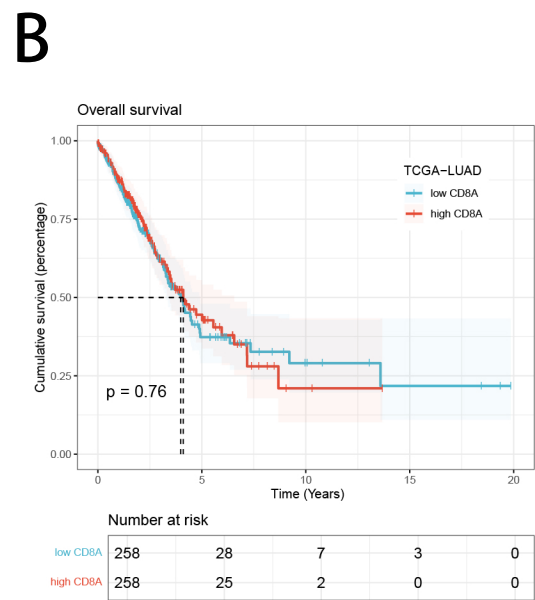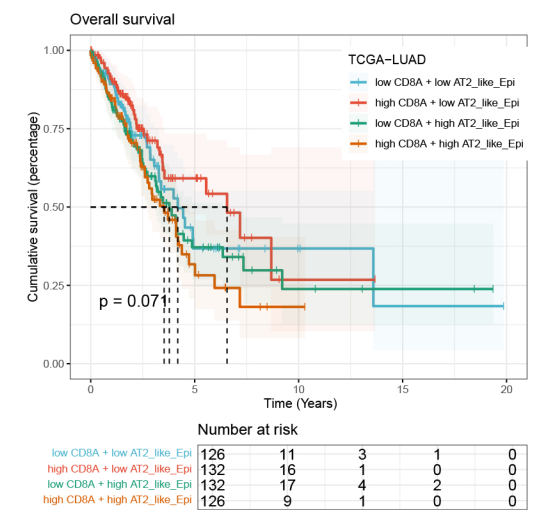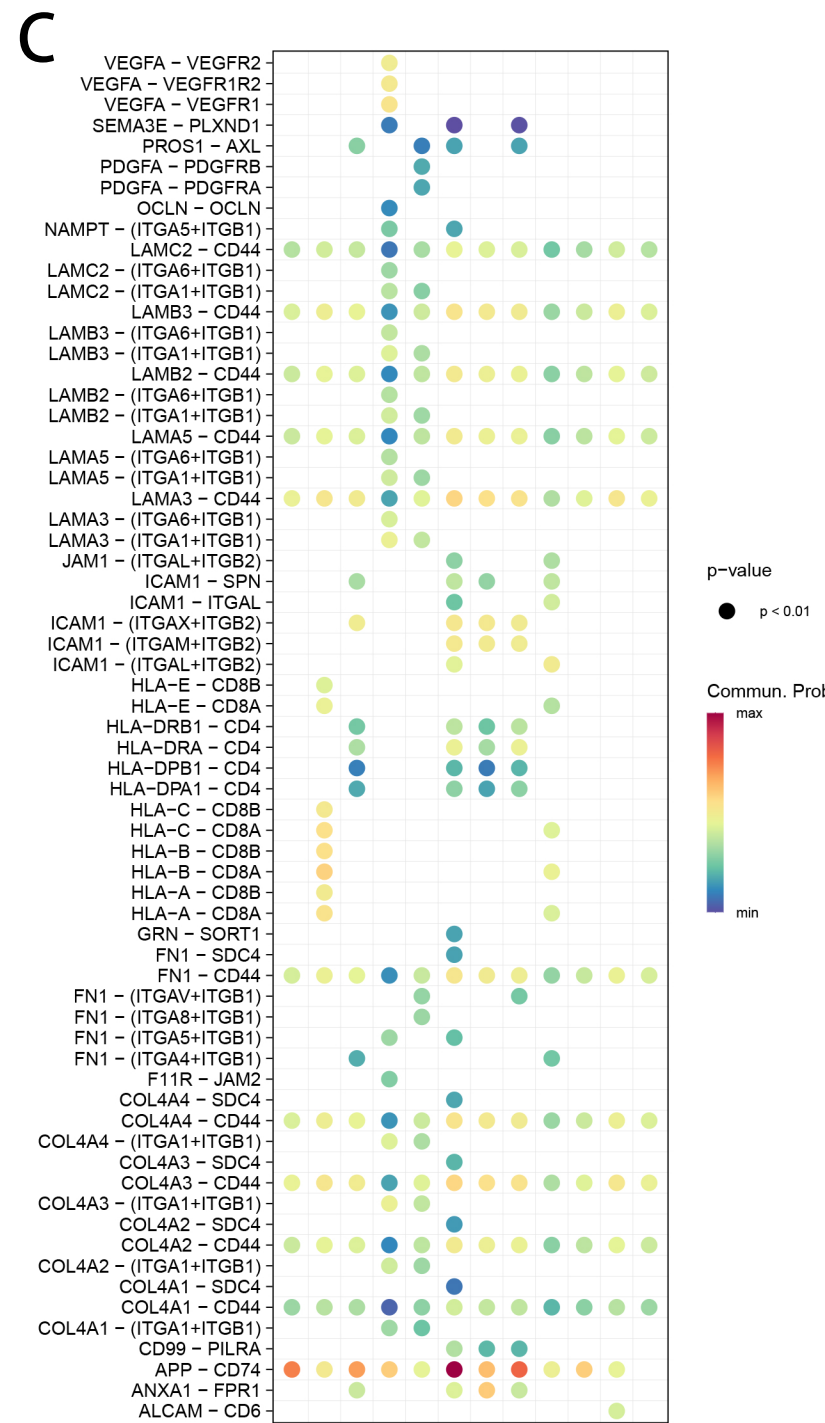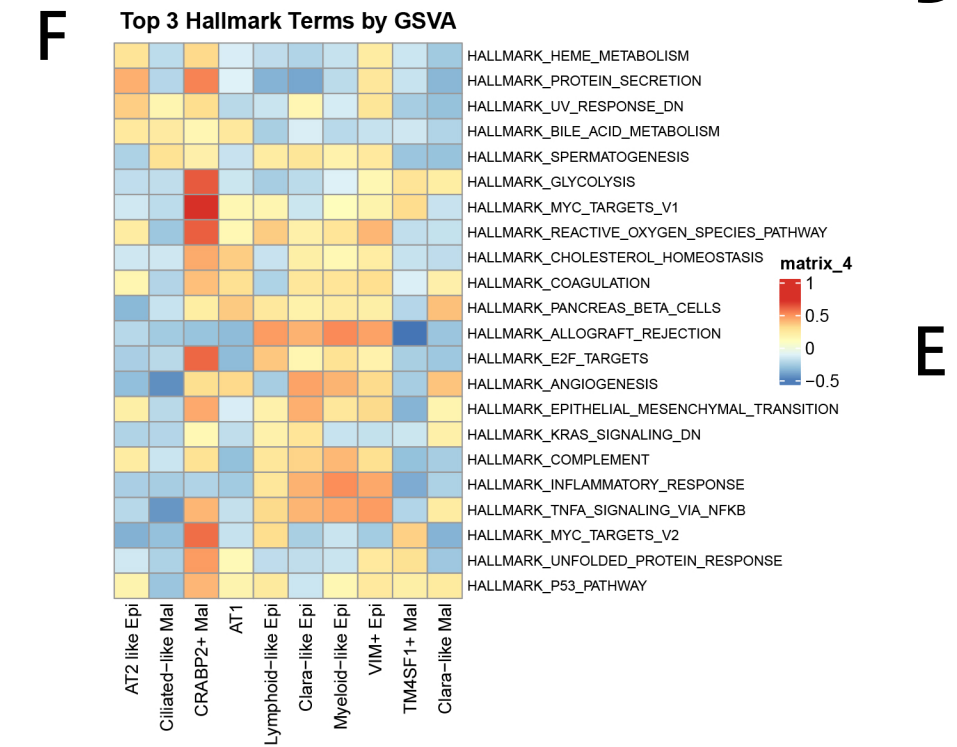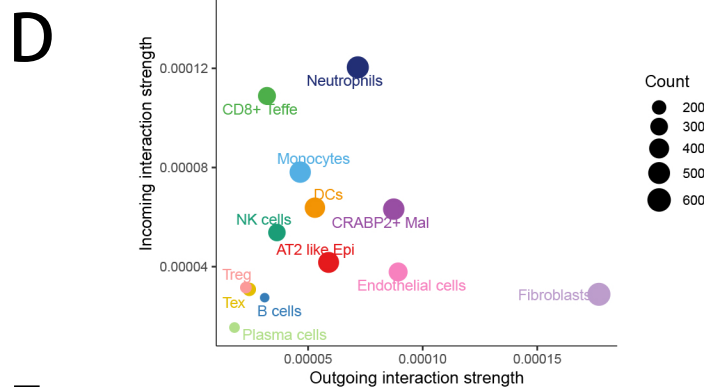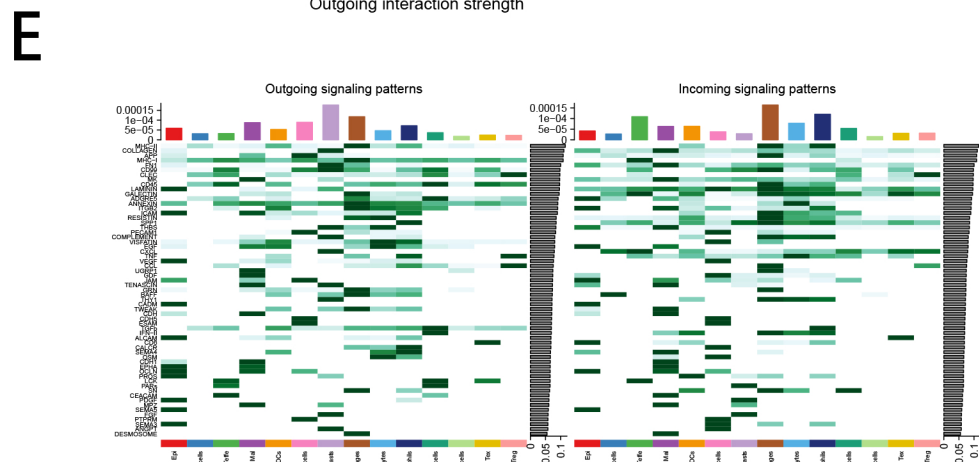

Supplement: Supplementary file 3 — Supporting Information 3 Figure S3: (A) Pseudotime DEGs identified by pseudotime analysis. (B) Survival analysis of TCGA‐LUAD patients with high CD8A or low CD8A, as well as four groups of patients divided by median CD8A expression and AT2‐like Epi scores. (C) The bubble chart showed differences in communication signals among various cell subpopulations. Bubble size represents p value generated by the permutation test, and the color represents the possibility of interactions. (D) Top 3 Hallmark terms of every epithelial subtype by GSVA. (E) The heatmap showed the efferent or afferent contributions of all signals to different cell types. (F) Heatmap showing incoming and outgoing interactions among various cell subtypes. [file IJOG-2026-9458552-s006.pdf]

A

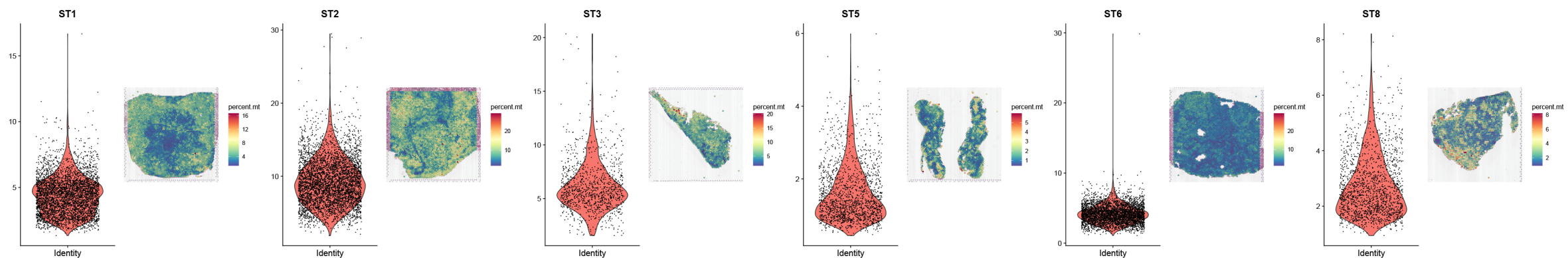

B

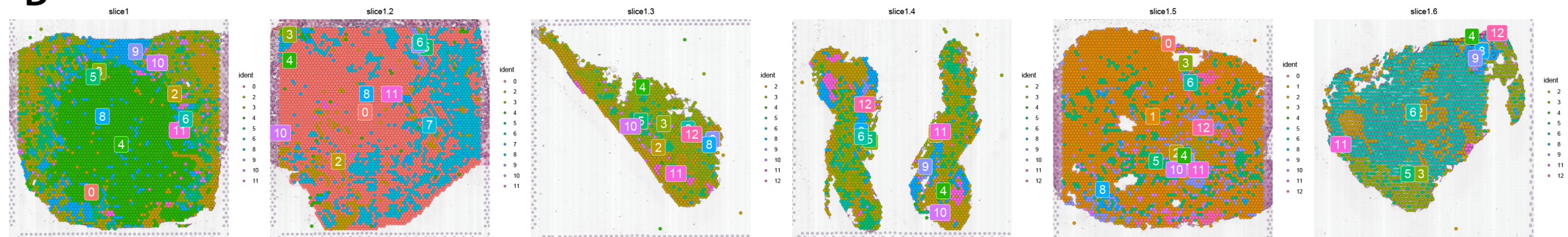

C

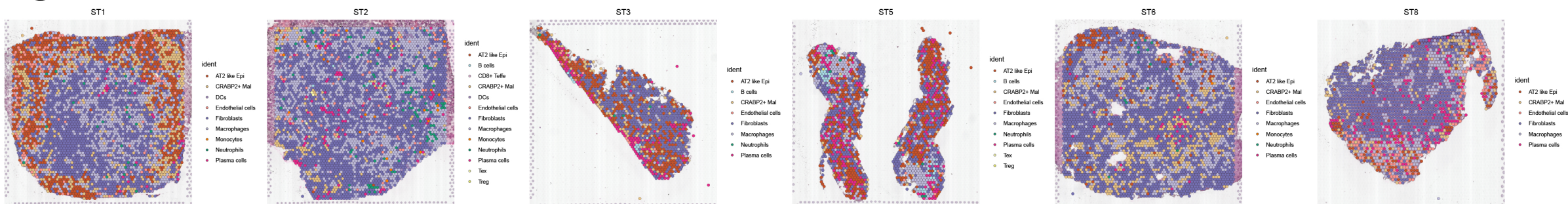

Supplement: Supplementary file 4 — Supporting Information 4 Figure S4: (A) Quality control of stRNA‐seq data. (B) Spatial map showing 13 clusters identified by stRNA‐seq. (C) Identification of cell types and proportions in each spot through deconvolution methods, with spatial maps showing the second abundant cell type in each spot. [file IJOG-2026-9458552-s004.pdf]

A

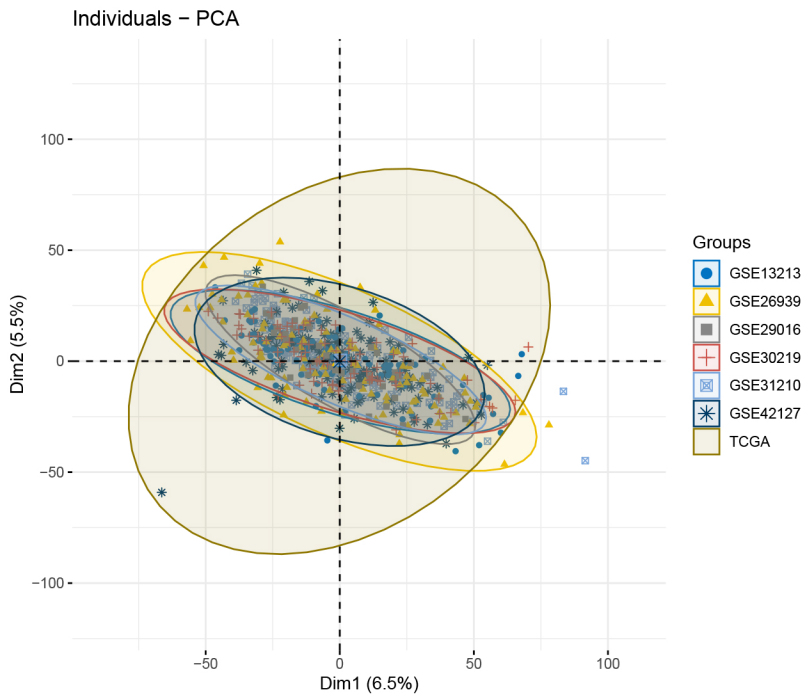

C

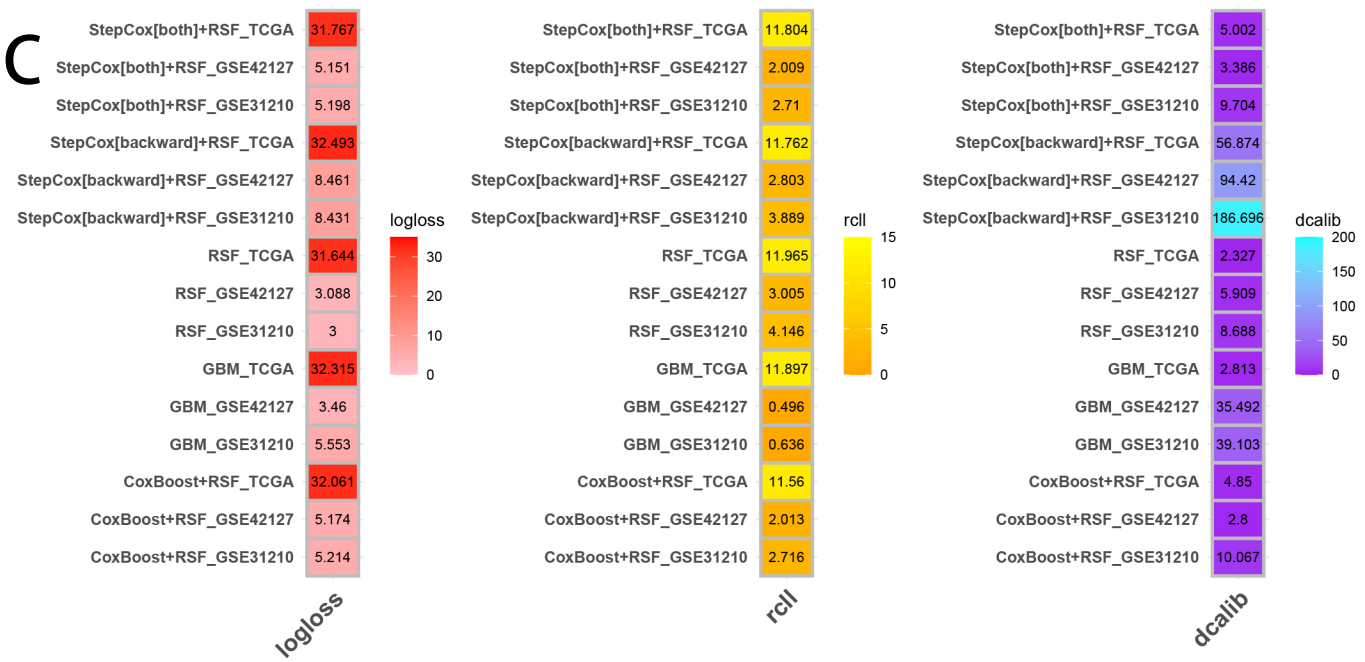

B

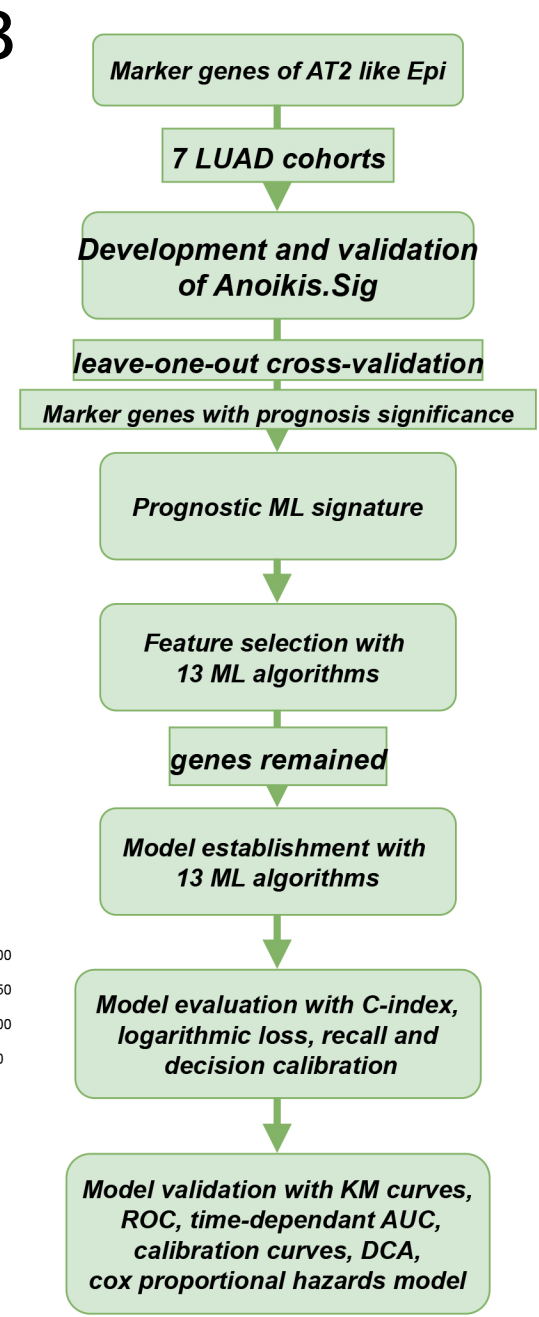

Supplement: Supplementary file 5 — Supporting Information 5 Figure S5: (A) PCA plot shows the successful removal of batch effects among LUAD RNA‐seq cohorts. (B) The flowchart to schematically explain the algorithmic pipeline of machine learning algorithm integration. (C) Visualization of logarithmic loss, recall, and decision calibration of Top 5 prognostic machine learning models. [file IJOG-2026-9458552-s005.pdf]
