# Supplementary material for: Advancing Prognosis Prediction and Immunotherapy Efficacy in Lung Adenocarcinoma Through Machine Learning: Novel Insights From Anoikis Regulator Patterns in Single‐Cell Multiomics
Source: Int J Genomics. 2026 Jan 3;2026:9458552. doi: 10.1155/ijog/9458552 (PMC12764181; doi:10.1155/ijog/9458552)
Supplement: Supplementary file 6 — Supporting Information 6 Supporting methods: These methods were previously described in our previous study [28]. [file IJOG-2026-9458552-s003.docx]

**Advancing prognosis prediction and immunotherapy efficacy in lung adenocarcinoma through machine learning: novel insights from anoikis regulator patterns in single-cell multi-omics**

Authors: Shan Li 1,2, Wenhang Zhou 3, Chen Hu 4, Ting Chen* 3, Jinping Li * 1

**Supplementary Methods**

**These methods were previously described in our previous study [1].**

**Consensus cluster analysis of anoikis patterns**

Base on model genes of Anoikis.Sig, we conducted unsupervised clustering analyses in three LUAD cohorts with“ConsensusClusterPlus” R package and the k-means algorithm [2]. Principal components analysis (PCA) and t-Distributed Stochastic Neighbor Embedding (t-SNE) were implemented to illustrate the heterogeneity between clusters. We utilized the “ComplexHeatmap” R package to assess the efficacy of the clustering analysis. This was done by comparing the disparities in clinicopathological features and gene expression levels among different clusters. Additionally, we carried out a survival analysis to examine the differences in survival outcomes between the clusters.

**Function enrichment analysis**

Differentially expressed genes (DEGs) were discovered between two clusters separated by consensus molecular clustering and between two risk groups divided by Anoikis.Sig. DEGs were defined by "limma" R package, with the threshold of False-discovery rate (FDR) <0.05 and absolute log2fold change (FC) >1. The functional enrichment of DEGs was employed in Gene Ontology (GO) and Kyoto Encyclopedia of Genes and Genomes (KEGG) terms within the "clusterprofiler" R package. [3]. Gene set variation analysis (GSVA) was implemented with KEGG terms within “GSVA” R package [4], based on the “h.all.v7.4.symbols.gmt” gene set from MSigDB. Gene set enrichment analysis (GSEA) was employed to investigate the signaling pathways related to distinct clusters and risk groups [5], with the threshold of p < 0.05 and Normalized Enrichment Score (NES) > 1.

**Delineating tumor microenvironment and immune subtypes**

We utilized several immune abundance algorithms by "IOBR" R package and the single sample gene set enrichment analysis (ssGSEA) with marker genes [6] to appraise the immune infiltration levels between groups and clusters by "wilcox" test [7-15]. Following this, the Spearman correlation analysis was implemented to investigate the relations among risk scores, model gene expressions, and immune cell abundances. Moreover, we implemented ssGSEA to assess the immune function levels with immune function marker genes [16] and to evaluate the seven steps of cancer immunity cycle with related genes in Tracking Tumor Immunophenotype (TIP) (http://biocc.hrbmu.edu.cn/TIP/) [17]. Afterwards, we illustrated the gene expression levels of immune checkpoint genes in two risk groups. We implemented immune subtype analysis [18] and discovered five immunological subtypes in TCGA-LUAD cohort, covering wound healing (C1), IFN-gamma dominant (C2), inflammatory (C3), lymphocyte depleted (C4) and TGF-β dominant (C6). We then assessed the proportions of various immunological subtypes among groups and clusters.

**Mutational landscape and copy number variation**

Acquiring the somatic mutational information from cBioPortal website (https://www.cbioportal.org/), we assessed the mutational categories and frequencies of Anoikis.Sig model genes through “maftools” R package [19]. Following this, we evaluated the tumor mutation burden (TMB) through computing the aggregate count of somatic mutations per megabase (MB) in the exonic region of the human genome. Genetic mutations were categorized into two subtypes, named as synchronous or nonsynchronous mutation. The latter comprised Frame_Shift_Del, Frame_Shift_Ins, In_Frame_Del, In_Frame_Ins, Missense, Nonsense, Nonstop, Splice_Site, and Translation_Start_Site aberrations. Key mutation regions were appraised in the copy number variation (CNV) data from cBioPortal website through GISTIC 2.0 [20]. Afterwards, the frequencies of somatic CNV of model genes were illustrated in “bubble plot”, while the chromosomal locations of genetic mutation was showed in “circle plot” through “RCircos” R package [21].

**Assessment of immunotherapy and chemotherapy**

To evaluate effectiveness of Anoikis.Sig to forecast immunotherapy responsiveness, we assessed immune dysfunction and exclusion (TIDE, http://tide.dfci.harvard.edu/) scores between risk groups. Following this, we employed submap algorithm to investigate immunotherapy response based on an immunotherapy cohort [22, 23]. Afterwards, we evaluated the competence of Anoikis.Sig to forecast immunotherapy response in immunotherapy datasets (IMvigor210, GSE78220, GSE135222, and GSE91061). Meanwhile, we gathered the chemotherapy sensitivity data of human cancer cell lines in Cancer Therapeutics Response Portal (CTRP, <https://portals.broadinstitute.org/ctrp>) and Profiling Relative Inhibition Simultaneously in Mixtures (PRISM, <https://depmap.org/portal/prism/>), to identify potential targets for personalized medicine strategies in LUAD based on “oncoPredict” R package [24]. Cell line which is more responsive to a chemotherapy agent could get a lower AUC, which help explore potential therapeutics for high-risk patients [25].

**Statistical Analysis**

All statistical analyses were performed using R software (version 4.2.1; R Foundation for Statistical Computing). Continuous data are presented as mean ± standard deviation if normally distributed, or as median with interquartile range if not. Normality was assessed using the Shapiro-Wilk test. Categorical variables are presented as numbers (percentages). Differences between two groups for continuous variables were analyzed using the independent two-sample Student's t-test (for normal data) or the Mann-Whitney U test (for non-normal data). Comparisons among three or more groups were conducted using one-way ANOVA with Tukey's post-hoc test. Categorical variables were compared using the Chi-square test or Fisher’s exact test, as appropriate. A two-sided p-value of < 0.05 was considered statistically significant.

1. Li, S., J. Luo, J. Liu and D. He, *Pan-cancer single cell and spatial transcriptomics analysis deciphers the molecular landscapes of senescence related cancer-associated fibroblasts and reveals its predictive value in neuroblastoma via integrated multi-omics analysis and machine learning.* Front Immunol, 2024. **15**: p. 1506256.

2. Wilkerson, M.D. and D.N. Hayes, *ConsensusClusterPlus: a class discovery tool with confidence assessments and item tracking.* Bioinformatics, 2010. **26**(12): p. 1572-3.

3. Yu, G., L.G. Wang, Y. Han and Q.Y. He, *clusterProfiler: an R package for comparing biological themes among gene clusters.* Omics, 2012. **16**(5): p. 284-7.

4. Hänzelmann, S., R. Castelo and J. Guinney, *GSVA: gene set variation analysis for microarray and RNA-seq data.* BMC Bioinformatics, 2013. **14**: p. 7.

5. Subramanian, A., P. Tamayo, V.K. Mootha, S. Mukherjee, B.L. Ebert, M.A. Gillette, et al., *Gene set enrichment analysis: a knowledge-based approach for interpreting genome-wide expression profiles.* Proc Natl Acad Sci U S A, 2005. **102**(43): p. 15545-50.

6. Jia, Q., W. Wu, Y. Wang, P.B. Alexander, C. Sun, Z. Gong, et al., *Local mutational diversity drives intratumoral immune heterogeneity in non-small cell lung cancer.* Nat Commun, 2018. **9**(1): p. 5361.

7. Zeng, D., Z. Ye, R. Shen, G. Yu, J. Wu, Y. Xiong, et al., *IOBR: Multi-Omics Immuno-Oncology Biological Research to Decode Tumor Microenvironment and Signatures.* Front Immunol, 2021. **12**: p. 687975.

8. Newman, A.M., C.L. Liu, M.R. Green, A.J. Gentles, W. Feng, Y. Xu, et al., *Robust enumeration of cell subsets from tissue expression profiles.* Nat Methods, 2015. **12**(5): p. 453-7.

9. Yoshihara, K., M. Shahmoradgoli, E. Martínez, R. Vegesna, H. Kim, W. Torres-Garcia, et al., *Inferring tumour purity and stromal and immune cell admixture from expression data.* Nat Commun, 2013. **4**: p. 2612.

10. Finotello, F., C. Mayer, C. Plattner, G. Laschober, D. Rieder, H. Hackl, et al., *Molecular and pharmacological modulators of the tumor immune contexture revealed by deconvolution of RNA-seq data.* Genome Med, 2019. **11**(1): p. 34.

11. Li, B., E. Severson, J.C. Pignon, H. Zhao, T. Li, J. Novak, et al., *Comprehensive analyses of tumor immunity: implications for cancer immunotherapy.* Genome Biol, 2016. **17**(1): p. 174.

12. Charoentong, P., F. Finotello, M. Angelova, C. Mayer, M. Efremova, D. Rieder, et al., *Pan-cancer Immunogenomic Analyses Reveal Genotype-Immunophenotype Relationships and Predictors of Response to Checkpoint Blockade.* Cell Rep, 2017. **18**(1): p. 248-262.

13. Becht, E., N.A. Giraldo, L. Lacroix, B. Buttard, N. Elarouci, F. Petitprez, et al., *Estimating the population abundance of tissue-infiltrating immune and stromal cell populations using gene expression.* Genome Biol, 2016. **17**(1): p. 218.

14. Aran, D., Z. Hu and A.J. Butte, *xCell: digitally portraying the tissue cellular heterogeneity landscape.* Genome Biol, 2017. **18**(1): p. 220.

15. Racle, J., K. de Jonge, P. Baumgaertner, D.E. Speiser and D. Gfeller, *Simultaneous enumeration of cancer and immune cell types from bulk tumor gene expression data.* Elife, 2017. **6**.

16. Barbie, D.A., P. Tamayo, J.S. Boehm, S.Y. Kim, S.E. Moody, I.F. Dunn, et al., *Systematic RNA interference reveals that oncogenic KRAS-driven cancers require TBK1.* Nature, 2009. **462**(7269): p. 108-12.

17. Xu, L., C. Deng, B. Pang, X. Zhang, W. Liu, G. Liao, et al., *TIP: A Web Server for Resolving Tumor Immunophenotype Profiling.* Cancer Res, 2018. **78**(23): p. 6575-6580.

18. Thorsson, V., D.L. Gibbs, S.D. Brown, D. Wolf, D.S. Bortone, T.H. Ou Yang, et al., *The Immune Landscape of Cancer.* Immunity, 2018. **48**(4): p. 812-830.e14.

19. Mayakonda, A., D.C. Lin, Y. Assenov, C. Plass and H.P. Koeffler, *Maftools: efficient and comprehensive analysis of somatic variants in cancer.* Genome Res, 2018. **28**(11): p. 1747-1756.

20. Mermel, C.H., S.E. Schumacher, B. Hill, M.L. Meyerson, R. Beroukhim and G. Getz, *GISTIC2.0 facilitates sensitive and confident localization of the targets of focal somatic copy-number alteration in human cancers.* Genome Biol, 2011. **12**(4): p. R41.

21. Zhang, H., P. Meltzer and S. Davis, *RCircos: an R package for Circos 2D track plots.* BMC Bioinformatics, 2013. **14**: p. 244.

22. Jiang, P., S. Gu, D. Pan, J. Fu, A. Sahu, X. Hu, et al., *Signatures of T cell dysfunction and exclusion predict cancer immunotherapy response.* Nat Med, 2018. **24**(10): p. 1550-1558.

23. Roh, W., P.L. Chen, A. Reuben, C.N. Spencer, P.A. Prieto, J.P. Miller, et al., *Integrated molecular analysis of tumor biopsies on sequential CTLA-4 and PD-1 blockade reveals markers of response and resistance.* Sci Transl Med, 2017. **9**(379).

24. Maeser, D., R.F. Gruener and R.S. Huang, *oncoPredict: an R package for predicting in vivo or cancer patient drug response and biomarkers from cell line screening data.* Brief Bioinform, 2021. **22**(6).

25. Yang, C., X. Huang, Y. Li, J. Chen, Y. Lv and S. Dai, *Prognosis and personalized treatment prediction in TP53-mutant hepatocellular carcinoma: an in silico strategy towards precision oncology.* Brief Bioinform, 2021. **22**(3).
