# Supplementary material for: Advancing Prognosis Prediction and Immunotherapy Efficacy in Lung Adenocarcinoma Through Machine Learning: Novel Insights From Anoikis Regulator Patterns in Single‐Cell Multiomics
Source: Int J Genomics. 2026 Jan 3;2026:9458552. doi: 10.1155/ijog/9458552 (PMC12764181; doi:10.1155/ijog/9458552)
Supplement: Supplementary file 1 — Supporting Information 1 Figure S1: (A) Harmony algorithm reduced the batch effects of each sample in the combined scRNA‐seq cohort of LUAD. (B) Quality control of each cell type in the combined scRNA‐seq cohort of LUAD. (C) The expression profiles of anoikis‐related genes in the combined scRNA‐seq cohort of LUAD. (D) Harmony algorithm reduced the batch effects of epithelial cells in the combined scRNA‐seq cohort of LUAD. [file IJOG-2026-9458552-s001.pdf]

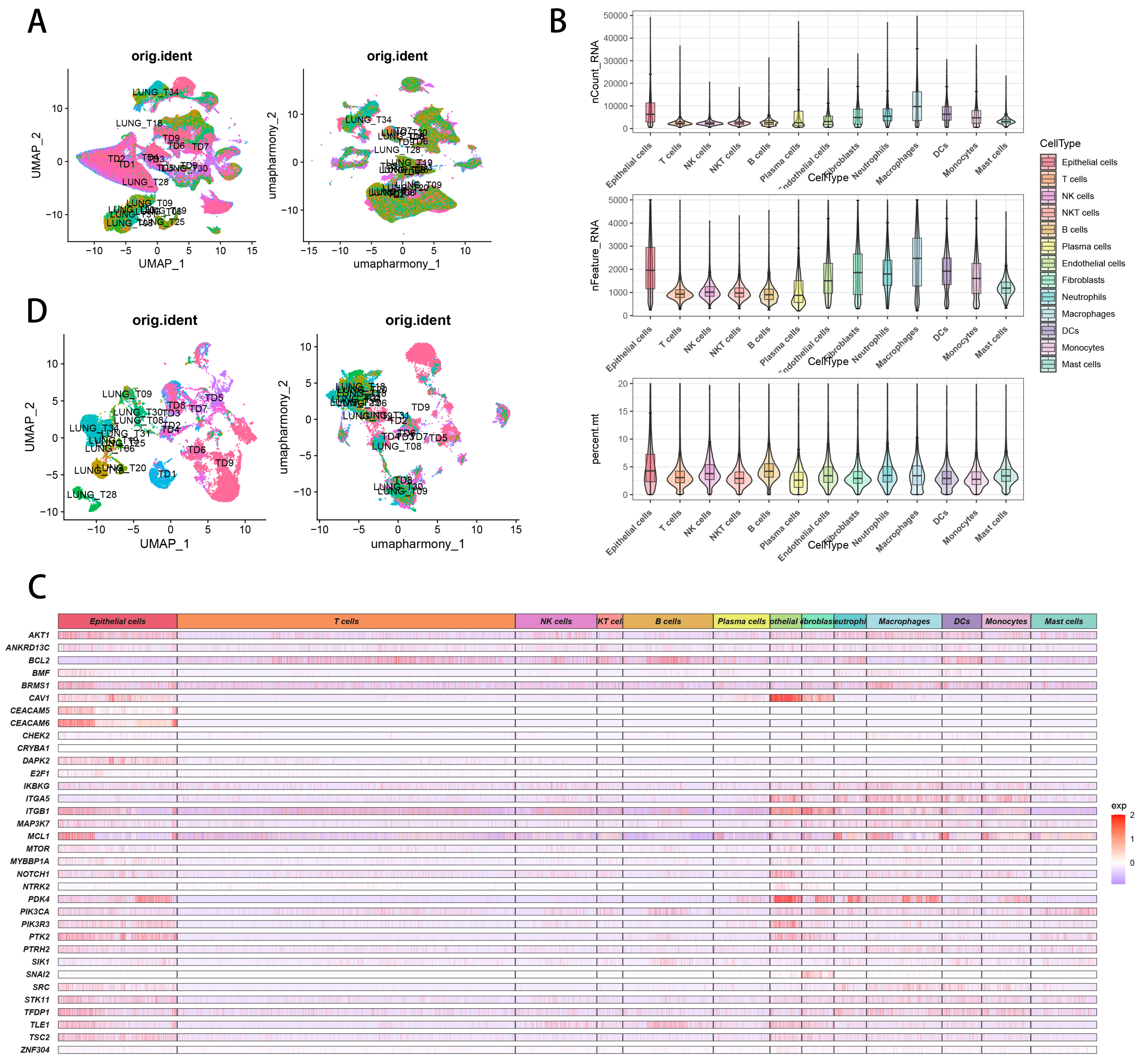
A
B
C
D
orig.ident
UMAP_1
UMAP_2
umapharmony_1
umapharmony_2
nCount_RNA
nFeature_RNA
percent.mt
CellType
Epithelial cells
T cells
NK cells
NKT cells
B cells
Plasma cells
Endothelial cells
Fibroblasts
Neutrophils
Macrophages
DCs
Monocytes
Mast cells
exp
AKT1
ANKRD13C
BCL2
BMF
BRMS1
CAV1
CEACAM5
CEACAM6
CHEK2
CRYBA1
DAPK2
E2F1
IKBKG
ITGA5
ITGB1
MAP3K7
MCL1
MTOR
MYBBP1A
NOTCH1
NTRK2
PDK4
PIK3CA
PIK3R3
PTK2
PTRH2
SIK1
SNAI2
SRC
STK11
TFDP1
TLE1
TSC2
ZNF304
